# Supplementary material for: “Like a doctor, like a brother”: Achieving competence amongst lay health workers delivering community-based rehabilitation for people with schizophrenia in Ethiopia
Source: PLoS One. 2021 Feb 25;16(2):e0246158. doi: 10.1371/journal.pone.0246158 (PMC7906313; doi:10.1371/journal.pone.0246158)
Supplement: S1 Appendix — (DOCX) [file pone.0246158.s001.docx]

**Supplementary file 1: RISE training outline**

Further training resources, including powerpoint slides and training activities in English and Amharic, are available on request [laura.asher@nottingham.ac.uk](mailto:laura.asher@nottingham.ac.uk)

|  | **Method** | **Session** | **Trainer** |
| --- | --- | --- | --- |
| **Week 1** |  |  |  |
| Monday AM | Classroom | Introduction to training | RISE team |
|  |  | Introduction to CBR |  |
|  |  | CBR for schizophrenia I |  |
| Monday PM |  | CBR for schizophrenia II |  |
|  |  | CBR for schizophrenia III |  |
|  |  | Overview of RISE pilot and trial |  |
| Tuesday AM | Classroom | Introduction to mental illness | Psychiatrist |
|  |  | Introduction to schizophrenia I |  |
| Tuesday PM |  | Introduction to schizophrenia II |  |
|  |  | Medicines for schizophrenia |  |
| Wednesday AM | Classroom | Disability | RAPID supervisor |
|  |  | Impact on family |  |
| Wednesday PM |  | Stigma |  |
|  |  | Human rights |  |
|  |  | Importance of the community |  |
| Thursday AM | Classroom | Initial visit | Psychiatrist |
|  |  | Communication skills |  |
| Thursday PM |  | Problem solving and trusting relationship |  |
|  |  | Needs assessment |  |
| Friday AM | Classroom | Goal Setting I | Psychiatrist |
|  |  | Goal setting II |  |
| Friday PM | Assessment |  | RISE team |
| **Week 2** |  |  |  |
| Monday AM | Classroom | Intro to RAPID | RAPID CBR worker/ supervisor |
| Monday PM | Practice in field | Needs assessment and goal setting |  |
| Tuesday AM | Discussion | Needs assessment and goal setting | RAPID supervisor |
| Tuesday PM | Classroom | Daily functioning |  |
|  |  | Improving physical health |  |
| Wednesday full day | Practice in field | Daily functioning & Improving physical health | RAPID CBR worker/ supervisor |
| Thursday AM | Discussion | Daily functioning & Improving physical health | RAPID supervisor |
| Thursday PM | Classroom | Family support groups |  |
|  |  | Dealing with stress and anger |  |
| Friday full day | Practice in field | Family groups & Dealing with stress and anger | RAPID CBR worker/ supervisor |
| Saturday AM | Discussion | Family groups & Dealing with stress and anger | RAPID supervisor |
| Saturday PM | Classroom | Community mobilisation I and II |  |
| Sunday AM | Classroom | Community life | RAPID supervisor |
|  |  | Getting back to work |  |
| **Week 3** |  |  |  |
| Monday AM | Practice in field | Community mobilisation | RAPID CBR worker/ supervisor |
| Monday PM | Discussion | Community mobilisation | RAPID supervisor |
| Tuesday full day | Day off |  |  |
| Wednesday full day | Assessment |  | RISE team |
| Thursday AM | Classroom | Understanding schizophrenia | Psychiatrist |
|  |  | Preparing for a crisis |  |
| Thursday PM |  | Risk assessment |  |
|  |  | Dealing with human rights issues |  |
| Friday full day | Practice in field | Understanding schizophrenia & Preparing for a crisis, risk assessment, human rights | Psychiatric nurse |
| **Week 4** | | |  |
| Monday AM | Discussion | Understanding schizophrenia & Preparing for a crisis, risk assessment, human rights | Psychiatrist |
| Monday PM | Classroom | Accessing healthcare |  |
|  |  | Supporting individuals to take medication |  |
| Tuesday full day | Practice in field | Accessing healthcare & Supporting medication | Psychiatric nurse |
| Wednesday AM | Discussion | Accessing healthcare & Supporting medication | Psychiatrist |
| Wednesday PM | Classroom | Family intervention |  |
|  |  | Dealing with distressing symptoms |  |
| Thursday full day | Practice in field | Family intervention & distressing symptoms | Psychiatric nurse |
| Friday full day | Day off |  |  |
| Saturday AM | Discussion | Family intervention & distressing symptoms | Psychiatrist |
| Saturday PM | Classroom | Taking control of your illness |  |
|  |  | Dealing with stigma |  |
| **Week 5** | | |  |
| Monday AM |  | Documentation | RISE team |
|  |  | Dealing with other problems |  |
| Monday PM | Classroom | Improving literacy |  |
|  |  | CBR worker wellbeing |  |
|  |  | Supervision |  |
| Tuesday full day | Practice in field | Taking control of illness & Dealing with stigma | Psychiatric nurse |
| Wednesday AM | Discussion  Classroom  Visit to health centre | Taking control of illness & Dealing with stigma | Psychiatrist  RISE team |
|  |  |  |  |
| Wednesday PM |  | Link to health centre |  |
|  |  | Dealing with difficult situations |  |
| Thursday full day | Revision |  | RISE team |
| Friday full day | Revision |  | RISE team |
