# Supplementary material for: “Like a doctor, like a brother”: Achieving competence amongst lay health workers delivering community-based rehabilitation for people with schizophrenia in Ethiopia
Source: PLoS One. 2021 Feb 25;16(2):e0246158. doi: 10.1371/journal.pone.0246158 (PMC7906313; doi:10.1371/journal.pone.0246158)
Supplement: S2 Appendix — (PDF) [file pone.0246158.s002.pdf]

RISE

Rehabilitation Intervention for people with Schizophrenia in Ethiopia

Manual for community-based rehabilitation workers

Version 2.0

Rehabilitation Intervention for people with Schizophrenia in Ethiopia (RISE): A manual for community-based rehabilitation workers

August 2017

Authors: Laura Asher, Charlotte Hanlon, Julian Eaton, Mary De Silva, Sudipto Chatterjee, Rahel Birhane, Atalay Alem, Vikram Patel, Abebaw Fekadu

The RISE manual was created following an extensive intervention development process in collaboration between the Department of Psychiatry, College of Health Sciences, Addis Ababa University, Ethiopia and the Department of Population Health, London School of Hygiene and Tropical Medicine, London, UK. For further information please see: http://journals.plos.org/plosone/article?id=10.1371/journal.pone.0143572. The RISE intervention was evaluated in a cluster randomised trial (Clinical Trials.gov NCT02160249)

Contact: laura.asher@lshtm.ac.uk

Acknowledgements: The authors would like to gratefully acknowledge several source materials that were used to develop the RISE manual, including 'The COPSI Manual: Manual for Working with People with Schizophrenia and their Families' (Sangath, Goa, India http://www.sangath.in/copsi/); the 'Mental Health Pocket Guide for Health Extension Workers' (Federal Democratic Republic of Ethiopia Ministry of Health); 'Community Based Rehabilitation Guidelines' (World Health Organisation); 'Facilitator's Guide for Psychosocial Rehabilitation Support Groups' (Carrie Brooke-Sumner, PRIME South Africa), 'Mental Health and Human Rights Resource Pack' (Amaudo UK, 2007), and 'Where there is no psychiatrist: A mental health care manual' (Vikram Patel).

Funding: The RISE project was funded by the Wellcome Trust. The RISE project was part of the PRogramme for Improving Mental health carE (PRIME), which is funded by the UK Department for International Development.

This manual belongs to:

Telephone number:

Contents

INTRODUCTION TO THE CBR MANUAL 6

SECTION A: UNDERSTAND WHAT SCHIZOPHRENIA IS AND THE PROBLEMS IT CAUSES 7

1 WHAT IS MENTAL ILLNESS? 8

2 WHAT IS SCHIZOPHRENIA? 13

3 MEDICATION FOR SCHIZOPHRENIA 18

4 DISABILITIES RELATED TO SCHIZOPHRENIA 21

5 IMPACT OF SCHIZOPHRENIA ON THE FAMILY 23

6 STIGMA AND DISCRIMINATION 26

7 HUMAN RIGHTS 29

8 THE IMPORTANCE OF THE COMMUNITY 31

SECTION B: LEARN HOW TO HELP PEOPLE WITH SCHIZOPHRENIA AND THEIR FAMILIES THROUGH CBR 34

9 OVERVIEW OF CBR DELIVERY 35

10 COMMUNICATION SKILLS AND PROBLEM SOLVING 50

11 CREATING A TRUSTING RELATIONSHIP 55

12 NEEDS ASSESSMENT 58

13 GOAL SETTING 62

14 RISK ASSESSMENT 68

15 BEING AWARE OF THE CAREGIVER'S NEEDS ... 73

16 MODULE: UNDERSTANDING SCHIZOPHRENIA AND ITS TREATMENT ... 75

17 MODULE: IMPROVING ACCESS TO HEALTH SERVICES ... 81

18 MODULE: PREPARING FOR A CRISIS ... 85

19 MODULE: DEALING WITH HUMAN RIGHTS PROBLEMS ... 89

20 MODULE: SUPPORTING INDIVIDUALS TO TAKE THEIR MEDICATION ... 96

21 MODULE: IMPROVING PHYSICAL HEALTH ... 106

22 MODULE: DEALING WITH DISTRESSING SYMPTOMS ... 112

23 MODULE: MANAGING STRESS AND ANGER ... 116

24 MODULE: IMPROVING DAY TO DAY FUNCTIONING ... 122

25 MODULE: IMPROVING THE FAMILY ENVIRONMENT ... 128

26 MODULE: TAKING PART IN COMMUNITY LIFE ... 134

27 MODULE: GETTING BACK TO WORK ... 140

28 MODULE: DEALING WITH STIGMA AND DISCRIMINATION ... 146

29 MODULE: IMPROVING LITERACY ... 150

30 MODULE: TAKING CONTROL OF YOUR HEALTH ... 152

31 COMMUNITY ENGAGEMENT ... 158

32 FAMILY SUPPORT GROUPS ... 167

SECTION C: KNOW HOW YOU WILL BE SUPPORTED TO DELIVER CBR 172

33 CBR WORKER WELLBEING 173

34 SUPERVISION 177

35 DEALING WITH DIFFICULT SITUATIONS.. 182

36 LINKS TO HEALTH SERVICES.. 195

37 GOOD DOCUMENTATION 198

38 HOW TO DEAL WITH OTHER PEOPLE WITH PROBLEMS................................ 200

39 SUPPORTING PEOPLE WITH INTELLECTUAL DISABILITY AND SCHIZOPHRENIA.............. 204

Introduction to the CBR manual

What is community-based rehabilitation (CBR) for schizophrenia?

Schizophrenia is a long-term illness, which can lead to severe disability in the individual, especially if the community does not accept them. The illness can also place a heavy burden on the family. People with schizophrenia usually need a period of rehabilitation and family support. Rehabilitation is where people are helped to regain skills and to return to their usual life activities.

Community-based rehabilitation is a way to help people with disabilities to be included in the life of their communities. It addresses all areas of a person's life including health, inclusion in social life and activities of the community, livelihood and work, education and empowerment. Empowerment means that the individual has the ability to make decisions about their own life. The focus is always on the needs and wishes of the individual. As a CBR worker you will work with individuals with schizophrenia, their families and their communities to help the individual to get back to their usual activities and be accepted fully into society.

Who is this manual for?

This manual is for community-based rehabilitation workers, who will be working with people with schizophrenia.

How to use this manual

This manual is designed to help you:

Understand what schizophrenia is and the problems it causes

→ this is covered in Section A

Learn how to help people schizophrenia and their families through CBR

→ this is covered in Section B

Know how you will be supported to deliver CBR

→ this is covered in Section C

Throughout the manual you will follow the experiences of Yosef and Sara, people with schizophrenia, and their families. Yosef and Sara are not real people, but their stories include situations and events that real people with schizophrenia have experienced.

SECTION A: Understand what schizophrenia is and the problems it causes

1 What is mental illness?

1.1 What does the mind do?

The mind does the following tasks:

- Perceiving: this means sight, hearing, smell, taste and touch
- Thinking: this means memory, judgement and decision-making
- Feeling: this means emotions such as sadness and happiness
- Behaviour: this means how we act

1.2 What is mental health?

Mental health means the mind is working well and we are productive, we have good relationships with others and we are able to cope well when problems happen.

1.3 What is mental illness?

Mental illness is any illness experienced by a person that affects their emotions, thoughts or behaviour, and has a negative effect on their life or the lives of their family.

1.4 What is disability?

Disability is where people have difficulty doing their normal activities and being part of their community. Many people with mental illness experience disability.

1.5 Why do people sometimes become mentally ill?

Like in other parts of the body, the mind can have problems and not work perfectly all the time. Mental illnesses are caused by problems with the way the mind works, especially when we are affected by stresses of life. Mental illness can cause problems with the four tasks of the mind: perceiving, thinking, feeling and behaviour.

1.6 Can people with mental illness recover?

It is important to remember that all people with mental illness have the possibility of recovery. Recovery does not always mean that the illness will be cured completely. Instead it means that things have improved, and disability is reduced, in a way that is important to the individual. In order to make sure individuals have the possibility to recover, it is important to always focus on their own needs and wishes.

1.7 What types of mental illness are there?

There are two main groups of mental illnesses: severe mental illness and common mental illness. Severe mental illness includes schizophrenia or psychosis. Common mental illness includes anxiety, depression and alcohol problems. Severe mental illness is usually more serious and disabling than common mental illness. Individuals may have more than one than one type of mental illness. For example, people with schizophrenia may also have an alcohol problem. In this manual you will learn lots more about people with schizophrenia, and how you can support them through CBR.

1.8 How can we recognise mental illness?

When people have mental illness they often behave in certain ways that helps us to recognise that the illness is there.

Psychosis or schizophrenia

Someone who has ANY of the following:	May have:
• Talks alone, says meaningless words or speaks in a strange language, talks very fast	**Psychosis or schizophrenia or 'severe mental illness'**
• Is often very restless; has mood swings from very happy or irritable to feeling very sad	
• Has false beliefs or suspicions, e.g. that someone is trying to harm them, or that they have special powers	
• Shows self-neglect (e.g. dirty, untidy appearance)	
• Is hearing voices or seeing things that are not there	
• Neglects or has difficulties in carrying out usual work, school performance, domestic or social activities	
• Behaves strangely e.g. running out of the house, running naked	

Seizures

Someone who has ANY of the following:	May have:
• Suddenly falls down and has sharp, shaky body movements (seizures)	**Epilepsy**
• During the seizure, he or she:	
– loses consciousness or does not respond normally	
–has stiff body, arms and legs	
–may bite their own tongue, injure themselves, and wet or soil their clothes	
• After the seizure: the person may feel very tired, sleepy, confused, and complain of headache, muscle aches	

Excessive sadness or worry

Someone who has ANY of the following:	May have:
• Complains of many physical symptoms but no physical cause has been found (e.g. headache, burning sensations, aches and pains)	**Depression or anxiety**
• Has low energy; is always tired; has sleep problems or does not want to eat	
• Always seems sad or anxious or irritable; feels hopeless or helpless or guilty	
• Has low interest or pleasure in activities that used to be enjoyable	
• Worries or thinks about day-to-day problems too much	
• Is not able or motivated to do their usual job, housework or social activities	
• Has been thinking of harming themselves, e.g. ending their life	

Alcohol problems

Someone who has ANY of the following:	May have:
• Often appears to be affected by alcohol (e.g. smells of alcohol, looks intoxicated, staggers when walking, slurred speech)	**Alcohol use disorder**
• Because of alcohol, often injures themselves, e.g. from falling down, fights, or walking in traffic	
• Has physical symptoms from excessive alcohol use (e.g. can't sleep, very tired, can't eat, vomits, complains of bad stomach, diarrhoea)	
• Has financial difficulties or crime-related or domestic problems	
• Has difficulties in carrying out usual work, school, domestic or social activities; does not attend or often arrives late	

Khat problems

Someone who has ANY of the following:	May have:
• Often appears drug-affected (e.g. low energy or agitated, fidgeting, slurred speech, suspicious, may see or hear things that are not real)	***Khat* or other drug use disorder**
• Shows signs of drug use, e.g. skin infection, unkempt appearance, burned lips, bad or worn teeth, has *dukak* (unpleasant dreams)	
• Has financial difficulties or crime-related legal or domestic problems	
• Has difficulties in carrying out their usual work, school, domestic or social activities; does not attend or often arrives late	

Problems with forgetfulness

An older person who has ANY of the following:	May have:
• Can't tell the time of day, or forgets where they are, forgets the names of objects or may not recognise familiar people	**Dementia**
• Gets lost when outside the home	
• Has difficult or embarrassing behaviour	
• Often loses emotional control, is easily upset, irritable or tearful	
• Lack of attention to personal hygiene, incontinence	
• Has difficulties in carrying out their usual work, domestic or social activities	

Child mental health problems

A child who has ANY of the following:	May have:
• Is usually kept in the house , and may be tied up or chained	**A childhood mental health or a developmental problem**
• People say the child behaves badly (e.g. is naughty, aggressive)	
• People say the child behaves oddly, or is possessed or cursed	
• Does not speak or respond like other children of the same age	
• Has problems dressing, feeding or washing themselves or using latrine at the usual age	
• Is always playing on their own, rocking, flapping their hands, or other odd behaviour	
• Is often being bullied or teased by other children	
• Is having problems at school or is often sent home by teachers	

1.9 What should we do when we recognise someone has mental illness?

You will learn what to do when you recognise someone with a mental illness other than schizophrenia in Chapter 38.

1.10 Summary

- Mental illnesses are caused by problems in the mind, which happen when people have lots of life difficulties and stresses
- Mental illness cause disability, which is when people find it difficult to do their usual activities
- Everyone with mental illness has a chance of recovery (getting better)
- There are different types of mental illness which have different signs

2 What is schizophrenia?

2.1 What kind of illness is schizophrenia?

Schizophrenia is a type of mental illness. People with schizophrenia have problems with the way their mind works in the following areas:

- Perceiving: To people with schizophrenia things that are not real might seem as if they are real
- Thinking: People with schizophrenia have confused or strange thinking. They believe things that are not true.
- Feeling: People with schizophrenia have fewer emotions or stronger emotions than usual
- Behaviour: People with schizophrenia may behave in a strange way.

2.2 What are the symptoms of schizophrenia?

There are four groups of symptoms of schizophrenia.

1. Hallucinations and delusions

Hallucinations are when a person experiences something that is not really happening. They may hear sounds that are not really there, for example a voice telling them to do something. They may see things, feel things or smell things that are not really there.

Delusions are when a person believes something that is not really true. For example, they may believe that they have special powers. Or they may believe that people are trying to poison their food.

Hallucinations and delusions may make people behave in a strange way. For example, they may talk to themselves, say meaningless words, speak in a strange language or speak very fast. They may also have mood swings, from very happy or irritable to feeling very sad.

2. Problems with motivation

People with schizophrenia often have low levels of motivation. They often do things slower, including thinking, talking and moving. They may feel they have no energy to do anything. They may not bother washing or dressing properly. They may not be interested in talking to other people. They may not show any emotions.

3. Problems with thinking clearly

People with schizophrenia often have problems concentrating on a task, or remembering things. This means it is difficult to follow a conversation or do household tasks such as cooking.

4. Lack of awareness of illness

Often people with schizophrenia do understand or believe that they are unwell. They may not realise that they are behaving strangely.

Yosef's story
Yosef is a 24 year old man who lives in a rural kebele with his mother, Addis, father, Solomon and younger siblings. About five years ago he was working hard on his family's farm. He had friends in village who he went to the alcohol shops with, or drank coffee. He wasn't wealthy but was seen as successful in the kebele. He always went to the kebele meetings and contributed to Edir. His mother hoped he would get married soon.<br>*About five years ago Yosef began to behave in a strange way. People in the kebele noticed that he talked to himself and laughed to himself. He confided in his mother that Saint Mary was communicating with him and told him that he had to do special tasks. Sometimes he ran away for several days. He began to believe that the neighbours hated him and were trying to ruin his crops. Sometimes he shouted at the neighbours and threatened them.*<br>*As time has gone on, he has stopped talking about his strange beliefs most of the time and he no longer runs away. But he is still not back to his usual self. He has stopped working on the farm and spends the days by himself. He stopped washing and dressing properly. Things don't seem to be improving for Yosef. Every few months he becomes much more unwell again. When this happens he seems to be very distressed and shouts at the neighbours again.*

2.3 What causes schizophrenia?

There is not one thing that causes schizophrenia and you cannot catch schizophrenia from someone else. **Schizophrenia is usually caused by a combination of vulnerability and life stress**. Protective factors can stop schizophrenia from developing or getting worse even when vulnerability and life stress are there. We don't always know why a person gets schizophrenia.

Vulnerability

Vulnerability means things that happen before a person is born or early in life. It includes:

- A person's mother having problems during her pregnancy, for example an infection.
- Having a difficult birth during which the baby is injured.
- Having a head injury.
- Using khat from an early age.
- Being abused during childhood.
- When someone has schizophrenia in the family most of the family members will not develop schizophrenia, but there is more chance of it happening.

Life stress

Life stress means events or circumstances in a person's life that put a lot of pressure on them. They might lead them to feel angry, anxious or sad. These might include:

- Being disappointed about a big thing. For example, a relationship or in work.
- Moving to an area where they don't know anyone
- Family conflict
- Illness
- Bereavement
- Having children

None of these things alone mean that a person will get schizophrenia. It only happens when the person has at least one type of vulnerability and at least one type of life stress.

Preventative factors

Preventative factors are things that help to stop a person becoming unwell with schizophrenia, or stop it from getting worse. They include:

- Good social support
- Good coping strategies
- Regular anti-psychotic medication

Yosef's story

Yosef's family believe that his problems are due to being possessed by spirits. His problems all seemed to start when he became very disappointed that he did not marry the woman he expected to. In the years before Yosef's problems started life had been stressful for the family. Some of his younger siblings had died and there had been a poor harvest a few years in a row.

2.4 What beliefs do people have about schizophrenia?

People with schizophrenia, their families and other people in the community may have beliefs about what causes schizophrenia which are different from the explanation given here. In Ethiopia the most common beliefs are that schizophrenia is caused by spirit possession, evil eye or possession by the devil. The same person may even have lots of different ideas about what causes it. Having different beliefs is not always a bad thing by itself. Having different beliefs becomes a problem when it results in:

- People with schizophrenia not getting treatment that works, for example not being taken to the health centre or hospital to get medication
- People with schizophrenia being treated badly, for example being beaten to exorcise the devil

2.5 How long does schizophrenia last?

Schizophrenia can either last a short time (even one episode), but usually lasts longer, even for many years. There are two main ways that people can be affected to schizophrenia:

- **Short-term, more severe illness**: During this phase the person can be very disturbed. They often have problems with hallucinations and delusions. This may lead the person to behave strangely. They may speak and think in a strange way, and may become angry. It is usually during this phase that the family of the person try and get help, for example by going to holy water or the hospital. The acute phase usually lasts from weeks to months.
- **Long-term, established illness**: During this phase the person may stop behaving strangely, but they still have problems with motivation and with thinking clearly. They may have problems with doing usual activities, such as getting dressed or doing farm work. They may find it difficult to talk to other people or socialise. This phase can last for months, years or can be life-long.

Most people with schizophrenia have a short-term, more severe illness at the beginning. What happens next varies a lot from person to person.

- A few people do not have any more severe illness. They may or may not have some less severe illness.
- Most people with schizophrenia have repeated episodes of more severe illness. These are known as relapses. In between they usually have some less severe illness.
- A few people stay unwell with more severe illness all the time

2.6 Can people recover from schizophrenia?

The important thing to remember is that everyone with schizophrenia has a chance to get better and to recover. Even if an individual does not get rid of all their symptoms, they have a good chance of getting back to their normal activities. Recovery can mean different things to different people. It doesn't usually mean that an individual is 'cured' of schizophrenia. Instead it means that things have improved in a way that is important to that person. Getting lots of support, taking medication and not using alcohol or khat make it more likely this will happen.

Even if a person with schizophrenia is feeling much better, they may suddenly become unwell again with a relapse. Relapses may happen due to not taking medication, physical illness, life stress or for no particular reason. The signs that a relapse is coming include problems with sleep, being isolated from people, being angry or anxious and stopping medication. In Chapter 30 you will learn how to prevent and manage relapses. Having a relapse doesn't mean they will be unwell forever and it is important for the individual and family not to give up hope that things will improve.

2.7 How is schizophrenia diagnosed?

There is no test for schizophrenia. The doctor or nurse decides whether someone has schizophrenia or not by listening to their problems and how long they have lasted.

2.8 How common is schizophrenia?

Schizophrenia is not a very common disease. In a kebele of 5000 people, there might be around 10 people with severe schizophrenia.

2.9 How do we give information about schizophrenia?

You will learn how to give information about schizophrenia in Chapter 16.

2.10 Summary

- People with schizophrenia have hallucinations, delusions, problems with motivation, problems with thinking clearly, and usually do not understand that they are unwell
- Schizophrenia is caused by a combination of vulnerability (early events) and life stress
- People with schizophrenia can have periods of short term, severe illness and long-term less severe illness
- All people with schizophrenia have the chance of recovery (getting back to usual activities)
- Schizophrenia is not very common

3 Medication for schizophrenia

3.1 What is anti-psychotic medication?

Taking regular anti-psychotic medication is an important part of treatment for all people with schizophrenia.

3.2 Why is it important to take anti-psychotic medication?

Taking anti-psychotic medication may improve the chances of recovery. Anti-psychotic medication is good at reducing the following symptoms of schizophrenia:

- Hallucinations (seeing or hearing things that aren’t really there)
- Delusions (believing things that aren’t true)

Anti-psychotic medication is less helpful for the following symptoms of schizophrenia:

- Problems with motivation, including showing emotions
- Problems with thinking clearly, including concentrating and being organised

3.3 When is anti-psychotic medication needed?

- When a person with schizophrenia is unwell, anti-psychotic medication is needed to reduce the symptoms
- Even when the person has become better and has no symptoms, medication is still needed to prevent relapse (i.e. prevent the person becoming unwell again).
- Antipsychotic medication works best when taken regularly but it can be difficult for patients to keep taking medication.

3.4 What types of anti-psychotic medication are there?

Anti-psychotic medications are available in tablet and injection forms (see Table 1). Injections are normally given when the person with schizophrenia has difficulty remembering to take their tablets or has experienced lots of relapses (when the severe illness comes back).

Table 1 Medications for schizophrenia

Name of medication	Brand name	Type of drug	How often it should be taken
Haloperidol	Haloperidol	Tablet	Once or twice a day
Chlorpromazine	CPZ	Tablet	Once or twice a day
Fluphenazine	Modecate	Injection	Fortnightly to monthly

3.5 What side effects do anti-psychotic medications have?

Medication can sometimes cause problems (side effects) as well as help (See Table 2). The side-effects from anti-psychotic medication are common so it is important that you know how to recognise them. Despite these problems, for many people their quality of life is better when taking the medication compared to when not taking it.

Table 2 Side effects of anti-psychotic medications

**Common**
The person feels restless and cannot sit still
The head, neck or body becomes stuck in an unusual position because of muscle stiffness
The hands shake
The person moves very slowly
The person is sleepy during the day
The mouth is dry
A lot of saliva is produced
Constipation
The person feels dizzy when they stand up too quickly from lying down or sitting.
The person wants to eat more than usual
Unusual movements of the head, neck, arms or legs
**Less common**
Seizures
Suddenly developing stiff muscles, fever, and confusion
Sexual problems
Skin rash
The person cannot pass urine

3.6 Can pregnant and breastfeeding women take anti-psychotic medication?

Depending on the woman, pregnant and breastfeeding women may need to stop or reduce anti-psychotic medication.

3.7 How do we help people with schizophrenia to take their medication?

You will learn how to help people with schizophrenia to take their medication regularly in Chapter 20, including how you can help with side effects.

Yosef's story

After several years of being unwell, Yosef's parents took him to the local health centre as they had heard he might be able to get help there. Here a nurse diagnosed him with schizophrenia. The nurse gave him anti-psychotic medication, called haloperidol. He was told to take the medication twice a day. Yosef found that taking the medication stopped the voice of Saint Mary in his head. His parents noticed that he stopped shouting at the neighbours and running away. However the medication also gave Yosef some problems. He is often very tired and drowsy and his hands often shake. These thinas make it hard to do farm work.

3.8 Summary

- Anti-psychotic medication is often needed to help people with schizophrenia to recover
- It is important to take medication when the individual is unwell, and to carry on taking the medication when the individual is well (to stop the illness coming back)
- Anti-psychotic medication can be given in tablets or injections
- Anti-psychotic medication can cause side-effects

4 Disabilities related to schizophrenia

4.1 What is disability?

Disability is when a person cannot do the activities that we would normally expect them to do, given their age and social circumstances. Disability can include:

- Problems with the body, for example blindness
- Problems with doing physical activities, for example walking
- Problems doing usual work and social activities, for example going to market.

4.2 What causes disabilities?

Disabilities in people with schizophrenia are caused by **a combination of the social environment in which the person lives and the illness itself.** For example,

- An individual may not be allowed to vote at the kebele meeting because of stigma and discrimination in the community (see Chapter 6).
- A person who doesn't have any family may not work because they are not being encouraged to do so.
- Problems with self-care may result from lack of motivation, a symptom of the illness.

4.3 What limitations do people with schizophrenia have?

People with schizophrenia usually experience many types of disability. These include:

1. Problems with self-care

This includes problems with washing, dressing, brushing hair and eating at the right time.

2. Problems doing household tasks

This includes problems with cooking, washing clothes, chopping wood or fetching water.

3. Problems with social interactions and participating in community life

This includes problems with having conversations with people and problems attending church, funerals or Edir meetings.

4. Problems in working

This includes problems doing farm work, trading or business.

5. Problems in looking after children

This includes problems giving children love, feeding and clothing them.

6. Problems with marital relationships

This includes not getting on well and arguing a lot.

4.4 What is the impact of having disabilities?

Disability may be more upsetting to the individual than the symptoms themselves. For example, finding it difficult to drink coffee with others may be more distressing than simply hearing voices. Disabilities may result in problems with money. For example, having problems with farm work is likely to mean the individual has less money. Disabilities may also have a big impact on the rest of the family. For example if a mother with schizophrenia finds it difficult to look after her children, other family members may need to help out. These family members may then have trouble looking after their own farm properly (see Chapter 5).

> ***Yosef's story***
>
> *Yosef does not often wash or dress himself. He no longer helps on the family's farm. He does not contribute to Edir anymore, instead he relies on his parent's contributions. He doesn't have any friends any more and does not go to Church.*

4.5 How do we assess disabilities?

You will learn how to assess what kinds of disabilities people with schizophrenia have as part of the Needs Assessment (see Chapter 12).

4.6 How do we work with individuals to improve their situation?

The aim of rehabilitation is to work with individuals to improve their situation so they are less disabled and can get back to their usual activities. Rehabilitation should be an empowering process. This means the needs and wishes of the individual are at the centre of the work. Rehabilitation isn't about 'doing things' to or for people who are disabled. It is about working together to improve their life and work towards recovery. Recovery can mean different things to different people. It doesn't usually mean that an individual is 'cured' of schizophrenia. Instead it means that things have improved in a way that is important to that person. You will see that all of CBR is focused towards rehabilitation.

4.7 Summary

- Disability is when an individual cannot do the activities we would normally expect
- Disability is caused by a combination of the social environment and the illness
- People with schizophrenia can have problems with: self-care, household tasks, participating in community life, working, looking after children and marital relationships
- Rehabilitation involves work with the individual to reduce disability.

5 Impact of schizophrenia on the family

As well as having a big impact on the individual, when a person has schizophrenia it also has a big effect on the whole family. This is because the family are usually their main carers. These are some of the effects the illness can have on the family.

5.1 Coming to terms with the illness

Not many families immediately know or believe that their relative has a mental illness. When the individual is very unwell, the family members may be scared by what is happening. When the episode is over, everyone wants to forget this painful time and focus on the future. Families may also look for other answers, hoping that the symptoms were caused by a physical problem or stressful events that can be removed.

5.2 Stigma and discrimination

Even when families know that their relative has a mental illness, they may not want to talk with others about it, because they fear other people's reactions or might be embarrassed. Other people in the community may suggest that there is something wrong with the family to cause the illness. The family may not want to invite anyone to the home. Or they may be anxious about leaving the individual at home alone. People with schizophrenia often find it difficult to get married. This is sometimes because of the stigma towards them, which may continue even if they become well. This puts an extra burden on the parents. See Chapter for more about stigma and discrimination.

5.3 Heavy responsibility of caring

Family members may spend a lot of their time looking after the person with schizophrenia. It is often female caregivers, either the individual's mother or wife, who take on most of the responsibilities. Their household routine may be disrupted and it may be difficult to attend social gatherings such as weddings. Sometimes caregivers, for example brothers and sisters, even decide not to marry so that they can take care of a person with schizophrenia. In the end the family may become isolated from the community. Some families may feel they do not get enough support from wider family members and the community. Families may be concerned that the individual will run away, harm their neighbours' property, or other people or get hurt themselves. This may mean that, as a last resort, families chain or tie up the individual

to protect them and other people. All of these issues mean the family members often become stressed, full of worry and exhausted.

5.4 Economic impact

People with schizophrenia usually need to take medication everyday for many months or years. The cost of this, along with the cost of seeing a nurse or doctor, and cost of transport to the health centre or hospital, means the family has to spend a lot of money on the individual. The person with schizophrenia may find it difficult to do their own farm work, day to day labouring or business. This means that the other family members have more work to do, and, there may be less money coming into the family. Also the family members may be able to do less work themselves, because they are carers.

5.5 Family conflict

Family members may not understand the illness and blame the individual for their bad behaviour and for not working. They may become frustrated and angry that the individual is not getting better. Conflict in families tends to make schizophrenia worse, and it is important to try to reduce this. The individual themselves may feel bad about all the money spent by the family on treatment.

> ***Yosef's story***
>
> *The living condition of Yosef's whole family has got worse because Yosef cannot work. Addis is often scared to leave Yosef alone in case he runs away again. This makes it difficult for her to do her usual work like going to market. His parents can't go to funerals or weddings unless he is asleep. They do not have any visitors at the house because they are ashamed of Yosef. They feel that nobody in the community helps them. Sometimes the whole family has arguments about Yosef. They argue about why he is not getting any better.*

5.6 How do we help the families of people with schizophrenia?

The whole of CBR aims to improve the situation of families, by helping individuals with schizophrenia get back to their usual activities. In particular, you will learn how to improve the family environment in Chapter 25.

5.7 Summary

- There may be extra costs to the family of a person with schizophrenia and they may have problems doing usual activities
- The family may experience stigma
- There may be conflict within the family

6 Stigma and discrimination

6.1 What is stigma?

Stigma is when people automatically think bad things about a person just because they have a mental illness. Stigma from other people towards people with schizophrenia consists of three problems.

- The problem of knowledge. This is called ignorance.
- The problem of attitudes. This is called prejudice.
- The problem of behaviour. This is called discrimination.

All these types of stigma can be found in family members, community members and health workers. Stigma from the person with schizophrenia towards him or herself, because they start to believe the negative things that other people say, is called self-stigma. Stigma and discrimination is also experienced by other people, for example people with physical disabilities, albinism, HIV or epilepsy.

6.2 What types of stigma do people with schizophrenia experience?

Ignorance

Most people in the community and some health workers do not have a good understanding of schizophrenia. This means they can have some of the following false beliefs:

- Nobody recovers from schizophrenia
- There is no treatment for schizophrenia
- People with schizophrenia are violent and dangerous
- People with schizophrenia are lazy and you cannot trust them
- Schizophrenia is the result of spirit possession
- Schizophrenia is the result of a weak character
- Everything people with schizophrenia say is nonsense
- People with schizophrenia cannot make decisions about their own lives

Prejudice

Prejudice is when people feel emotions such as anxiety, anger, hostility or disgust towards people with schizophrenia, as well as having ignorant thoughts. For example, a neighbour may feel scared whilst talking to a person with schizophrenia, even if the person is not acting in an aggressive way.

Discrimination

Discrimination is when people behave differently towards people with schizophrenia, because of their ignorance and prejudice. This can mean that individuals are not able to do the activities that they used to when they were well. People with schizophrenia may continue to be discriminated against even though they have recovered. They often feel that discrimination is more distressing than the symptoms of the illness.

Here are some examples of discrimination which people with schizophrenia experience:

- Community members do not greet the individual in the neighbourhood
- Community members call the individual rude names, laugh or gossip about them
- Friends do not want to talk or drink coffee with the individual
- Community members do not listen to the individual when they try to contribute at community meetings, such as kebele meetings or edir meetings
- The individual cannot get a job or cannot be involved in a microfinance group because employers or group members think they are lazy and unreliable
- The individual finds it difficult to get married
- The family asks the individual to hide when relatives visit the house
- Family members may tie up the person with schizophrenia. This is usually done because the person is very unwell and the family cannot control them. However sometimes it is done because the family believes the individual is possessed by spirits or the devil.

Self-stigma

Some people with schizophrenia start to believe the negative attitudes that others have towards them. This can result in:

- Low self-esteem
- Feeling critical towards themselves
- Feeling hopeless
- Depression
- Being isolated

People with schizophrenia may stop themselves doing activities because they expect people to be rude to them, laugh at them, or treat them differently.

6.3 What types of stigma do families experience?

The family members of people with schizophrenia commonly experience stigma too. The whole family may be treated with less respect or people may avoid them. They may also be blamed for the illness in the individual.

> ***Yosef's story***
>
> *Sometimes people laugh and gossip about Yosef when he goes outside. Sometimes children throw stones at him. Yosef doesn't have any friends anymore. His family doesn't think he will ever get married now he has become ill. Once when he was feeling better he went to a kebele meeting and tried to add to the discussion. The other people there didn't say anything bad to him, but they ignored him. This made Yosef feel bad and he didn't go the meeting again. He has started to believe other people and thinks he is worthless.*

6.4 How do we reduce experiences of stigma?

You will learn how CBR can reduce experiences of stigma and discrimination in Chapter 28.

6.5 Summary

- Stigma includes problems of knowledge (ignorance), attitudes (prejudice) and behaviour (discrimination).
- Many people with schizophrenia, and also their families, experience stigma
- Experiences of stigma may include: being laughed at or called names, being excluded from community activities or work, and being kept hidden or tied up

7 Human rights

7.1 What are human rights?

The term ‘human rights’ describes the belief that all people have basic rights, including the right to life, freedom of speech, belief and freedom from fear. The Ethiopian constitution states that human rights and freedom are in our human nature (Ethiopian constitution, chapter 10, No 1). All people have human rights, regardless of age, sex, ethnicity or religion. These rights enable a person to live their life with worth and dignity. The Ethiopian Constitution states that human rights should not be violated.

7.2 What human rights do people with mental illness have?

People with mental health problems have equal rights like any other Ethiopian citizen; they have the same need for respect and care. The following rights apply to people with mental illness, just like all other people:

- They should not be chained, suffer, or be locked up at home because of their illness
- They should not be held by police just because they are ill
- They have the right to have a family of their own, get married and have children;
- They have the right to work
- They have basic rights for food, clothing, housing and medical services; they should not be left to starve, or be homeless.
- They should not be abused, disrespected or called bad names, or beaten to drive out bad sprits.

It is quite common for people with mental illness to be denied their human rights.

Yosef’s story

When Yosef becomes very unwell his parents sometimes chain him to the wall of their house. They do this because they are worried he will run away and hurt someone, or get eaten by hyenas or drown in the river.

7.3 How do we deal with human rights problems in people with schizophrenia?

In Chapter 19 you will learn how to help protect the human rights of individuals.

7.4 Summary

- All people have the same Human rights including the right to life, freedom of speech, belief and freedom from fear.
- It is quite common for people with schizophrenia to be denied their human rights

8 The importance of the community

8.1 What is the community?

Everybody lives within a community of some kind. When we say community we mean:

- A group of people living in the same place, for example the kebele
- A feeling of shared attitudes and interests

For people with schizophrenia their community might include:

- Their friends, neighbours and relatives
- Community members that live in the same kebele, even if they do not know them
- Community leaders such as kebele leaders, priests or the headteacher

There are many community resources in every kebele. These include:

- Churches and mosques, including priests
- Edir groups
- Religious groups such as mahaber, tsewa and lika
- Primary school
- Women's Associations and Youth Associations
- Kebele administration
- Markets
- Traditional healers, for example tanqway or herbalists, and holy water sites and holy water priests

8.2 What problems do people with schizophrenia have in community life?

People with schizophrenia often find they have problems accessing the community resources and doing their usual community activities (see Chapter 26). These might include:

- Not socialising with friends and neighbours, for example drinking coffee
- Not attending church or mosque
- Not participating in religious groups such as mahaber
- Not participating in Edir
- Not attending community groups such as the Women's or Youth Association

8.3 What role does the community have in the life of the person with schizophrenia?

Positive influences

The community can have a positive or supportive influence on the person with schizophrenia, such as:

- Making the individual feel welcome when they participate in community activities
- Helping the individual and family with food or other practical support when they are having a particularly difficult time
- Giving emotional support to the family, for example listening to their problems
- Organising community activities that the person with schizophrenia may wish to be part of. This includes government schemes, such as adult literacy groups, and local groups, such as Edir.
- Helping the family in transporting the individual to the health centre or hospital

Negative influences

The community can also have a negative influence on the person with schizophrenia. The community may make it even more difficult for the individual to participate in their usual activities. This is usually due to stigma and discrimination. See Chapter 6 for more detail.

8.4 What is the role of the community in CBR?

CBR aims to increase the positive influences of the community, and reduce the negative influences. It also aims to help people with schizophrenia to access the community resources. The community also has an important role in making sure the positive changes that have been made during CBR are continued after the CBR worker has left (see Chapter 31). The community also benefits through CBR. Once the individual is back to doing their usual activities, they are likely to be a more productive and active community member.

Yosef's story

Yosef used to be an active member of his kebele. The kebele chairperson thinks the community has been affected by his illness, and if Yosef could get better the community would benefit.

8.5 Summary

- People with schizophrenia often have problems taking part in community life
- The community can have a positive or negative influence on the person with schizophrenia

- The aim of CBR is to increase the positive influences and reduce the negative influences of the community.
- CBR helps individuals to become more productive and active, so the community also benefits

SECTION B: Learn how to help people with schizophrenia and their families through CBR

9 Overview of CBR delivery

9.1 What is community-based rehabilitation?

Community-based rehabilitation is a way to help people with disabilities to be included in the life of their communities. It aims to improve the quality of all areas of a person's life. The focus is always on the needs and wishes of the individual. CBR benefits the family, by reducing the burden upon them. It also benefits the whole community, by helping the individual to be a more active and productive community member. Sometimes it takes a long time to see the positive impacts of CBR, so it is important that everyone involved is patient and stays positive.

9.2 What are the principles of CBR?

1. Use a holistic approach

CBR addresses all aspects of the person's life including:

- Social
- Livelihood
- Health
- Education
- Empowerment (a person's ability to make decision about their own life)

These are all areas where the individual might be having problems and where CBR can make a positive change.

2. Work with the family and the community.

The family are the main carers for people with schizophrenia and the aim is to hand over care to family at the end of your involvement. The community also has an important role in the individual's life. Working with the community is required to maximise the impact of the family work and to ensure the positive impact of CBR stays after you have gone.

3. Encourage respect for human rights

All of CBR, whether work with the individual, family or community, should encourage respect for the human rights of the individual. Treating people with schizophrenia with dignity and respect is empowering and will make it more likely they will get back to their usual activities.

4. Link to existing services

Where possible, should involve linking the individual to existing services, for example the government run adult literacy scheme.

9.3 Who is involved in CBR?

The person with schizophrenia

The person with schizophrenia should always be at the centre of CBR. By always focusing on their concerns, needs and wishes you will be more likely to help them to recover in a way that is important to them. In this manual we will usually refer to the person with schizophrenia as 'the individual'.

Primary caregiver

The primary caregiver is the main person who looks after the person with schizophrenia. This may be a spouse, parent, sibling or other relative. Very occasionally the primary caregiver may be a neighbour, friend or other community member. The primary caregiver should be your main point of contact within the family. They should also be present at all home visits. They will have been identified before you start the CBR.

Other family members

At your first visit you should find out which other members are involved in the care of the person with schizophrenia. This may include a spouse, parent, sibling, child or grandparent. Depending on the individual circumstances, other family members may be involved in many of the home visits or only a few. The manual will tell you when it is particularly useful to involve other family members. In this manual we will often refer to the primary caregiver and other family members as 'the family'.

You, the CBR worker

You will arrange and lead all the home visits to the family and do all of the community work.

Your supervisor

You will be supported by your supervisor. Your supervisor will come with you at certain home visits, for example for the needs assessment and goal setting. You will also meet regularly with them to discuss the progress of each individual (see Chapter 34).

Health Centre staff

You will be linked to one health centre, where all the people with schizophrenia you are working with will receive their medical care. You will need to go with the individual to the health centre every few months, around the time of each CBR Review. There are several situations when you should send the individual for a review at the health centre (see Chapter 37).

Trial psychiatric nurse

In certain circumstances you or your supervisor may need to contact the psychiatric nurse. This will usually be when a serious event has occurred, such as the individual attempting suicide. There is more detail on when to contact the psychiatric nurse in Chapter 35.

Community leaders

Community leaders will be invited to attend awareness-raising meetings. You may also need to have individual meetings with certain community leaders to help with parts of CBR.

Community members

Community members will be invited to attend public awareness raising events.

9.4 Where and when does CBR take place?

Most of CBR takes place through home visits, where you will meet with the individual and family. You should try to fit the location, duration and frequency of the home visits around the needs and wishes of the individual and family. These visits will usually last between 30 and 90 minutes, but may be shorter if necessary. In some cases the individual or family may not feel comfortable for you to visit them at home. At the first visit you should offer to see them at the health post instead, if they wish. Wherever you meet the individual, the caregiver or another family member should always be present or nearby. How often you make the home visits are depends on the phase. You will learn about the Phases in section 9.7. We suggest the following, but this may vary between families or during harvest time or festivals:

Phase I: Every week or every 15 days

Phase II: Every 15 days

Phase III: Every month.

The community work will take place at different places in the kebele. For example, you may meet the kebele leader at the kebele office.

9.5 How long does CBR last?

You will work with each individual for 12 months. Even if the individual becomes much better whilst you are working with them, you should still continue to visit them during the 12-month period. This will help them to keep well and develop more skills.

9.6 How do we start CBR (initial visit)?

Your supervisor will give you the name and contact details of the individuals you will be working with. You will arrange the first home visit by phone or in person. You can ask the Health

Extension Worker to help you to find the home of the individual. Over the first one or two visits you should give the following information:

- Introduce yourself
- Describe the structure and purpose of CBR (see section 9.1 and 9.7). Explain that you are not able to offer money, a loan or a job. Explain that the family do not need to pay you or give you gifts.
- Describe the modules you might cover (see section 9.7)
- Discuss confidentiality (see Chapter 10, section 10.6)
- Explain that the caregiver or another family member should be present or nearby when you do the visits

You should also gather the following information:

- Ask why they decided to participate in CBR
- Ask how long they have been unwell and when they first sought help
- Ask if they are generally well or unwell at the moment. Ask what problems the illness is causing at the moment e.g. behaving strangely, being unmotivated.
- Relationship of primary caregiver to individual
- Other family members who will also be involved in CBR. In particular think about which family member/s have most power and influence. This may be an older, male relative.
- Home environment including who lives in the home, the type and condition of the home, position of the home and any issues with access, and income and land owned by the family.
- Preferred location of visits (home or health post)
- If the individual is currently chained
- If the individual is currently accessing the health centre
- If the individual is currently accessing medication
- Contact details of the primary caregiver
- Urgent needs which need addressing immediately, before the next home visit. For example, the individual is suicidal or seriously ill with a physical health problem.
- The particular strengths, opportunities and challenges for that family.

You should record this information on the Initial Assessment form (Form 1).

9.7 What are the phases of CBR?

This CBR is divided into three phases. At each phase you will be helping the individual in different ways. This is because as time passes people with schizophrenia will usually have some improvement, so their needs will change. At the beginning of each Phase you will conduct a CBR Review together with your supervisor. The key areas for each phase are described below and summarised in Table 3.

Phase I

During Phase I the aim is to get to know the individual and family and to develop a trusting relationship with them. You will complete the four Phase I modules with all individuals. These modules are important for all people with schizophrenia as they address basic needs:

- Understanding schizophrenia and its treatment
- Improving access to health services
- Dealing with human rights issues, when they arise
- Preparing for a crisis

CBR Review I, at the beginning of Phase I, helps to decide which order to address these modules. The length of Phase I depends on the particular circumstances of the individual. It may last for around three months, but it may be much shorter or much longer. At the end of Phase I you may wish to revise the main issues you have covered before moving to Phase II.

Phase II

Aside from the basic needs addressed in Phase I, people with schizophrenia have very different disabilities and needs. In CBR Review II, at the beginning of Phase II, you will therefore do a Needs Assessment. This helps you to decide which areas to focus on. The Needs Assessment is described in Chapter 12. On the basis of the Needs Assessment and the wishes of the individual and family, you will set goals to work towards during Phase II. Goal Setting is described in Chapter 13. To make sure we are taking actions to achieve these goals, you will then deliver a series of linked Modules. The exact modules that you deliver to the individual will depend on which problems have been identified in the Needs Assessment. Depending on the needs of the individual Phase II may include the following modules:

- Supporting individuals to take medication
- Improving day to day functioning
- Improving the family environment
- Managing stress and anger

- Taking part in community life
- Getting back to work
- Dealing with stigma and discrimination
- Dealing with distressing symptoms
- Improving literacy
- Improving physical health
- Taking control of your health

In Phase II you will also set up a Family Support Group in each kebele. All caregivers, and in some cases people with schizophrenia, are invited to these Groups (see Chapter 32). The length of Phase II depends on the particular circumstances of the individual. It may last for around 4 to 6 months, but it may be much shorter or much longer. At the end of Phase II you may wish to revise the main issues you have covered before moving to Phase III.

Phase III

The new focus in Phase III is prevention of relapse (stopping the illness returning). Therefore the Module that we cover with everyone in Phase III is:

- Taking control of your health

The main purpose of Phase III is to maintain the progress made in Phase II. However, individuals will still have different needs, and these may have changed over time since Phase II. In CBR Review III at the beginning of Phase III you will therefore do another Needs Assessment. Depending on the needs of the individual, and the goals that are set, Phase III may include any of the Phase II modules. The Family Support Groups should continue through Phase III. The length of Phase III depends on the particular circumstances of the individual. It may last for around 3 to 6 months, but it may be much shorter or much longer.

Table 1 Overview of RISE CBR structure

Phase	CBR Review	Goals	Modules	Community engagement tasks	Family Support Group
I	CBR Review I:<br>• Initial Assessment<br>• Needs Assessment<br>• Goal setting for Phase I<br>• Risk Assessment<br>• Accompany to health centre<br>• Rehabilitation plan	Individual and caregiver have been informed of what schizophrenia is, available treatments, and the potential for recovery	Understanding schizophrenia and its treatment	Task 1: Meet with health extension worker/s<br>Task 2: Identify key community leaders<br>Task 3: Identify key community resources<br>Task 4: Ascertain what community engagement relating to mental illness has already taken place or is planned<br>Task 5: Meet with key community leaders<br>Task 6: Community awareness-raising<br>Task 7: Identify potential employment opportunities in the kebele	
		Individual is able to access medication and attend health centre for mental health as indicated by clinical status	Improving access to health services		
		Crisis management plan is in place	Preparing for a crisis		
		Person with schizophrenia is not chained or restrained	Dealing with human rights issues		
II	CBR Review II:<br>• Needs Assessment<br>• Goal setting for Phase II<br>• Risk Assessment<br>• Accompany to health centre<br>• Invite to Family Support Group<br>• Rehabilitation plan	Individual is willing to take medication	Supporting individuals to take medication	Task 8: Individual meetings with Kebele leaders<br><br>Task 9: Individual meetings with Edir leaders<br><br>Task 10: Individual meetings with religious leaders<br><br>Task 11: Individual meetings with traditional healer/ holy water priest/ attendant	Support group active
		Individual has strategies to remember to take medication			
		Individual feels side effects are improving			
		Individual feels hallucinations and delusions are improving	Dealing with distressing symptoms		
		Individual feels problems with motivation and thinking clearly are improving			
		Individual is able to access health services for physical and sexual health needs and contraception when	Improving access to health services		

		required		Task 12: Demonstrate progress of client/s to community leaders/ wider community<br><br>Task 13: Community awareness raising consolidation<br><br>Task 14: Facilitate employment opportunities in the kebele<br><br>Task 15: Individual meetings with literacy group leader	
		Individual has strategies to deal with stress and anger	Managing stress and anger		
		Individual has information to make decisions about health-related behaviours	Improving Physical Health		
		Individual has good physical, sexual and reproductive health			
		Individual is not malnourished			
		Individual participates in community life	Taking part in community life		
		Individual participates in religious activities if they are important to the individual			
		Person with schizophrenia is able to interact socially with neighbours and friends			
		Individual has improving ability to do parenting activities	Improving the family environment		
		Individual can carry out usual family role			
		Individual has improved relationship with family members			
		Caregiver has improved ability to cope			
		Individual has improving self-care	Improving day to day functioning		
		Individual has improving ability to do household tasks			
		Individual has improving self-esteem	Dealing with stigma and		

		Individual does not feel discriminated against	discrimination		
		Individual is not the victim of physical, sexual or emotional abuse	Dealing with human rights issues		
		Individual has restored participation in livelihood activities, including farm work	Getting back to work		
		Individual has basic literacy skills	Improving literacy		
**3**	CBR Review III:<br>• Needs Assessment<br>• Goal setting for Phase III<br>• Risk Assessment<br>• Accompany to health centre<br>• Rehabilitation Plan	Individual has relapse prevention plan	Taking control of your health	Any Phase 2 Community Engagement task	Support group active
		Any Phase 2 goal	Any Phase 2 module		

9.8 What are the CBR Reviews?

The CBR Reviews are a chance for you to discuss the individual's progress with the individual, the family and your supervisor. They take place at the beginning of each Phase. What you cover in the CBR Review depends on the Phase. Just before or around the time of each CBR Review you should go with the individual to the health centre. By doing this you will get more information about the progress the individual is making and any problems they are having. Every time you accompany an individual to the health centre you should record it on the Health Centre Contact Form (Form 2). Each CBR Review will usually take more than one home visit to complete. You should complete the CBR Review Form (Form 3) to ensure you have done everything for that CBR Review.

CBR Review I: Beginning of Phase I

Initial Assessment (see section 9.6 and Form 1)

Needs Assessment (see Chapter 12 and Form 4)

Accompany to health centre (see Form 2)

Prioritise Phase I Goals (see Section 13.5 and Form 5)

Risk Assessment (see Chapter 14 and Form 7)

Rehabilitation Plan (see Form 8)

CBR Review II: Beginning of Phase II

Needs Assessment (see Chapter 12 and Form 4)

Goal setting for Phase II (see Section 13.5 and Form 6)

Risk Assessment

Accompany to health centre

Invite to Family Support Group (see Chapter 32)

Rehabilitation Plan

CBR Review III: Beginning of Phase III

Needs Assessment

Goal setting for Phase III (see Section 13.5 and Form 6)

Risk Assessment

Accompany to health centre

Rehabilitation Plan

CBR Review IV: At 10-11 months

Continuing Care Assessment (see Section 9.12 and Form 9)

Accompany to health centre

Yosef's story

Berhan is a CBR worker who is asked to look after Yosef and his family. She records her first visit to Yosef on the Initial Assessment Form. At the next visit she conducts the Needs Assessment to get to know his situation better. As part of CBR Review I, Berhan also goes to the health centre with Yosef and Addis a few days later and sits in whilst Yosef sees the nurse. This helps her to understand his illness. She records the information on the Health Centre Contact Form.

9.9 What community work is involved?

In every kebele, you need to conduct the same core tasks to make links with the community (community engagement tasks). These should be completed around the time when the individuals are in Phase I (around the first three months):

- Task 1: Meet with health extension worker/s
- Task 2: Identify key community leaders
- Task 3: Identify key community resources
- Task 4: Find out what community work relating to mental illness has already taken place or is planned
- Task 5: Meetings with key community leaders
- Task 6: Community awareness-raising event/s
- Task 7: Identify potential employment opportunities in the kebele

Depending on the needs of the people with schizophrenia, you may also need to conduct the following tasks when the individuals are in Phases II and III:

- Task 8: Individual meeting with Kebele leaders
- Task 9: Individual meeting with Edir leaders
- Task 10: Individual meeting with religious leaders
- Task 11: Individual meeting with traditional healer/ holy water priest/ attendant
- Task 12: Individual meeting with literacy group leader
- Task 13: Demonstrate progress of client/s to community leaders/ wider community
- Task 14: Community awareness raising consolidation
- Task 15: Facilitate employment opportunities in the kebele

The community engagement work should be recorded in the Kebele Logbook (Form 10)

9.10 How do you deliver CBR?

CBR Manual

The rest of the manual will tell you how to deliver each module of CBR. Each chapter in the manual covers a different module. In each chapter you will find the following questions are answered:

- What is the problem?
- Why does this problem happen?
- Why is it important to help with this problem?
- How can we help?

Chapter 31 also tells you how to conduct the Community Engagement tasks.

Step-by-step guide

There is a short step-by-step guide to delivering each module. These are designed to take on the home visits to remind you of the main things to say and do for the module.

Structure of home visits

Each home visit should cover the following

- Check their general health and symptoms
- Check and record progress relating to ongoing goals
- Problem solving for ongoing goals
- Deliver module/s: selected on the basis of the remaining goals
- Check whether they are taking medication regularly
- Do brief risk assessment (see Chapter 14)
- Tasks to complete before the next session
- Plan date and content of next session

Recording home visits

All home visits should be recorded on the Home Visit Form (Form 11). You should also complete the Visit Summary Form (Form 12), which asks for information about the practical aspects of your visit, for example how long it took you to get there.

Completing modules

You can take use more than one home visit to complete a module. Sometimes, you may also cover more than one module in one home visit.

Workload

You should usually do two tasks every day. For example two home visits, or one home visit and one meeting with community leaders. See Form 13 for an example of how your timetable might look.

9.11 How do you encourage individuals to take part in CBR?

When you are going to make a home visit, if possible call ahead the day before or when you are on your way to remind the individual or caregiver. During Phase III you should also call the individual or caregiver to check their progress every two weeks. If the individual goes missing for a long period you should encourage the family and community members to look for the individual, but you should not normally search for them yourself.

9.12 How do you end CBR (continuing care assessment)?

At around 10 or 11 months you should conduct CBR Review IV. At this CBR Review you should discuss the fact that CBR will soon end with the individual and family. You should look back to the Goal Setting Forms from each Phase and discuss the progress that has been made on each goal. This should be a positive exercise, which focuses on the individual's strengths and achievements. It should not be a chance to make the individual feel that they have failed. Around this time you should also attend the health centre with the individual. Together with your supervisor you should complete the Continuing Care Assessment (see Form 9). The aim is to identify ongoing needs and to formulate an ongoing plan to address these needs once you have left. You should prepare the individual and family for the possibility that old problems will come back or that new problems may appear. Reassure them that the health centre staff will continue to look after the individual. At the final session the Continuing Care Plan should be reviewed with the individual and their caregiver. They may feel angry or upset that the home visits are ending. Remind them that they have learnt to deal with their own problems whilst you have been working with them.

9.13 What do you do if CBR is not wanted?

Rarely the individual and/or family may wish to stop participating in CBR before the end of 12 months. This may make you feel shocked, upset or rejected. However we should not force anyone to be involved in CBR if they do not want to be. Here are some ways to deal with the situation:

- Accept what the family says and don't try to argue with them

- Check what the family is unhappy with and try to change it. For example, offer to meet them at the health post instead of at home.
- Consider inviting an individual who has received or is receiving CBR to discuss the benefits of participating with the individual and family.
- Always discuss the situation with your supervisor
- Leave your contact details with the family. Say you are happy to continue whenever they are ready.
- Try to arrange a final session to say goodbye and do some final work- for example reminding them what to do in an emergency situation.

  In some cases only the caregiver does not wish to participate in CBR or is too busy, but the individual wishes to continue. When this happens you may continue to support the individual, but only if you and your supervisor have no concerns about your safety.

9.14 Summary

- CBR aims to improve the lives of people with schizophrenia and involve them in community life. The principles are: employ a holistic approach, work with the family and the community, encourage respect for human rights and link to existing services.
- The following people are involved in CBR: the person with schizophrenia, the caregiver and family, the CBR worker, the CBR supervisor, the health centre staff, the psychiatric nurse, the community leader and community members.
- CBR lasts 12 months for each individual and takes place through home visits and meetings in the community
- CBR has 3 phases. In Phase I the aim is to build a relationship with the individual and cover the same Modules with everyone: Understanding Schizophrenia and its treatment, Improving access to health services, Dealing with human rights issues, Preparing for a crisis.
- In Phases II and III the Modules differ between individuals depending on their needs. They might include: support with taking medication, improving daily functioning, improving the family environment, getting back to work and community life, Dealing with stigma and distressing symptoms, improving physical health and literacy.
- The Community Engagement Work involves: Identifying community resources, engaging with the health extension worker and community leaders, doing community awareness-raising and arranging employment opportunities.
- You will set up a Family Support Group for families to provide mutual support

- The CBR manual and Step by Step Guide tell you how to deliver CBR

10 Communication skills and problem solving

10.1 Why do we need communication skills?

A lot of CBR takes place in the family home through discussions between you and the person with schizophrenia and their family. For these discussions to go well and be useful you will need certain skills in listening and communicating. Although it may sometimes be more difficult to communicate clearly with a person who has schizophrenia, it is important that you always treat the person as a responsible and respected individual.

10.2 How to communicate well with families

1. Be friendly and understanding

It is important to be warm and friendly towards the individuals and family. Try to put yourself in the place of the person you are talking to and feel what he or she could be feeling at the moment, for example frustration or anger. The process will help you to understand better the situation of the person. You can also let the person know that their feelings are common and expected for someone in their situation. This is different from expressing pity where you only express the fact that you feel bad for the person. This does not make the person feel that he or she is being genuinely understood. Some ways to be understanding include: "I can understand how the way people have been treating you makes you feel angry" and "You seem to feel upset today."

2. Be non-judgemental

You should accept the person for who he or she is irrespective of religion and ethnic group. The person has the right to his or her own views and feelings. Even when you do not agree you should not judge the person negatively.

3. Be respectful

Be aware of the person's age, gender and culture. You should make them feel respected. Use words that are easily understandable, and that the individual does not find distressing or offensive, to refer to schizophrenia or mental illness.

4. Be patient

The individual and the family may take a lot of time to understand information or to change their behaviour.

5. Encourage trust

Respond sensitively if the individual or family tell you private or distressing information (for example, regarding sexual violence), and reassure them that you will not discuss this with other members of the community.

10.3 What skills can you use when communicating?

1. Show the person that you are listening to them

Listen carefully to what the person with schizophrenia is saying. If you show the person that you are listening they are more likely to feel comfortable to say how they feel. You can do this by:

- Keeping appropriate eye contact with the person
- Use body language. Make encouraging facial expressions and gestures. Sit facing the person with your arms unfolded.
- Don't keep checking your watch or phone.
- Keep your phone on silent mode. Don't answer it unless it is your supervisor. Don't read or respond to text messages.
- Keep your voice gentle. Speak slowly and clearly.
- Don't interrupt the person or ignore what they are saying

2. Use questioning skills

'Open ended' questions can be useful in getting the person to talk. They are questions that cannot be answered in a few words or sentences. For example, *"Could you tell me more about that?"* and *"How did you feel when that happened?"* Try not to put your own view into the question. For example, "Don't you think it would be helpful if you got back to work?" This may sounds judgemental and threatening. Instead you could say, *"What do you think would be helpful for you?"*

3. Be observant

You should be observant of the body language that the individual uses. For example, if the person has a smile on her face but has her fists clenched or twisting her fingers vigorously this may indicate a build-up of tension.

4. Use encouragement

Use word to encourage the person to carry on talking, for example "um", "really" or "ah ha". Nod your head and smile at the person. Sometimes just the repetition of a word leads to the person giving further details. If the person says "My life is a mess" you could reply *"A mess?".* This encourages the person to say what she means. You can also repeat back the person's story in your own words. This is useful to make sure you have understood correctly, and to encourage the person to talk about the problem in detail.

5. Notice the feelings of the person

It can be useful to notice the feelings of the person and show them what you have noticed. This helps them to feel understood. You might notice feelings through what the person says or how they act. For example, if the person says "I was so angry that I felt like hitting him", you could reply "You *must have been really angry".* Or if the person is biting her lips, you could say "*You seem very worried today*".

6. Notice the positive things

Noticing the positive things and how the individual is improving will help to improve their self-esteem. For example *"You say you are unhappy about your daughter-in law being rude to you... but it is good to hear that you have been enjoying time playing with your grandson."* However, try not to be unrealistic or to give false hope for things that definitely won't happen.

10.4 What approaches are not helpful when working with families?

- Telling the person what to think or do or making decisions for them, without asking them about their experiences or ideas
- Making judgements on whether the person is 'good' or 'bad'
- Blaming the person if they have difficulty changing their behaviour
- Making promises that you cannot keep
- Pretending to understand what they mean when you don't
- Not responding when the person tells you something sad or difficult
- Telling the person about your personal experiences
- Using offensive words for schizophrenia or mental illness

10.5 Problem solving

Sometimes people with schizophrenia and their caregivers find their problems overwhelming. Worrying or thinking too much about their problems can make them feel stress (see Chapter 23). This stress may even make the illness worse or cause a relapse (see Section 2.4). You can use **problem solving** to help individuals find ways around their problems. You can use this method for different types of problems, for example dealing with stigma and discrimination (see Chapter 28) or planning how to get back to farm work (see Chapter 27). People with schizophrenia may have many worries or problems. Try to focus on one at a time. Pick the one that relates to the module you are covering, or the one which is most important to the individual at that time. Once you have decided which problem you are going to focus on, follow these steps:

1. Describe the problem

- Discuss together what the problem is
- Try to describe it in the clearest way

2. Think of possible solutions

- Together, list as many solutions as possible to solving the problem. Sometimes you may need your supervisor's help to think about possible solutions.
- Try not to think about whether these are good or bad suggestions

3. Discuss the possible solutions

- Discuss each possible solution in turn
- For each one, now decide together whether it is a good suggestion or not useful
- For each of the good suggestions, discuss together the good and bad things about it.

4. Decide on a solution

- Decide which of the possible solutions is the best. Always check the individual is happy with this solution.
- Discuss the step by step actions which are needed to carry out the solution.
- Think about who will be involved and what their roles will be

5. Carry out the solution

- Support the individual and family to carry out the solution as you have discussed
- Check on their progress at later sessions

10.6 Confidentiality

Information that you gather during your work must be kept secret from other people, even from the patient's relatives, unless:

- The patient gives permission for you to discuss it with them
- It is essential that you tell other people in order to protect the person. For example, if you are worried the person is at risk of suicide (see Chapter 14).

The places where you talk to people with schizophrenia and their caregivers should be arranged in such a way that no-one can listen to your private discussions. Explain that you won't tell other people about the things that they tell you unless it is necessary for helping them. Remember to ask permission of the individual and caregiver before discussing an individual with a community leader or community member. This will help in building trust between you and the individuals you work with.

10.7 Summary

- Good communication between you and the individual and family is essential for CBR
- When working with families you should be: understanding, non-judgemental, respectful, patient and encourage trust
- The skills you can use include: listening and questioning skills, being observant, using encouragement, noticing feelings and noticing positive things.
- You can use Problem Solving to help individuals find practical ways around their problems

11 Creating a trusting relationship

11.1 What is a trusting relationship?

A trusting relationship means that there is a bond between you, the individual and family. It means the individual and family speak freely and share their ideas and concerns.

11.2 Why is it important to have a trusting relationship?

It is very important to have a trusting relationship with the individual and family. This will help the CBR in the following ways:

- The individual and family members are more likely to tell you the information you need to help them. For example, worries about money or family conflict.
- They are more likely to listen to the information you give them
- They are more likely to try the activities which you suggest
- They are more likely to take their medication regularly
- They are more likely to keep returning to the health centre
- They have a way of expressing their emotions
- They are more likely to be hopeful that things can improve

11.3 How to form a trusting relationship

1. Introduce yourself

When you first meet the individual and family, introduce yourself. Introduce yourself to any new family members you meet at later sessions.

2. Learn about the family

Try to learn as much as possible about the individual and their family before you meet them. Find out from the health centre staff or Health Extension Worker how long the individual has been unwell and what problems they have had. When you meet the family, try to understand who is involved in the care of the individual and who has power and influence. You may need to work with this family member/s to change how the family treats the individual.

3. Focus on the problems that are important to the individual

Listen to what the individual wants to change and help them to achieve it (see Chapter 13).

4. Keep each session focused on the individual

Near the beginning of each session, ask the individual “What would make this a helpful visit?” or “What would you like to see different after our meeting?” It is not usually helpful to refer to your own experiences.

5. Get detailed information from the individual

Try to get as much information as possible about the current situation, and any difficulties they have. For example, an individual may be drinking a lot of alcohol. On asking him for the reasons for drinking, he may say this is due to frequent quarrels at home. You can then discuss how to reduce the quarrels, instead of drinking to deal with the problem.

6. Listen to ideas, beliefs and concerns

Listen to the ideas and beliefs of the individual without judging them or telling them they are wrong. For example, the individual may find it helpful to make regular visits to holy water. If this is not causing harm you should support them to continue, even if you do not believe that holy water can cure the illness. You should listen to their concerns. For example, even if they have worries about side effects that you think aren’t important, you should not simply dismiss them.

7. Treat the individual with dignity and respect

Make the individual the centre of your attention at all times. Treat them with the respect you would with any other person, during the sessions and if you meet them in the kebele. Don’t use stigmatizing words like ‘mental’. Use non-stigmatizing words like problem and illness. These actions will help to improve the self-esteem of the individual. They also help to reduce stigma and discrimination from the family and community towards the individual.

8. Stay neutral during family discussions

There may be disagreements within the family on the best way to do things. Try to stay neutral.

9. Keep clear boundaries

You are aiming for a close working relationship with the individual and family. However, it is not appropriate for them to intrude on your personal life. The family should not visit you at home. However, it is fine to greet the family if you see them outside their home. It is fine for the family

to give you tea or coffee when you visit, but it is not normally allowed for you to receive gifts. You should also not pay for the individual's food or medication yourself.

11.4 Challenges with forming a trusting relationship

Due to their illness people with schizophrenia sometimes find it more difficult to form a relationship or bond with other people. You should always keep trying. But this is part of the reason why it is important to also make a good relationship with the family.

11.5 Summary

- Creating a trusting relationship between you, the individual and the family makes it more likely that CBR will be helpful.
- You can form a trusting relationship by: finding out about the family, keeping the sessions focused on the individual and treating them with dignity and respect, listening to beliefs and concerns, and staying neutral during family discussions.

12 Needs Assessment

12.1 What is the Needs Assessment?

People with schizophrenia have many different sorts of problems. Some problems will be more important for particular individuals and families. Many of these problems can be helped through CBR. The Needs Assessment is an organised way of finding out what problems each individual and family are facing.

12.2 Why do we do the needs assessment?

- The Needs Assessment tells us what problems the individual and family have, and which are the most important problems at that time. This helps us decide what improvements the person with schizophrenia and their family would like to work towards first. This is called Goal Setting (see Chapter 13). This helps us to decide which Modules of CBR to start with, and which to leave until later.
- Doing the Needs Assessment will help the individual and family understand that you are interested in their problems. This will help build a trusting relationship (see Chapter 11).
- It is also useful to look back at the Needs Assessment at the end each Phase. Then we can see whether the problems that the family had at the beginning are still there, and what still needs to be done.

12.3 Who is involved in the needs assessment?

The Needs Assessment should always involve you, , the individual and the primary caregiver. Your supervisor should be involved at the beginning of Phase I and may be involved in Phase II and III. You may include other members of the family who are involved in the individual's care. Around the time of the Needs Assessment, you should accompany the individual to the health centre for a review by the nurse or health officer. By observing what the nurse or health officer says you will get extra information about the needs of the individual. There may be different views about what the most important needs are. For example, the individual may feel the most urgent need is to get back to farm work. The family may feel that the symptoms need to be under better control first. At this stage it is important to get all the points of view. In Chapter 13 you will learn how to set goals for the individual together with the family.

12.4 When is the needs assessment done?

The Needs Assessment is done three times during CBR, at CBR Reviews I, II and III.

12.5 How do we do the needs assessment?

You will use a detailed checklist to assess whether there are problems in the areas where people with schizophrenia usually have difficulties.

Using the Needs Assessment form

The Needs Assessment Form (Form 4) covers the following areas:

- Understanding schizophrenia
- Accessing health services
- Preparing for a crisis
- Human rights issues
- Symptoms
- Taking medication
- Social life
- Family life
- Daily functioning
- Work
- Empowerment
- Literacy

You should go through each potential need in turn and ask the individual and family whether they have any problems in this area. For example to assess social life you could say, *"Sometimes people with schizophrenia have problems doing the activities in the community that they used to do, or that other people their age do. For example, being part of an Edir group, going to kebele meetings or being part of a mahaber group. Have you had any problems with doing these sorts of activities?"*

Using observations

You and the supervisor can also use your own observations about what the problems are. For example, you may notice that the individual is very drowsy (which might be a side effect of medication), or that a family member often shouts at the individual.

Recording the needs assessment

Mark on the assessment form whether each possible need is:

- Not a problem for this person (everyone agrees there are no problems). For example, an individual remembers to take their medication every day without being reminded.
- A partially met need (there are a few small problems). For example, an individual occasionally does not go to church, even though they would like to.
- An unmet need (there are problems). For example the individual does not do any of the farm work that they used to do.

For each need write some comments about what you have been told and what you have observed. Include:

- Detailed information on the need. For example, what type of work the individual would like to do or what self-care tasks are most difficult.
- How long this has been a problem. For example, the individual may have had difficulty socialising for a long time, but there may be new medication side effects.
- How important this need is for the individual. This will help later when goal setting.
- If this is the second Needs Assessment, record what progress has been made since the previous needs assessment.

Assessing personal needs

In addition to the checklist you should also ask the individual and family to tell you about any other problems or needs that have not been covered. These are known as 'personal needs' and you should record them on the needs assessment form.

> **Yosef's story**
>
> *At Review II, Berhan does the Needs Assessment with Yosef and his family. Haile, her supervisor, is also there. She records everything on the Needs Assessment Form.*

12.6 Tips for completing the needs assessment

- Some individuals and family members may start by saying that they have no problems. It is important to gently continue asking for difficulties. You may need to come back to sections on the checklist. Sometimes you will need more than one home visit to complete the needs assessment.
- When you ask each question, think about what is likely to be usual for that individual. For example, if young women do not usually attend the kebele meetings, then it may not be appropriate to ask about problems with this.

- It is likely that the individual and family will ask you questions about the illness whilst you are doing the needs assessment. You should be ready to answer some questions, but remind them there will be a chance to discuss these things in more detail later.

12.7 What do we do with the information from the needs assessment?

In Phase I, we do the Needs Assessment to help to get to know the individual and the family. In Phases II and III we use the information for Goal Setting (see Chapter 13).

12.8 Summary

- We do a Needs Assessment to understand what problems the individual has and to decide which parts of CBR to start with
- We conduct the needs assessment by asking questions to the individual and family, making observations, and attending the health centre with the individual
- On the Needs Assessment Form you should record whether each possible need is not a problem for this person, an unmet or partially met need.

13 Goal Setting

13.1 What is Goal Setting?

Goal setting is about deciding together which needs to address and in which order. It should be a positive process, not one that makes the individual feel helpless or a failure.

13.2 Why do we need to do Goal Setting?

Goal setting is important so that the individual, family, you and your supervisor all know what you are working towards. This can help individuals to recover more quickly. Goal setting makes sure that the CBR Modules that you do with each individual are the right ones for him or her. Goal setting also gives a sense of achievement and progress when a goal is achieved.

13.3 Who is involved in Goal Setting?

Goal setting should always involve you, the individual and the primary caregiver. You may involve other members of the family who are involved in the individual's care. Your supervisor should be involved at the beginning of Phase I and may be involved in Phase II and III.

13.4 When do we do Goal Setting?

We do Goal Setting as part of the CBR Reviews at the beginning of each Phase. Goal Setting should always come after the Needs Assessment.

13.5 How do we do Goal Setting?

Phase I

Phase I is about making sure all individuals have their basic needs met. Even though you might have found out about lots of different problems on the Needs Assessment, during Phase I you should focus on basic needs. The potential goals for Phase I are therefore :

- Individual and caregiver have been informed of what schizophrenia is, available treatments, and the potential for recovery
- Individual is able to access medication and attend health centre for mental health as indicated by clinical status
- Crisis management plan is in place
- Individual is not chained or restrained

Not all individuals will have problems with these basic needs, so not everyone will need to set a goal around all of them. The order in which you address the Phase I goals will also depend on

the situation of the individual. For example if an individual is currently chained, you should usually address the goal ‘Person with schizophrenia is not chained or restrained’ first, before going on to the other goals. You should work together with the individual, family and your supervisor to decide the best order to address the goals. On the Phase I Goal Setting form (Form 5), you should make notes about the most important goals to address first. Add a timeframe for how long you expect it to take to achieve the goal. In addition to these core goals you should also ask the individual if they would like to set a personal goal. You should fill in the Rehabilitation Plan (Form 8) to summarise the information from the Initial Assessment and Goal Setting. This form should be filled in using simple language that the family can understand. Make a copy of the form for the individual and family to keep.

Yosef’s story

At Review I, Berhan does Goal Setting with Yosef and his family. Haile, her supervisor, is also there. She records everything on the Goal Setting Form.

Phase II

In Phase II, all the goals are based on the particular needs of the individual and family. This means they will differ from individual to individual. To set the Phase II goals you should go through the list of needs that you marked as ‘Partially met’ or ‘Unmet met’ on the Needs Assessment you complete as part of CBR Review II. The number of goals for Phase II will vary between individuals but should not be more than five or six. The decision about which goals to focus on should be made together with the individual, family, you and your supervisor. You should also decide which goals to focus on first, and how long you expect it will take to achieve each goal. You can continue working on Phase I goals if you feel ongoing work is needed to maintain the situation. You should encourage discussion about the following:

- Which goals are most important to the individual and why?
- Which goals are most important to the family why?
- Which goals need to be achieved first before other goals can be achieved? For example, the individual may have problems remembering to take their medication. As they still have many symptoms this may be the reason why they also have difficulties with self-care and social life. You may agree that it is important for the person to start taking their medication regularly, before moving on to addressing self-care.
- Which goals are realistic?

On the Phase II Goal Setting form (Form6), you should tick off the goals you have chosen, and make notes about the most important goals to address first. Include information on why the family wants to focus on those goals first. You should add details on what you hope to achieve. For example, if the goal is 'Individual has good self-care' you could add the particular tasks you are trying to improve, such as getting washed. At any time you may be working towards several goals at once. You should also discuss the personal goal set in Phase I, and either continue working towards this or set a new personal goal. Record any personal goals on the form. Once again, you should summarise the information from goal setting by updating the Rehabilitation Plan (Form 8). Make a copy of the form for the individual and family to keep.

Yosef's story

At Review II, Berhan does Goal Setting with Yosef and his family. Haile, her supervisor, is also there. She records everything on the Goal Setting Form.

<u>Phase III</u>

Setting and prioritising goals in Phase III should follow the same process as Phase II. However, in Phase III you should focus on maintaining the improvements made in Phase III, rather than starting to work on lots of new areas. The number of goals for Phase III will vary between individuals but should not be more than three. Unless they have already achieved the goal in Phase II, all individuals should have the following goal:

- Individual has relapse prevention plan in place

On the Phase III Goal Setting form (Form 6), you should tick off the goals you have chosen, and make notes about the most important goals to address first. Summarise the information on the Rehabilitation Plan (Form 8).

13.6 What do we do with the information from Goal Setting?

Each goal has a specific Module connected to it. If an individual is working towards a goal, then you should complete the connected Module at some point during the Phase. More than one goal may be addressed by the same module, so you can try to work on these linked goals around the same time. Even if you are only trying to achieve one goal linked to a module, you should normally complete the whole module. However, you should tailor the module to help with the specific problems that the individual faces. Remember the focus is on supporting individuals to achieve the goals, not just to complete the module as fast as possible.

Phase I

In Phase I everyone should complete the same Modules, even if they haven't set a specific goal relating to the module. These modules are:

- Understanding schizophrenia and its treatment
- Improving access to health services
- Addressing human rights issues
- Preparing for a crisis

However the order of the Modules should be decided depending on the situation of the individual. The Initial Assessment will help you to decide this. How long you spend on each Module will also depend on the individual. For example, if an individual has a good understanding of schizophrenia, is not chained or tied up, and regularly access health services, they may only need one or two home visits to complete all these Modules. Or you may need to spend many more home visits with someone who does not access health services and who is currently suffering severe symptoms.

Phases II and III

Once you have decided which goals to focus on in Phases II or III, and in which order, you should then note and discuss which modules are needed to achieve these goals. This should be easy, as each goal has a recommended module linked to it. These are listed on the Phase II and III Goal Setting forms. On the forms you can also write other actions that you think might help to achieve the goal, but which aren't in the module. As in Phase I the length of time to complete each Module will vary between individuals.

13.7 How do we know if goals are achieved?

You should always be thinking about which goals the individual is working on at the moment. At each home visit you will check and record the progress of each of the current goals using the Home Visit Form (Form 11). On this form for each goal you record:

- The module/s you are currently working on which relate to that goal
- Any community engagement work or other actions underway which relate to that goal
- Any issues or problems relating to the goal
- Whether the goal is achieved, partially achieved or not achieved by the end of the home visit.

In Phases II and III a goal is normally only achieved when there is a change in the behaviour, actions or experiences of the individual or family. You should ask the individual and family whether they feel the goal has been achieved. You will also meet with your supervisor every two weeks to discuss the progress of each individual (see Chapter 34). At these meetings you should discuss whether each of the goals you are working on has been achieved yet. There is not a fixed time when any of the goals should be achieved by. In your discussions with the family you should focus on the positive things. Try not to make the individual feel they have failed if it takes a long time to achieve the goal. Once a goal is achieved tick it off on the Goal Setting Form.

Yosef's story
At each visit Berhan fills in a home visit form, which keeps track of the goals Yosef is working towards.

13.8 When do we move to the next phase?

Moving from Phase I to Phase II

Once all the Phase I goals have been achieved, you should arrange CBR Review II with the individual, family, you and your supervisor. If the individual is not ready for CBR Review II by about 2 months, you and your supervisor will review the situation together. There may be one or more goal that you, your supervisor, the individual and family agree may never be achieved despite the best efforts of you all. If this happens, with the permission of your supervisor, the individual can move onto Phase II without having achieved that goal. You should however still continue to support the individual to achieve the goal during Phase II. It is not necessary to have achieved the personal goal before moving onto Phase II. Your supervisor will be monitoring the progress of each individual.

Moving from Phase II to Phase III

Once all the Phase II goals have been achieved, you should arrange CBR Review III with the individual, family, you and your supervisor. If the individual is not ready to move to Phase III by about 8 months, you and your supervisor will review the situation together. If there is a goal that you agree may never be achieved, you can move onto Phase III without having achieved that goal.

13.9 Tips for Goal Setting

- Remember to choose realistic goals with the individual and family. For example, if the individual is still unwell at the beginning of Phase II, it may be better to choose ‘Taking medication’ instead of ‘Getting back to work’ as a goal at this stage.
- Remind the individual that just because a goal has not been chosen at this stage does not mean it will never be addressed. You can add goals later, for example in Phase III.
- If a particular goal has not been achieved yet, keep focusing on the progress made.
- Remember that if you, your supervisor, the individual and family agree that a goal may never be achieved, it is acceptable to move onto the next Phase anyway.

13.10 Summary

- We do Goal Setting to decide which needs to address and in which order, and to follow the progress of the individual
- In Phase I the Goals are the same for everyone. You should decide the order to address them by discussing with the family and recording on the Phase I Goal Setting Form

  In Phases II and III the Goals are different for each individual. You should choose no more than five or six goals to focus on in each Phase and record them on the Phase II or III Goal Setting Form
- The goals you choose will tell you which Modules to deliver

14 Risk assessment

14.1 What risks do people with schizophrenia face?

Suicide

People with schizophrenia are about ten times more likely to commit suicide (kill themselves) than other people. Suicide is therefore quite a common cause of death in people with schizophrenia. The most common ways people commit suicide in rural Ethiopia are hanging, drowning and swallowing pesticides or chemicals.

Attempted suicide

Attempted suicide is where someone tries to kill themselves but does not succeed. This is a serious warning sign that the person needs urgent help. Suicide attempts are much more common than suicide, where people succeed in killing themselves. Young women are most likely to attempt suicide in Ethiopia. People who have attempted suicide are more likely to commit suicide later.

Chaining and tying up

People with schizophrenia are quite commonly tied up or chained up from time to time. This may take place at the family home, or sometimes at a holy water site or traditional healer's home. The person may be tied up for a period of days to weeks, or sometimes months to years. See Chapter 7.

Physical abuse

People with schizophrenia may be beaten by family members. Sometimes they are beaten by holy water priests or attendants or traditional healers as part of the treatment. See Chapter 7.

Emotional abuse

People with schizophrenia may be treated badly even without being physically abused. For example, they may be shouted at or treated in an undignified way. This may take place within the home or outside.

Sexual violence

People with schizophrenia are particularly vulnerable to sexual violence, for example rape. This may happen within or outside the family home.

Neglect

Sometimes people with schizophrenia are not given enough food, or don't wear any clothes. Sometimes they are homeless and have no shelter. See Chapter 7.

Risks to children

Children who live in a household where a person has schizophrenia may be vulnerable to neglect or abuse. This may happen because a parent (either with the illness or a caregiver) is unable to look after the child properly due to burden on the family due to the illness.

Environmental risks

Sometimes people with schizophrenia are not able to look after themselves as well as normal. This can result in them being hit by cars, falling off buildings or being attacked by wild animals.

Imprisonment

Some people with schizophrenia get involved with the police, particularly if they are behaving in a disruptive way, and may end up imprisoned in a jail.

14.2 Why do we need to assess risk in people with schizophrenia?

Once we know the risks individuals are facing we can take action to try to reduce the risk. In some cases we may save an individual's life. In other cases, we may improve their situation so they have a better quality of life and find it easier to get back to usual activities. It is not true that asking about suicidal ideas introduces the idea in the person's mind. Instead, asking whether the person has experienced suicidal ideas is the only way to identify risk and provide the necessary help to prevent suicide.

14.3 How do we assess risk in people with schizophrenia?

In your work with individuals and families you may become aware that the individual is at risk in different ways, just by listening to them and observing their situation. Below you will find some specific things that will help you know that the individual is at risk. In addition, at every home visit you should sensitively ask questions to check for suicide risk and chaining or restraint. At each CBR Review your supervisor will do a full risk assessment by asking questions to the individual and family. You should also be present and contribute where you can. It may feel very sensitive to ask about these topics. You can start by saying, "I need to ask you some questions which might be difficult or sensitive. Before we start I want to let you know that these are routine question that we ask to everyone."

Suicide

You should ask some questions to assess suicide risk at every home visit. One way to start asking

about this is: "*How do you feel about life at the moment?"* If the individual does not say anything very negative, you can carry on with the home visit as usual. If the individual says that they are feeling very negative, or things are going very badly, you should ask some more questions, such as *"I can see you are going through a very difficult period. In your situation, some people might feel that it is not worth carrying on with life. Have you ever felt like that since I last saw you?"*

Or you can use other ways of asking the question, for example,

"Have you felt it would be better if you did not wake up in the morning?"

"Have you felt it is not worth carrying on with life?"

"Have you felt that your problems in life are too difficult to be solved and that you would be better off dead?"

If the individual says yes to any of these questions you should ask them, *"Have you made any plans for harming yourself?"* This will help you know how serious the risk is.

The following things may also indicate the individual is at risk of suicide:

- Any reference to death or dying, in verbal or written expression or a strong focus on an individual who committed suicide
- Not being willing to discuss the topic and seeming distressed
- Any statements that "life is not worth living"
- Impulsive and potentially dangerous behaviour. For example drinking alcohol or using khat.

If the individual tells you they have thought about suicide, listen carefully and respond sensitively using your communication skills (Chapter 10).

Chaining

You should assess for chaining at every home visit. It may be obvious that the individual is chained or you may notice wounds or sores on the individual. If it is not obvious you can check by asking: "Since I last saw have you been chained or restrained at home, at a holy water site, or by a traditional healer or by anyone else?"

Physical, emotional or sexual abuse inside or outside the home

You may notice signs of physical injury such as bruises or cuts. You may notice that a family member treats the individual in an abusive way.

Neglect and not eating or drinking

You may notice the individual looks very thin or malnourished, or that they are not wearing any clothes.

14.4 How do we respond when we find there are risks?

Record any risks you find on the Home Visit Form. Follow these steps.

1. Find out if there were any particular circumstances or factors leading to the risk.
2. Discuss with your supervisor, the family and individual immediate actions that would be helpful.
3. Follow the flow chart (Chapter 35) for the situation. It will tell you the specific steps you need to take. The steps for suicide risk and chaining are also given below (section 14.5).
4. Discuss with your supervisor, the family and individual steps to reduce the risk in future.
5. Follow up at every visit to check these steps have been taken, and if there have been any further problems.

14.5 How do we deal with suicide risk?

In addition to the general steps (Section 14.4), if you identify a risk of suicide you should always follow these steps:

- Check the individual is taking their medication and if not encourage them to do so
- Facilitate access to the health centre as soon as possible for a review. Fill in a Health Centre Referral Form (Form 14).
- Inform your supervisor immediately, if they are not already aware.
- If the individual does not attended the health centre after one day, make a joint home visit with your supervisor.
- At this home visit you and your supervisor should discuss with the family and the individual (if possible) the reasons why they have not attended.
- Try to address these reasons, for example:
    - Reiterate the importance of receiving treatment for improving symptoms
    - Mobilise community leaders to help the individual travel to the health centre
    - Also see Chapter 17 Accessing Health Services
- Your supervisor will do a more in-depth assessment of suicide risk
- Your supervisor will then give discuss the risk assessment with the Psychiatric Nurse

- If the individual still has not attended the health centre within 2 days, and the Trial Psychiatric Nurse thinks the individual is at high risk they will then do a home visit to review the situation and improve the treatment.

14.6 How do we deal with risk of chaining or restraint?

In addition to the general steps (14.4), if you identify an individual who is chained you should follow these steps (see Chapter 19 for more detail):

- Tell your supervisor immediately.
- Check the individual is taking their medication and if not encourage them to do so
- Facilitate immediate access to the health centre for a clinical review. Fill in a Health Centre Referral Form (Form U).
- If the individual is still chained and they have not attended the health centre after one week, make a joint home visit with your supervisor.
- At this home visit you and your supervisor should discuss with the family and the individual (if possible) the reasons why they have not attended.
- Try to address these reasons
- If the individual is still chained and they have not attended the health centre after one week, you should inform the Trial Psychiatric Nurse. They may do a home visit to review the situation and improve the treatment.

14.7 Summary

- We assess risks to find out what risks an individual faces, so we can take action to protect them
- Your supervisor will do a full risk assessment at every CBR Review. You should ask sensitive questions about suicide risk and chaining at every home visit
- When a risk is identified there are certain steps you should follow. Usually this is referring to the health centre and informing your supervisor, if they are not aware yet.

15 Being aware of the caregiver's needs

15.1 What problems do caregivers have?

As we learnt in Chapter 5, when someone has schizophrenia this can put a burden on the whole family. Often the burden falls on one person in particular. This may be the spouse, parent or child. It is often, but not always, a female caregiver who takes most of the responsibility. Female caregivers may be less likely to get support from other family members or people in the community. Sometime the stress on this person is so great that they become unwell themselves. The caregiver may develop distress, depression or anxiety. Some of the signs of this are when the caregiver:

- Complains of many physical symptoms but no physical cause has been found (e.g. headache, burning sensations, aches and pains)
- Has low energy; is always tired; has sleep problems or does not want to eat
- Always seems sad or anxious or irritable; feels hopeless or helpless or guilty
- Has low interest or pleasure in activities that used to be enjoyable
- Worries or thinks about day-to-day problems too much
- Is not able or motivated to do their usual job, housework or social activities
- Has been thinking of harming themselves, e.g. ending their life

15.2 Why is it important to think about the caregivers needs?

Caregivers have the right to be healthy, just like people with schizophrenia. Also, if the caregiver becomes unwell they will not be able to look after the person with schizophrenia very well.

15.3 How should we stay aware of the caregiver's needs?

Unlike for the person with schizophrenia, you do not need to do a full needs assessment with the caregiver. However, at every home visit you should think about how the caregiver seems to be getting on. Caregivers who are female, elderly, isolated or suffering from a physical illness are more likely to suffer from the stress of caregiving. Look out for any of the signs that they are distressed or depressed (section 15.1). If you notice any of these signs take these steps:

- If possible, try to talk to the caregiver by themselves, without the individual present. If you do this, you may need to explain to the individual that you are not talking about them behind their back. Explain that you just want to check how the caregiver is doing.

- Ask the caregiver how they feel they are coping and listen to their experiences. Ask if they have anybody they can turn to for support, for example a relative or neighbour.
- Tell them it is sometimes useful to discuss their problems with somebody else. Just by doing this, things can feel easier.
- If they are not already doing so, encourage them to join the Family Support Group (Chapter 32)
- If possible, involve the other members of the family. Speak to them and share your concerns about the stress on the caregiver. Discuss how they can take some of the responsibilities
- If you are very worried about the caregiver, you should suggest that they go to the health centre for a review with the nurse or health officer. You should also tell your supervisor.

Yosef's story

Berhan notices that Addis sometimes seems sad and distressed. She often sounds hopeless about Yosef's situation. Addis agrees to talk to Berhan alone. They both reassure Yosef that they are not talking about him behind his back. Addis tells her problems to Berhan. She admits she often feels unhappy and stressed. Addis agrees to share her problems with her cousin, who she trusts. Addis is also interested in joining the Family Support Group when it gets set up.

15.4 Summary

- Caregivers can become unwell through the stress of looking after a person with schizophrenia. It is often female caregivers who take on more responsibility.
- They may have physical symptoms, low energy, be very worried, or consider harming themselves
- Ask the caregiver how they are coping. If you are concerned try to ensure they get more support, for example from friends and family.

16 Module: Understanding schizophrenia and its treatment

16.1 What problems do people with schizophrenia and their families have with understanding schizophrenia?

Many people with schizophrenia and their families do not know very much about the illness. Some common beliefs, which are not true, include:

- No one with schizophrenia will improve or get well
- Medication can completely cure the illness
- Schizophrenia is caused by spirit possession or evil eye

Most of these incorrect beliefs are not harmful by themselves. However, some can be unhelpful. For example, if the individual and family believe the illness will never improve, they may make less effort to support the person. On the other hand, if they believe the medication will completely cure the illness, they may be disappointed when it takes a while to see improvements. The individual may then stop taking the medication altogether.

16.2 Why do people with schizophrenia and their families have problems with understanding schizophrenia?

People with schizophrenia and their families may have little understanding of schizophrenia because there was no information available to them. Even if they have been to the health centre, the nurse or health officer may not have had time to give the information properly.

People may find traditional beliefs make sense and that traditional practices are helpful.

16.3 Why is it important to give information about schizophrenia?

People with schizophrenia and their families have a right to have information about their illness. However, this does not mean we should force them to believe all of the information they are given. People can have lots of different beliefs at once. Having a certain belief, such as that the illness is caused by the devil, does not always stop people taking certain actions, for example taking medication.

If people have more information about schizophrenia, they may feel more positive about their illness and that things may improve. With this attitude, the individual and family may be more likely to take active steps to help recovery, including participating in CBR more fully. The more active steps individuals and families make, the more likely they are to get back to their usual activities.

16.4 How do we give information about schizophrenia?

1. Use these principles for giving information

Giving information about schizophrenia is an ongoing process. You will need to remind the individual and family about information you have already given them. Also, the issues the person faces, and therefore the information they need, will change as time goes on. It is likely that you will involve other family members apart from the individual and the primary caregiver when you give information about schizophrenia. Who you involve should be decided between you, the individual and the caregiver. Try to include those family members that have power and influence within the family, along with those who are helping to look after the individual. Here are some general tips on giving information about schizophrenia:

- Give the individual and family time to **ask questions** and pause after each section
- **Encourage** the family to tell you if they disagree with the information
- Be prepared to **discuss and clarify** the information with the family.
- Avoid **over-loading** the family with too much information or medical terms.
- Use **examples** from what you know about the individual
- **Listen** and understand the family's view of the illness.
- Be **sensitive** to any distressing experiences of the family members
- Tell the family if you do not know the answers to their questions. Before the next session, make sure you find out the answer by talking to your supervisor or looking up the information in this manual.

2. Explain why you are doing this module

Tell them why would like to give them information about schizophrenia

3. Assess understanding about schizophrenia

- To begin with, ask whether they think something is wrong with the individual or not.
- If so, ask them what do they think that is wrong with him/her? They are unlikely to report problems with "hallucinations" or "delusions". They may instead report the effect of hallucinations or delusions on the individual's behavior. For example, that he or she laughs and talks to himself, says things that do not make sense, is not doing well in work or school, or does not want to spend time with other people.
- Ask if they know what illness the individual has. They may not have been told the

diagnosis, or if they have, they may not understand the details.

- Find out about their knowledge about medications. Do they know why medicines are given? What is the relationship between the medications and the symptoms? What are the side effects of the medication?
- Ask if they think medication can make them better or worse.
- Ask what they think about how long the illness lasts. Some families expect the illness to be cured with medication. They may not be aware that there may be many episodes.
- Finally, ask what they think about the causes of the illness. Most family members will not consider mind problems as being the main cause. They may wonder if they are being punished for something they did wrong, and therefore feel guilty, angry or responsible. Some family members may think that "evil spirits" cause it. Parents may wonder if it has been inherited and may blame the other spouse if they have a relative with a mental illness.

4. Give information about typical symptoms of schizophrenia

- Explain each symptom group in turn (hallucinations and delusions, problems with motivation, problems with thinking clearly and not understanding they are unwell)
- Use examples from the individual's behaviour to help explain
- Tell the family that each individual has a different symptom pattern and will have different symptoms at different times. This is important, as family members may not listen to you when you describe symptoms that the individual does not have.
- Emphasize that the hallucinations and delusions cannot be easily understood. They are thoughts, ideas, and voices that go in inside the individual's head. The family can only see their effects through the individual's behavior.
- Emphasize that the symptoms are real to the individual and they cannot control them. For example, they cannot help but answer the voices they hear.
- Emphasize that problems of motivation and problems with thinking clearly cannot be easily overcome by the individual.

5. The causes of schizophrenia

- Explain that both stress and vulnerability are usually needed for a person to develop schizophrenia, but sometimes we don't know why a person develops the illness. Give

examples of stress and vulnerability. When discussing vulnerability, do not emphasise that having a close family member with schizophrenia increases the risk, as this may be stigmatising. Instead, focus on difficult early life experiences.

- Families usually have their own beliefs about the cause of the illness. You should listen to these ideas. It is possible to have more than one belief about the cause.
- Family members may think they caused or could have prevented the illness in some way. These ideas make the family feel guilty and depressed. You should reassure the family that they did not cause the illness.
- If the family asks you, you can tell them that their children will most likely NOT develop the illness too.

6. Course of schizophrenia

- Inform them that there are good chances individual can recover from an episode and have a near normal life.
- Also tell them that the illness may come back (relapse), even once they have started to feel better. Describe some of the signs that a relapse is coming (e.g. problems with sleep, feeling angry or anxious). To try and stop this happening they should continue medication all the time. However sometimes things out of the individual's control will happen e.g. physical illness or life stress, which may trigger the illness to come back. If the illness does come back it is important not to give up hope.

7. How common is schizophrenia

- Tell them about 10 people have schizophrenia in an average kebele. You don't need to give them the exact number for the kebele they live in.

8. Medication

- **What are the medicines for**

Tell them medication may reduce symptoms, prevent relapse and improve the chances of recovery.

- **How do they work**

Tell them the medication works by improving the way the mind works and prevents it from getting sick again. The result of this will be fewer hallucinations and delusions.

- **What is the name of the medication that has been prescribed?**
- **What are the common side effects**
- **Regular medication**

Discuss with the family and emphasize the need for taking medication regularly. There is a separate module for support with taking medication (Chapter 20).

- **Continuing medication**

Emphasize that medication will need to be taken for a long period of one to two years or even forever. The duration of the treatment depends on how well the individual is recovering and not on how long the illness was there before the treatment started.

- **Address concerns**

Some families think that the medications are addictive if used for a long time. The family may then decide that the individual should learn to cope without the medication. This view is not correct. Be clear that the medicines are not addictive.

9. Stress-vulnerability model

- Explain that both reducing stress and taking medication can help reduce relapses and help the person to recover
- Explain that other parts of CBR can help reduce stress, for example improving the family environment, reducing stigma and discrimination

Yosef's story

Berhan explains what the symptoms of schizophrenia are, referring back to Yosef's problems. She explains the causes of schizophrenia and the importance of medication. Addis is still sure that Yosef's illness was caused by spirit possession, but she can also see that the medication is useful. Addis does not try to change Addis' beliefs about spirit possession. Yosef's family have lots of questions so it takes two visits to cover all the information.

Follow up

- Be prepared to remind the family of the information at later home visits
- Look up or ask your supervisor any questions that you did not know the answer to. Give the individual and family the information at the next home visit

16.5 Summary

- Many people with schizophrenia and their families do not know much about the illness
- If people with schizophrenia know more about their illness they are more likely to take positive steps towards recovery
- You should give the following information about schizophrenia: typical symptoms, causes, course, how common it is, medication (names, side effects, importance of taking regularly and continuing once well) and the stress-vulnerability model.

17 Module: Improving access to health services

17.1 What problems do people with schizophrenia have accessing health services?

There are three levels of health services that are available for people with schizophrenia.

1. Health centre

Nurses and health officers in Sodo woreda are trained to look after people with schizophrenia. Nearly all the medical care for individuals takes place in health centres

2. Butajira Outpatient Clinic

Psychiatric nurses at Butajira have expert knowledge about schizophrenia. Sometimes health centre staff will send individuals here when they are very unwell.

3. Ammanuel Hospital in Addis Ababa

Psychiatrists (doctors with expert knowledge on mental illness) look after people with schizophrenia at Ammanuel Hospital. Rarely, an individual will be sent here when they are very unwell.

Problems accessing health services

Sometimes people with schizophrenia have difficulty getting to the health centre or hospital to get the treatment they need. This may mean that even though the individual is willing to see the nurse or health officer and to take the medication, they are not able to.

17.2 Why do people with schizophrenia have problems accessing health services?

There are various reasons why people with schizophrenia have problems accessing health services. These include:

- The family cannot afford the medication
- The family cannot afford to travel to the health centre
- There is nobody to go with the individual
- It is difficult to take the individual to the health centre because they are unwell or it is far.
- The medication is not always available at the health centre
- The individual is chained up at home

In some cases the individual is able to access health services but chooses not to because they do not want to receive treatment.

17.3 Why do we need to improve access to health services?

Getting access to the health centre is essential for recovery from schizophrenia. At the health centre the individual is reviewed by a nurse or health officer who will:

- Check overall progress
- Prescribe the medication or give the injection. People with schizophrenia need to take their medication regularly in order to feel better and get back to usual activities (see Chapter 20)
- Check for and try to improve side effects
- Change the dose of the medication if it is not enough or too much
- Refer them to Butajira outpatient clinic if they cannot meet all individual's needs

How often people with schizophrenia need to attend the health centre depends on how bad their illness is at that time. Usually they need to see a nurse or health officer and collect their medication every 1-3 months.

17.4 How can we improve access to health services for people with schizophrenia?

1. Inform the family what health services are available

2. Explain why you are doing this module

Explain the importance of accessing health services.

3. Assess problems with accessing health services

Find out what the current arrangements for accessing medication. Then assess any reasons why the individual has problems accessing health services, or might do in the future. Use the potential reasons above (Section 17.2) as a starting point. If the individual is able to access services but does not want to, you may need to complete Module: Supporting Individuals to Take Medication (Chapter 20).

4. Use problem solving to improve access to health services

You should then discuss each reason or problem in turn with the individual and caregiver. Encourage them to think of ideas for how to get around the problem using the problem solving approach (Section 10.5). Discuss the approaches they have already tried. Build on their ideas and then suggest some of your own if everything has not been covered. Some or all of the following methods can be used.

a. Explore accessing free medication

A certificate for free medication can be given by the kebele administration to the 'poorest of the poor'. Only 5 in every 1000 families can receive this certificate, therefore not all people with schizophrenia will be eligible. However, if you think that the family is amongst the poorest in the kebele and they do not already have the certificate, you should take some action. First of all encourage the family to approach the kebele leaders and request this certificate for themselves. Discuss with the caregivers the kind of things they might say. If this is unsuccessful, you should approach the kebele leader yourself and request that the family receives the certificate. See Task 8 in the Community Engagement chapter.

b. Make travelling to the health centre easier

Discuss potential sources of help for travelling to the health centre. This may include extended family members, neighbours or community leaders. They may be able to give money for the transport costs, or help to accompany the individual, along with family members, on the journey. If the family is unable to find additional support themselves, you could meet with a community leader and ask for their help. See Tasks 8, 9 and 10 in the Community Engagement chapter. You are not normally expected to accompany the individual to the health centre yourself, except for around the time of the CBR Reviews.

c. Address financial problems

Poverty is a long-term problem, which may to be difficult to improve over the course of receiving CBR. However, financial problems may be addressed to some extent by getting the person with schizophrenia back to work (See Chapter 27). Remember you should not pay for the individual's medication yourself.

5. Discuss delivering medication to the individual's home

You should only discuss this if the individual or family suggests it. You or the Health Extension Worker taking the medicine from the health centre to the individual's home should be a last resort when all the other relevant methods have been tried. The reason for this is that it is not an empowering or sustainable way to ensure the person takes medication. For delivery to the home to be considered, the individual should be very unwell and not attending the health centre. Discuss this with your supervisor.

6. Monitor whether the individual attends the health centre

Each individual should go to the health centre every 1 to 3 months. You should be aware of all the appointments they have, and check if they have attended. If they have missed an appointment, even if you are not due for a home visit, you should make a home visit to check for any problems and remind them to attend. If the individual chooses not to attend the health centre you should discuss the potential benefits of treatment but also listen to their own wishes.

7. Make a plan

At the end of the discussion, make sure that everybody knows what input they are expected to have.

Follow up

Remember to check what progress has been made by the next home visit.

If you agreed to do so, discuss free medication certificate with kebele leader or support travelling to the health centre with community leader/s.

> **Yosef's story**
>
> *Addis tells Berhan that sometimes they can't get to health centre because Yosef is too unwell to walk there. This makes things worse as then he can't get the medication. They agree that to get over this problem they need help to get to the health centre. They discuss who could help. Addis agrees to ask her uncles and cousins to help walk or carry Yosef to main road. They will then get a gari using small amount of money borrowed from uncle*

17.5 Summary

- Some individuals do not access health services due to money or transport problems
- It is important to access the health centre to get treatment, as this may improve the chance of recovery
- Ways to improve access include: exploring the possibility of free medication, making travel to the health centre easier, and improving financial problems.

18 Module: Preparing for a crisis

18.1 What kinds of crises do people with schizophrenia have?

As we learnt in Chapter 14, people with schizophrenia are vulnerable to many sorts of risks. A crisis is when dangerous or worrying incidents happen as a result of these risks. Some common types of crisis include:

- The individual talks about or tries to commit suicide
- The individual is the victim of physical, emotional or sexual abuse
- The individual behaves in a dangerous way. For example they consume poisonous fluid thinking it wouldn't hurt them, or they jump off a roof.
- The individual thinks the family or health worker is trying to harm them and tries to protect themselves in an aggressive way.
- The individual does not want to eat or drink

The individual may feel scared and alone. The family may not know how the best way to look after the individual, and may find it difficult to cope.

18.2 Why do people with schizophrenia have crises?

Many types of crisis happen due to the illness. For example, behaving in an unpredictable way or thinking others are trying to harm them may be due to hallucinations or delusions (experiencing or believing things that aren't true). These are all likely to be worse when the individual is having a relapse (when the illness gets much worse). Other types of crisis are due to stigma or other external factors. For example, being beaten at the holy water. Although these things can happen at any time, they are also more likely to happen when the individual is very unwell.

18.3 Why is it important to prepare for a crisis?

In Chapter 14 you learnt how you should respond when you identify the individual is at risk. We also need to give families advice on how to deal with a crisis when one occurs. It is good to give the family skills to do this because they are the main carers, and they need to know how to cope when you are no longer visiting them. Being well prepared for a crisis helps to avoid a serious or undesirable outcome, for example the individual hurting themselves or someone else, or being chained up.

18.4 How do we help individuals and families to prepare for a crisis?

Principles

It is good to start preparing for a crisis when the individual is well enough to take part in the discussions. This gives the individual more control over the situation. Be aware that talking about future or past crises may be upsetting for the individual and family.

1. Discuss what kinds of crisis are likely

Ask the individual and family to think of crises that have already happened or might happen in the future. Use the examples in Section 18.1 to help them to think about it. Discuss how they will know if a crisis is happening e.g. the individual stops taking medication or becomes angry or upset.

2. Explain why you are doing this module

Explain why you would like to help them to prepare for a crisis.

3. Decide who to ask for help

Ask the individual and family to decide three people to call for support in a crisis. Tell them to keep their phone numbers in more than one place so they don't lose them. The people might include:

- A relative
- A neighbour
- A community or religious leader
- The CBR worker. Remind them that this won't be possible once CBR has finished

4. Make a crisis plan

Decide together what actions the family should take when there is a crisis and record these on the Crisis Management Plan (Form 22). This might include the following:

a) Try to make sure the individual is taking the medication

b) Take the individual to the health centre for a review. Tell the family to make sure they are clear about what the plan is and what to do next. Tell the family to inform you or the health centre staff about violent behaviour, discussion about death or suicide and any beliefs that other people are trying to harm them

c) Contact the relative/neighbour/leader. Just bringing someone else into the home to assess the situation may be useful. They may also help with getting to the health centre if this is difficult.

d) Remove access to weapons and potentially poisonous materials

e) Make the environment safe and comforting. Give the individual lots of space.

f) Remove all access to alcohol and khat.

g) Encourage the individual to put strong feelings and energy into safe activities they enjoy, such as listening to the radio

h) Suggest the individual tries relaxation techniques (See Section 23.4)

5. Discuss how the family should treat the individual during a crisis

- Use a calm, positive but firm tone of voice.
- Use simple language
- Give the individual extra time to respond and to calm down
- Reduce expectations about work or household tasks

6. Discuss what actions are not usually helpful

- The family should try not to argue with the individual. This will only make the person confused and frustrated. Instead the family should be loving. The family shouldn't disagree with delusions. It's ok to tell the person they don't see things the same way and leave it at that.
- The family should not accept violence from the individual. The individual should be clearly told that this behaviour is unacceptable.
- They family should not be violent towards the individual and should try to avoid restraining them.

7. Make a plan to deal with the current crisis, if there is one

Follow up

If there is currently a crisis, make sure you follow up on what has happened and whether the steps helped at the next home visit.

Yosef's story

Yosef and Addis agree that the most likely crisis is that Yosef will become unwell and run away, like has happened in the past. They agree that if a crisis happens Solomon (Yosef's father) will ask their uncle for help. They might also ask the local priest, whose opinion they respect. Berhan writes down the crisis plan. The family cannot read but they appreciate Berhan having the plan written down. This means they can look back at it later with Berhan.

Yosef's Crisis Management Plan

- *Check if Yosef is taking the medication. Gently encourage him to start taking it again if he has stopped.*
- *Go to the health centre for a review, with the help of the uncle.*
- *Tell the local priest if Yosef has run away, in case he can help to find him and bring him home.*
- *If Yosef is still at home, make sure he has some space to himself.*
- *Make sure there is no alcohol or khat in the house.*

18.5 Summary

- It is common for people with schizophrenia to have crises such as dangerous behaviour or thinking about suicide
- It is important to prepare for a crisis to avoid serious outcomes such as getting hurt or being chained
- Ways to prepare for a crisis include: deciding who to ask for support, making a crisis plan, and thinking about ways to treat the individual during the crisis.

19 Module: Dealing with human rights problems

19.1 What human rights problems do people with schizophrenia experience?

All people, including all people with schizophrenia, have the same human rights. Quite commonly people with schizophrenia are denied their human rights. We have already learnt about some human rights problems in Chapter 7, and in Chapter 14 as they are also types of risks and crises. The human rights problems experienced by people with schizophrenia include:

- **Chaining and restraint**
- **Neglect**
- **Physical abuse**
- **Sexual violence**
- **Not accessing health services**

Many people with schizophrenia find it difficult to access health services either for their mental illness or for physical health problems (see Chapter 17). On some occasions people with schizophrenia are actually excluded from getting the same level of health care as people who do not have mental illness.

- **Not being able to work or get married**

Sometimes people with schizophrenia cannot do usual life activities such as working or getting married because they are too unwell. Just as often, individuals find it difficult to do these things due to stigma and discrimination (see Chapter 6).

19.2 Why do people with schizophrenia experience human rights problems?

- **Due to being unwell and the family not having enough support**

People with schizophrenia may get mentally unwell from time to time. This can happen because they are not accessing treatment or because the medication is not sufficient to help them. During these times, if the family has little support they may feel they have no choice but to restrain the individual. Usually, they are trying to protect their family member from running away and being harmed in an accident, or by other people. Often they are also concerned that the individual may harm other people or damage property. When a person is unwell with schizophrenia and the family does not have enough support it also becomes more difficult for the family to look after them properly. It becomes difficult to keep the individual fed and clothed. It can then become more difficult to get them to the health centre, which makes all the problems worse.

- **Not taking medication**

When a person with schizophrenia is not taking their medication, the family may resort to secretly giving the medication (for example mixed into food), tying up or physically forcing the individual to make them take their medication. The person may also be tied up in order to make the journey to the health centre or hospital.

- **Stigma and discrimination**

Many people don't know very much about schizophrenia. They may believe that people with schizophrenia are always violent, or that they are possessed by the devil. These kinds of beliefs can lead people to treat those with schizophrenia badly, such as by beating them or keeping them tied up even when they are well (See Chapter 6).

- **Traditional and religious healing**

Most traditional and religious healing does not harm people with schizophrenia. However, sometimes when a person refuses to take holy water, they may be beaten or tied up so that they can be forced to take the water or bathe in it. Also, some traditional healing practices include physical abuse such as branding, beating and fumigation. All of these things make schizophrenia worse, and are an abuse of human rights.

- **No health services available**

Often the reason why people do not get medical care for schizophrenia is because there is none available locally, or it is too far or too expensive for them to use.

19.3 Why is it important to reduce human rights problems in people with schizophrenia?

People with schizophrenia have the same human rights as all people, so we should ensure they are protected. Reducing human rights issues is also an important way to help the rehabilitation. Whilst they are tied up, individuals cannot begin to undertake usual life activities like washing and dressing, drinking coffee and doing farm work. Treating people with schizophrenia with dignity and respect will improve their self-esteem and confidence and make it more likely they will get back to their usual activities. This in turn reduces the burden on the family.

19.4 How can we reduce human rights problems in people with schizophrenia?

Principles

One of the main aims of CBR is to reduce human rights problems. CBR can do this by:

- Improving access to health services for physical and mental health (see Chapter 17)

- Improving understanding about schizophrenia and changing negative and incorrect beliefs amongst the family (see Chapter 16) and community (see Chapter 31)
- Improving self-care (Chapter 24) and nutrition (see Chapter 21)
- Improving the family environment (see Chapter 25) therefore reducing the likelihood of emotional and physical abuse.

There are also specific steps we can take to reduce human rights problems. Remember that discussing human rights problems may be upsetting for the individual and family. Be sensitive to this and be ready to take a break from the discussions if necessary.

1. Explain why you are doing this module

Explain what human rights are, and that people with schizophrenia have the same rights as other people. Explain why it is important to protect the human rights of people with schizophrenia.

- The individual cannot begin to get back to usual activities whilst tied up
- Treating the individual in a dignified way will improve self-esteem and make it more likely they will do usual activities again

2. Assess human rights problems

You should begin by discussing what human rights issues the individual has faced in the past, or present or may face in the future. You should also refer back to the risk assessment, where human rights issues may have been identified (see Chapter 14).

3. Educate the family about chaining and physical abuse

Advise the family that chaining is not desirable, even if this hasn't happened to the individual. Use the reasons given above (see Section 19.3) for why it should be avoided.

4. Use problem solving to reduce human rights problems

You should then discuss each human rights issue in turn with the individual and caregiver. Encourage them to think of ideas for how to avoid the issue in future using the problem solving approach (see Section 10.5). Discuss the approaches they have already tried. Build on their ideas and then suggest some of your own if everything has not been covered. Some or all of the following methods can be used.

a. Help individuals to take their medication and reduce relapse

Use the strategies in Chapters 20 and 30 to support individuals to take medication and reduce relapse. These will help avoid situations where the individual becomes unwell and the family turn to chaining.

b. Help families to deal with violence and crises

Giving the family strategies to deal with violence from the person with schizophrenia, and other crises situations, will help them to avoid resorting to chaining. See Chapter 18.

c. Educate community members and community leaders

Part of the awareness- raising for community members and community leaders should be that restraint should not be used except in an emergency (see Tasks 5 and 6 in the Community Engagement chapter 31). Ask the family if there is any particular individual who would benefit from education about chaining or physical abuse.

d. Educating holy water priests, attendants and traditional healers

Holy water priests and attendants and traditional healers may be involved in your general community awareness raising. If not, and you feel there are particular problems with priests or attendants you have identified, you may need to undertake specific meetings with these individuals. Ask the family if there is any particular individual who would benefit from education about chaining or physical abuse. The aim is to encourage them to avoid chaining or physical abuse during their healing practices. See Task 11 in Community Engagement Chapter 31.

5. What to do if someone is chained (see also Chapter 14)

If an individual is chained whilst you are working with the family follow these steps:

- Tell your supervisor immediately.
- Facilitate access to the health centre for a clinical review. Ask the family to take the individual as soon as possible.
- If the individual is still chained and they have not attended the health centre after one week, make a joint home visit with your supervisor.
- At this home visit you and your supervisor should discuss with the family and the individual (if possible) the reasons why they have not attended.

- Try to address these reasons, for example:
  - Repeat the importance of receiving treatment for improving symptoms
  - Mobilise community leaders to help the individual travel to the health centre
  - Also see Chapter 17
- If the individual is still chained and they have not attended the health centre after one week, you should inform the Trial Psychiatric Nurse.

Unchaining

Do not tell the family to unchain the individual without support from your supervisor and the health centre. The decision to unchain should be made by the family, preferably in discussions with the health centre.

6. Reducing harm from chaining when it occurs

Despite the best efforts of you, the family and the individual, sometimes the individual may become so unwell that the family still feels the best option is to tie up the individual. You should inform your supervisor and facilitate the individual to attend the health centre for a review. You should not encourage chaining, however you can offer the following advice to the family to ensure that chaining is as safe and dignified as possible when it does occur.

- Make sure the individual is in a sheltered position not exposed to the sun and rain.
- Make sure the individual has some clothing and/or blankets to keep warm
- Make sure the individual is offered regular food and water. Help and encouragement should be given to eat and drink.
- If possible, allow the individual to be unchained every day to walk around
- Change the position of the chains/ restraint as often as possible, to ensure the individual is not always sitting or lying in the same position
- Check regularly for wounds or sores caused by the chains/restraint, or by lying in the same position for a long time. Carefully wash any wounds or sores with clean water. Change the position of the chains to allow the wounds to heal.
- Continue interacting with the individual and involving them in conversation
- If the individual becomes physically unwell, take them to the health centre

7. What to do when other human rights abuses occur

- **Neglect.** See Chapter 35.
- **Physical abuse.** See Chapter 35.
- **Sexual violence.** See Chapter 35.
- **Not accessing health services.** Complete Module: Improving Access to Health Services (Chapter 17).
- **Not being able to work or get married.** Consider completing Modules: Getting back to work (Chapter 27), Taking part in community life (Chapter 26) or Dealing with Stigma and Discrimination (Chapter 28) depending on the situation.

8. Make a plan

At the end of the discussion, make sure that everybody knows what input they are expected to have before the next session.

Follow up

Check what progress has been made relating to human rights problems at the next home visit.

Yosef's story

In the past when Yosef was very unwell Addis and Solomon felt it was necessary to chain Yosef to the house. The kebele chairperson also told them it was their responsibility to this, to protect other people and property in the kebele. Yosef and Addis find it upsetting to talk about him being chained up. Berhan tells the family that she understands that they were only trying to help Yosef and protect other people when they chained him up in the past. However, it is important to try to prevent this happening in the future. The family agree that the main way to avoid chaining is to ensure Yosef takes his medication regularly. They agree that in the future they will complete the Module: Supporting individuals to take medication. Solomon and Addis feel they cannot have any influence on the kebele chairperson. Berhan agrees to discuss the issue with him. Berhan also gives the family advice on how to chain Yosef in a way which is as dignified as safe as possible, in case they ever need to do it as a last resort.

19.5 Summary

- Quite commonly people with schizophrenia are denied their human rights, for example they are chained or physically abused.

- Respecting the human rights of people with schizophrenia is empowering and makes it more likely they will get back to usual activities.
- You can reduce human rights problems by: educating the family, supporting adherence, helping families to deal with crises, and educating community leaders and traditional healers.
- There are ways to reduce the harm from chaining when it does occur.

.

20 Module: Supporting individuals to take their medication

20.1 What problems do people with schizophrenia have in taking their medication?

Some people with schizophrenia refuse to take any treatment from the beginning. More commonly, people start the medication but then stop taking it regularly, or stop it altogether, over a period of time.

20.2 Why do some people with schizophrenia not take their medication?

Individual factors

- Insufficient support from the family
- The individual feels shame or stigma from taking the medication, especially if they no longer have symptoms and wish to be seen as well
- The individual and/or their family believe that the illness is due to spirit possession or other causes, and that medication will not help this.
- The individual and/or their family understand that medication helps to relieve symptoms in the short term but do not understand the importance of medication to prevent the illness coming back.
- Holy water priests or attendants or traditional healers tell the individual that it is not needed, or even harmful, to take medication whilst receiving traditional treatment

Illness factors

- People with schizophrenia may not understand that they have an illness that needs treatment. Because of their illness, they may even believe that the treatment is being given to them to harm them.
- People with schizophrenia often have low levels of motivation
- People with schizophrenia often have low levels of attention and concentration
- The person may also be using khat or alcohol. This may make them less organised or less interested in taking medication.

Treatment factors

- The medication causes unpleasant side effects in the person with schizophrenia
- The person with schizophrenia does not have enough food to cope with feeling more hungry than usual as a result of the side effects of the medication
- The person with schizophrenia does not have a good, trusting relationship with the nurse or doctor prescribing the medication

- The medication has to be taken on a complicated schedule so the person with schizophrenia forgets to take the medication.
- The medication does not completely cure the illness so the individual does not see the point in taking it.

Practical factors (see Chapter 17)

- The family cannot afford the medication
- The family cannot afford to travel to the health centre
- It is difficult to take the individual to the health centre because they are unwell and/ or it is too far.
- There is no one free to take the individual to the health centre.
- The medication is not always available at the health centre

20.3 Why is it important to take medication regularly?

It is important to take medication regularly to treat the illness so that the person with schizophrenia starts feeling better. When individuals take medication they are more likely to be able to get back to usual activities like farm work and housework. Once taking the medication, it also is much less likely that the individual will be so unwell that the family needs to restrain them up. It is very important to continue to take medication, even after the person with schizophrenia is feeling well again. This is to prevent the illness from coming back. If the illness comes back the individual may stop being able to work and socialise again. Even though there are some disadvantages to taking medication, for example the side effects, most people with schizophrenia feel on balance that life is better when taking the medication.

20.4 How can we support people with schizophrenia to take their medication?

Principles

- There is not one single approach to helping people to take their medication. Often a range of methods is needed for each individual.
- Which methods you use depends on their social and personal circumstances
- When somebody does not take his or her medication, it is usually not a problem that can be quickly 'solved'. This means ongoing input is needed, usually for the duration of receiving CBR.
- You should not hassle or force the individual to take medication. The individual should continue CBR even if they are not taking medication for any reason.

1. Revise the information about medication

Revise the following information about medication.

- What is anti-psychotic medication
- Side effects of anti-psychotic medication

2. Explain why you are doing the module

Explain why it is important to take medication regularly and why is it important to continue taking medication when feeling well.

3. Assessing problems with taking medication

Find out what the current arrangements are for taking medication. When do they take it? Does anyone remind them? Then assess the reasons why the individual has problems taking the medication, or might have problems in the future. Ask what their attitude towards medication is. Have they found it useful? The Checklist for problems taking medication (Form 15) should be used. This covers the reasons in Section 20.2.

4. Problem solving to improve taking medication regularly

You should then discuss each reason or problem in turn with the individual and caregiver. Encourage them to think of ideas for how to get around the problem using the problem solving approach (Section 10.5). Discuss the approaches they have already tried. Build on their ideas and then suggest some or all of the ones below, if they have not been covered:

a. Involve the family

The individual's immediate family are the most important people to help them take medication regularly. You should:

- Try to understand the family member's viewpoint and attitudes towards medicines
- Involve them in understanding the reasons for not taking medicines and deciding the best methods to improve the situation
- Provide them with information on the benefits and side effects of medicines.
- Involve members of the extended family (uncles, aunts, cousins, etc) who live outside

the home. They may have a significant role to play in ensuring individuals take medication regularly. Find out who the important individuals are and consider meeting with them to gain their support.

- Get the family to use positive feedback
- Give the family skills to deal amicably with situations where the family members and the individual have different views on adherence (see Chapter 25)
- Consider asking the family to observe the individual every time they take the medication. However this may not help the individual to feel independent, so try this only if this the other ideas do not work.

b. Reinforce information about medication

Give or revise information about medications, their benefits and side effects. You may need to give information to other family members as well as the individual and caregiver.

c. Address side effects

Discuss concerns about side effects. There are some side effects you can help with and others that need to be reviewed at the health centre (see Table 4).

Table 4 How to help with side effects

What happens?	How you can help
The mouth is very dry	Try putting a clove in the mouth or sipping water
A lot of saliva is produced	Advise them to put a cloth next to their mouth at night
Constipation	Advise them to drink lots of water and eat high fibre foods e.g. lentils and vegetables
The person feels dizzy when they go from lying down or sitting to standing	Advise person to get up slowly and wiggle fingers and toes before getting up
The person wants to eat more than usual	Advise that the individual probably does not need more food, this is just a side effect of the medication. Try eating small amounts regularly and including more bulky food e.g. vegetables. Refer to health centre- for change of dose or possibly medication.
The person feels restless and cannot sit still.	Refer to health centre
The head, neck or body becomes stuck in an unusual position.	Refer to health centre
The hands shake	Refer to health centre
The person moves very slowly and feels their muscles are very stiff	Refer to health centre
The person is very drowsy	Refer to the health centre- the amount of medication may need to be changed, or the time of day they take it. Drowsiness may reduce when the person gets used to the medication
Writhing movements of the head, neck, arms or legs.	Refer to health centre
Seizures	Refer to health centre
Sexual problems	Refer to health centre
Skin rash	Refer to health centre
The person cannot pass urine	Refer to health centre
Suddenly developing stiff muscles, fever, and confusion	Take to hospital immediately

d. Get a daily routine

Build medication into the daily routine. Suggest the person with schizophrenia always takes the medication after doing a daily task e.g. getting washed in the morning or having breakfast.

e. Use positive feedback

People with schizophrenia have problems with motivation. They need frequent and consistent

feedback and appreciation from family members and you for continuing to take the medication. To begin, the family can reward the individual taking his medication with clear signs of appreciation (by telling them they are doing well or cooking his favourite dish). Make it clear that this is linked to the fact that the individual has been taking the medicines.

f. Improve relationship with the nurse

When you attend the health centre with the individual around the time of each CBR Review, one of your roles is to help improve the communication and relationship with the nurse. If there is any obvious misunderstanding during the consultation, try to clarify this at the time. You can also help the individual to raise any issues, for example that the medication schedule is too complicated. After the consultation, check the individual and the caregiver understood everything and fill in any gaps.

g. Arrange a medication review at the health centre

Taking the medication once daily is likely to be easier to remember. If the individual feels that the schedule is too complicated, they can discuss this with the nurse at the health centre. Having the injection instead of tablets usually makes it more likely the individual will take the medication, as they don't have to remember every day. On the other hand some people with schizophrenia are less willing to have injections than to take tablets. It is not your decision to change to injections. However, if other methods have been tried but are not successful, and the individual is willing to try injections, you can encourage him or her to speak to the nurse about this at the health centre.

h. Engage with traditional and religious healers

Try to find out who the healer is. You may have already made contact with them during your initial community engagement (see Task 5 in Community Engagement Chapter 31). With the permission of the family, try to arrange a meeting with the healer. Give them information about schizophrenia, including that medication can help to reduce symptoms and prevent the illness from returning. Tell them that the individual does not need to stop receiving holy water or traditional practices, but that they can use medication alongside it (see Task 11 in Community Engagement Chapter 31).

i. Explore accessing free medication

See Chapter 17.

j. Make travelling to the health centre easier

See Chapter 17.

k. Address financial problems

See Chapter 17.

Table 5 can be used as a guide for which methods to use:

Table 5 Approaches for supporting individuals to take medication

Problem	Approach/s
Insufficient family support	Involve the family
Shame or stigma	Give information<br>Involve the family
Differing beliefs	Give information<br>Involve the family
Lack of understanding about preventing relapse	Give information<br>Involve the family
Discouraged to take medication at holy water	Give information<br>Engage with traditional and religious healers<br>Involve the family
Lack of awareness (due to illness)	Give information<br>Involve the family
Lack of motivation and/ or concentration	Positive feedback<br>Daily routine<br>Involve the family<br>Medication review at health centre
Khat or alcohol use	Referral to health centre<br>Positive feedback when khat or alcohol is avoided<br>Get a daily routine<br>Involve the family
Side effects	Address side effects
Insufficient food for increased appetite	Small regular meals including vegetables
Poor relationship/communication with nurse	Improve relationship with nurse
Complicated medication schedule	Medication review at health centre
Cannot afford medication	Explore free medication<br>Address economic problems
Cannot afford travel to health centre	Make travelling to the health centre easier<br>Address economic problems
Practical issues travelling to health centre	Make travelling to the health centre easier

5. Difficult situations

What to do when the individual refuses to take the medication

If the individual refuses to take the medication you should listen carefully and respect their point of view. You should try the approaches above. However, sometimes the individual will continue to refuse. If this happens you should not hassle them or force them to take it, but should refer them to the health centre for a review. It is important to remember that you should continue with CBR even if the individual is unable or unwilling to take the medication.

Giving the individual medication without their knowledge

The caregivers may have previously given the medication without the knowledge of the person with schizophrenia, for example by mixing it in food or tea. The family may also suggest this as a method for dealing with them not taking the medication in the future. This is a difficult situation as the individual's basic right to decide what treatment they receive is not respected. It also means that if the individual gets better they do not realise that it is due to the medication. This makes it even less likely they will take the medication out of choice later on. In addition, if the individual finds out later on that they were being given the medication in secret, they may find it difficult to trust people. This might make the illness worse.

On the other hand, it is usually understandable when families take this step, if they feel there is no other way to get the person to take the medication, and therefore get well. You should not encourage the family to give the individual medication without their knowledge. If this is done or suggested, you should explain to the family why this method should be avoided if possible, without appearing to be judgemental. Your role is then to ensure that all the other methods for helping the individual to take medication are used first. Tell your supervisor if you are aware if it is happening.

Forcing individuals to take medication

The caregivers may have previously used physical force to get the person with schizophrenia to take the medication, for example, by tying up the individual and forcing the medication into his or her mouth. Others may suggest this as a method for future use.

You should encourage families never to force individuals to take medication in this way. As well as being disempowering, it may make the person more agitated, or they may even get harmed. Tell your supervisor if you are aware if it is happening.

6. Make a plan

At the end of the discussion, make sure that everybody knows what input they are expected to have. Set some specific tasks for the individual and family to complete by the next home visit. For example:

- The person with schizophrenia agrees to take the medication with breakfast everyday
- The caregiver agrees to remind the individual to take medication with breakfast, observe whether they take it or not and to give encouragement if he or she takes it
- The CBR worker agrees to meet with the holy water priest and attendant and give information about the importance of taking medication whilst receiving holy water

Yosef's story

Yosef says even when he is well he often has problems remembering to take his medication. The medication makes him feel tired, drool saliva and makes his hands shake. Sometimes Yosef goes to holy water. The holy water attendants tell him he shouldn't use medication at the same time as this will mean the holy water will not work.

Addis and Yosef agree that Yosef needs the family's help to remember to take the medication. They agree Addis will remind him and watch him take it at breakfast every day. When he takes it she and Solomon will give lots of encouragement. Berhan advises Yosef to try putting a cloth next to his mouth at night, to help with the drooling. She also asks him to go to the health centre in case the amount of medication can be reduced, or he can take it at night. Berhan agrees to discuss the importance of continuing medication with the holy water priest and attendant. She discusses this with her supervisor, Haile, and they go together to the holy water site. It takes Berhan and the family two home visits to discuss medication and create the plan.

7. Monitoring medication

You should assess whether medication is being taken at some point during every home visit, even if you are not currently working through this module. Ask both the individual and the caregiver if there have been any problems. Be sensitive and respectful and do not hassle the individual to take the medication. Use problem solving to address any problems, or complete or repeat this module. Record issues with medication on the Home Visit Form (Form 11).

Follow up

Remember to check what progress has been made at the next home visit. If relevant, refer to the health centre, or meet with community leader, kebele leader or traditional or religious healer.

20.5 Summary

- Many people with schizophrenia have problems taking their medication due to: individual reasons (for example, not enough family support), illness factors (for example, not being organised), treatment factors (for example, side effects) and systemic factors (for example, cannot afford travel to health centre)
- Taking medication is important for getting well and staying well
- Ways to help individuals take medication include: involve family, give information, get a daily routine, use positive feedback, educate traditional healers, arrange medication review at the health centre and address side effects.

21 Module: Improving physical health

21.1 What problems with physical health do people with schizophrenia have?

- **Usual health problems**

People with schizophrenia experience the same physical health problems as other people. For example, they may get malaria, a cough or a broken bone from an accident. However, people with schizophrenia are less likely than other people to get medical help for any physical health problems they have.

- **Other disabilities**

People with schizophrenia are just as likely to have other disabilities as other people. For example they may be blind or have problems walking due to childhood polio or an accident.

- **Being underweight or underweight**

In Ethiopia, people with schizophrenia are commonly underweight. Much less commonly in Ethiopia, people with schizophrenia may become overweight.

- **Reproductive health and sexual health problems, need for family planning and antenatal care**

Like anyone else, people with schizophrenia have sexual relationships. Women with schizophrenia may also be sexually exploited. They therefore need access to advice about safe sex, family planning and antenatal care just like other people.

- **Chewing khat, drinking alcohol or smoking**
- **Physical injuries**

People with schizophrenia may develop wounds and sores from being beaten or being chained up. Their muscles may stop working properly if they are chained up for a long time.

- **Heart disease and diabetes**

People with schizophrenia are more likely to get heart disease and diabetes (high blood sugar levels) than other people.

21.2 Why do people with schizophrenia have problems with physical health?

There are a number of reasons why people with schizophrenia have more physical health problems than other people.

- **Difficulty accessing the health centre**

It may be difficult for families to take the individual with schizophrenia to the health centre, either because they have difficult behaviour or because they are tied up.

- **Difficulty paying for health care**

Families may have problems paying for health care, particularly if the individual is so unwell that they or the caregiver have difficulty working.

- **Side effects of medication**

Medication can cause problems such as gaining weight and sexual problems. Sometimes people do unhealthy behaviours, such as chewing khat or drinking alcohol, to try to reduce the side effects of medication.

- **Not enough food**

People with schizophrenia may become underweight because the family is poor and they do not have enough food.

- **Stigma and discrimination**

Sometimes the family or community believes the individual does not need or deserve the same nutrition or physical healthcare as other people. Sometimes health workers don't take people with schizophrenia seriously and dismiss their physical complaints.

- **Individual not willing or able to eat regularly**

Sometimes due to the illness the individual does not want to eat or doesn't find time to eat

- **Being inactive**

Many people with schizophrenia become inactive. This can be one reason for weight gain.

- **Trying to escape problems or socialise**

People with schizophrenia may drink alcohol, chew khat or smoke to try to escape their problems and socialise.

21.3 Why is it important to improve physical health in people with schizophrenia?

People with schizophrenia are much more likely to die young than other people. This is partly due to people with schizophrenia committing suicide. However, it is mainly due to physical health problems such as infections and malnutrition. Having physical health problems may stop people recovering from schizophrenia. When people have physical health problems it may be more difficult to get to the health centre for anti-psychotic medication, or to return to farm work. It is therefore important to try to improve physical health in people with schizophrenia. Chewing khat can result in decay of the teeth, heart problems, cancer, anxiety and depression. Both khat and alcohol make the symptoms of schizophrenia worse and make it harder to recover. It is therefore important to support people to stop using khat and alcohol.

21.4 How can we improve physical health in people with schizophrenia?

1. Explain why you are doing this module

Explain to the individual and family what physical health problems are common in people with schizophrenia. Give reasons why people with schizophrenia have these problems.

2. Assess physical health problems

Assess what physical health problems or unhealthy behaviours the individual has or might have in the future.

3. Problem solving to improve physical health

You should then discuss each problem in turn with the individual and caregiver. Encourage them to think of ideas for how to get around the problem using the problem solving approach (see Chapter 10). Discuss the approaches they have already tried. Build on their ideas and then suggest some or all of the ones below, if they have not been covered:

a. Ensure access to health care

Ensure that the individual is accessing the health centre or hospital for physical health problems when they arise, for example infections or accidents. See Chapter 17.

b. Avoid alcohol, cigarettes and khat

Encourage the individual not to drink alcohol, smoke or chew khat. Explain that this will probably make the illness worse. Ask the family to try to avoid giving the individual money for alcohol, khat or cigarettes, if possible. Discuss with the individual whether they need help to cut down or stop.

c. Get help for problems with alcohol or khat

If the individual has serious problems with alcohol or khat use, then you should refer them to the health centre. A person has a serious problem if:

- They often appear to be affected by alcohol or khat. For example, smells of alcohol, looks intoxicated, staggers when walking, slurred speech.

- Because of alcohol, they often injure themselves, for example from falling down, fights, or walking in traffic
- They have physical symptoms from excessive alcohol use. For example can't sleep, very tired, can't eat, nauseated by food smells, vomits, complains of a bad stomach, diarrhoea, headaches.
- They show signs of drug use. For example, skin infection, unkempt appearance, dry mouth, burned lips, bad or worn teeth, complains of *dukak* (vivid unpleasant dreams)

d. Encourage a healthy diet

Encourage the individual to eat a healthy diet as much as possible. Be aware that many families will have very limited choice about what they eat. A healthy diet consists of a mixture of:

- Cereals, such as teff (injera), barley and wheat
- Pulses, such as lentils
- Fruits
- Vegetables
- Small amounts of milk, cheese and yogurt
- Small amounts of meat

Make sure the person with schizophrenia eats the same amount and type of food as the rest of the family (See Chapter 24). If you are concerned the person is very underweight you should refer them to the health centre.

e. Encourage appropriate physical activity

Encourage the individual to do some physical activity as it is not good for them to sit at home all day. Most people can do this as part of their day-to-day activities, for example going to fetch water. However, people with schizophrenia should not do excessive exercise as this may contribute towards them becoming underweight.

f. Refer to the health centre for problems with side effects of medication

You should refer back to the health centre if the individual side effects which are affecting the individuals' physical health. For example, problems with menstrual periods.

g. Refer to the health centre for malnutrition

Refer the individual to the health centre, and facilitate them to attend, if they appear very underweight or malnourished (see Chapter 14).

h. Attend to physical injuries

If the individual has any wounds or sores make sure these are washed with clean water. If you are worried about the wound, for example if it is not healing, refer the individual to the health centre. If the individual has problems moving their arms or legs properly because they have been chained up for a long time you should refer them to the health centre. You may be asked to help them exercise their arms and legs.

i. Attend to physical disabilities

If the individual has a physical disability discuss with your supervisor the best way to support them.

j. Ensure access to the health extension programme

The Health Extension Worker should make routine visits to the household to cover contraception, protection against HIV, sanitation, malaria prevention, child nutrition and antenatal care. If the individual asks for help with any of these issues, or if you know that the Health Extension Worker has not made routine visits to the household, contact the Health Extension Worker. Ask her to make a routine visit to the individual to discuss these issues.

4. Make a plan

At the end of the discussion, make sure that everybody knows what input they are expected to have. Set some specific tasks for the individual and family to complete.

Follow up

Make sure you refer to the health centre or health extension worker if this is relevant. If you do make a referral make sure that the individual attends for the initial visit and any follow up that is needed. At later visits ask about progress with healthy behaviours. If there are physical disabilities, discuss with your supervisor.

> **Yosef's story**
>
> *Berhan observes that Yosef is quite underweight. Yosef says sometimes he is too tired to eat. Berhan tells Addis about the different parts of a healthy diet. Addis explains that often they cannot grow or afford to buy different types of grains and vegetables and they rarely eat meat. Berhan tells Addis she understands they have limited choices. They agree that Yosef needs more support and encouragement at meal times. Berhan discusses the issue with Haile. They agree that Yosef does not need to go to the health centre at the moment, but if he loses more weight this will be necessary.*

21.5 Summary

- People with schizophrenia have physical health problems such as being underweight and using khat and alcohol
- Physical health problems may be dangerous, and make it more difficult to get well
- We can improve physical health by: ensuring access to health centre and health extension worker, and giving advice about diet, exercise, alcohol and khat.

22 Module: Dealing with distressing symptoms

22.1 What distressing symptoms do people with schizophrenia have?

As we discussed in Chapter 2 people with schizophrenia can have four groups of symptoms:

a. Hallucinations (seeing or hearing things that aren't really there) and delusions (believing things that aren't true)
b. Problems with motivation
c. Problems with thinking clearly
d. Person not being aware that they have an illness

All of these symptoms can be very distressing for individuals. The extent to which they are distressing varies from person to person.

22.2 Why do people with schizophrenia have distressing symptoms?

Distressing symptoms are part of the illness of schizophrenia. Medication usually helps to reduce hallucinations and delusions, but sometimes they can continue even when an individual is taking the medication regularly. It is common for problems with motivation and problems with thinking clearly to continue even when an individual is taking their medication regularly.

22.3 Why is it important to reduce distressing symptoms?

As well as being distressing for the individual, these symptoms can be disabling. See Chapter 4. Having symptoms can make getting back to usual activities more difficult.

22.4 How can we help to reduce distressing symptoms?

Principles

At all times, the focus should be on **empowering** the individual to take charge of managing their own illness. The general approach to reducing symptoms is:

- Supporting the individual to take medication (see Chapter 20)
- Reducing **stress** by improving the environment, for example dealing with human rights problems (See Chapter 19), or improving coping strategies (see Chapter 23)

However, people with schizophrenia often continue to experience distressing symptoms despite taking these steps. It is therefore important to have specific ways to help to cope with them. To do this, follow these steps.

1. Revise information about symptoms

Revise what kinds of symptoms people with schizophrenia experience, relating them to the symptoms the individual has

2. Explain why you are doing this module

Explain why it is important to reduce distressing symptoms. Emphasise that this is a chance for the individual to feel better. Highlight that problems with motivation and thinking clearly can be difficult to overcome.

3. Assess symptoms

Find out which is the most distressing symptom and focus on that first. Discuss the following issues relating to that symptom:

- How often it happens
- How it affects the person
- What makes it worse, for example having an argument with parents
- What makes it better, for example talking to a brother or sister

4. Suggest potential coping strategies

a. Hallucinations

- *Change the level of activity*
  Try relaxation (see Chapter 23), or taking a walk
- *Do a distracting activity*
  Talk to family members or neighbours, listen to the radio or sing a song or hum
- *Talk to yourself in a positive way*
  For example, 'I can cope with this'
- *Ignore the hallucination as much as possible*

b. Delusions

- Suggest that the individual checks their ideas of reality with someone they trust, for example the caregiver
- If the individual discusses their delusions with the caregiver, the caregiver should listen to them. The caregiver should not try to argue or reason with them.

c. Problems with motivation

- Remind the family that the person is not lazy or useless, but has an illness that makes it difficult to find the energy or will power to do their usual activities.
- Encourage the individual to have a regular routine, e.g. getting up at a regular time, eating when others eat (see Section 24.4 for more detail)
- Encourage the family to involve the individual in social and community activities, for example attending church (see Chapter 26)
- Encourage the individual and family to think about the future with hope. Ask them to think about the progress that the individual has already made.

d. Problems with thinking clearly

- When the person with schizophrenia is doing something, for example preparing food or having a conversation, try to reduce distractions. For example turn off the radio and reduce background conversations.
- Try activities that improve concentration, for example weaving or praying
- Remind the family to give the individual lots of time to do small tasks and not to become impatient.
- Practice activities many times until they become easier

5. Discuss coping strategies that are unlikely to be helpful.

These include: Angrily telling the voices to go away; Drinking alcohol or chewing khat; Sleeping a lot; Avoiding contact with other people and Stopping medication.

6. Make a plan

Help the individual to select a coping strategy or strategies, involving the caregiver in the discussion. You can also use a problem solving approach (see Section 10.5). Ask the individual to practice the coping strategy they have chosen a few times. Ask to them to think about times when it is possible or not possible to use the strategy.

Follow up

Follow up progress every time you see the individual. Ask whether it is helping or not. If it is not helping, make changes to the strategy or try a different strategy.

Sara's story

Sara is a young woman who became unwell with schizophrenia one year ago. She can still hear voices that command her to do things. Sometimes she believes she has special powers to read other people's minds. Her husband, Alemu, usually tells Sara that she is stupid or mad when she talks about this. These problems are a bit better since she started taking the medication, but they have never completely gone away. Berhan asks Sara to describe these problems and to tell her how often they happen. Sara tells Berhan that the problems get worse when she doesn't take the medication and when she gets stressed. After some discussion, Sara agrees to try listening to the radio when the voices next become very bad. Alemu agrees that it doesn't seem to help when he insults Sara or tells her off when she talks about her special powers. Alemu agrees to try not to do this in future. Berhan asks Sara and Alemu to try these ideas and that they will discuss how it went at the next home visit.

22.5 Summary

- Many people with schizophrenia find their symptoms distressing, even when they are taking medication regularly.
- Having distressing symptoms can make it more difficult to get back to usual activities
- We can try to reduce distressing symptoms by supporting individuals to take medication, and reducing life stress.
- We can also suggest coping strategies such as: doing a distracting activity (for hallucinations), discussing ideas with a trusted person (for delusions), having a regular routine (for motivation problems) and reducing background noise (for problems thinking clearly)

23 Module: Managing stress and anger

23.1 What problems do people with schizophrenia have with stress and anger?

Stress is what people experience when they feel overwhelmed by the things happening in life. It makes people feel tense and anxious and that they cannot cope with the difficulties they face. We all experience stress in our daily lives. However when someone has schizophrenia this will increase the stress of the individual and their family members. Stress can have many effects on people, including changes in the body, emotions, and behaviour. In particular stress can sometimes cause people with schizophrenia to become angry. Anger can sometimes cause an individual to harm other people or themselves or damage property.

23.2 Why do people with schizophrenia have problems with stress and anger?

Different people find different things stressful. Stress can be caused by:

- Important life events that happen suddenly, for example a death in the family,
- Ongoing difficult situations, for example financial problems or family conflict. These things may be more common when someone in the family has schizophrenia.

23.3 Why is it important to reduce problems with stress?

Stress, along with **vulnerability**, is one of the factors that determines how severe the illness is and how many relapses there are (see Section 2.3). Learning to manage stress is therefore important for the wellbeing of the individual and to help them to recover. Anger can cause relationship problems within the family. If a person with schizophrenia becomes aggressive, the family may feel it necessary to tie them up in order to protect other people and the individual. In order to prevent this happening it is important to learn how to prevent and deal with anger.

23.4 How can we help to reduce problems with stress and anger?

Principles

Problems with stress and anger can be reduced by:

- Preventing stress and anger happening in the first place
- Giving ways to deal with stress and anger when it does happen

1. Give information about stress and anger

Explain what stress and anger are and why they happen

2. **Explain why you are doing this module**

Explain why it is important to reduce stress and anger

3. **Assess problems with stress**

Discuss what situations were stressful in the past or might be stressful in the future.

4. **Discuss ways to prevent stress**

Discuss how the individual can avoid feeling stressed in the future. Use the individual and families' ideas and suggest the following if they have not been covered:

a. **Avoid or try to change situations that were stressful in the past**

If a situation was stressful before, it is likely to cause problems for the person when they are in the same situation again. Suggest to the individual that they avoid these if possible. For example if the individual finds it difficult to attend a wedding, they could avoid going to the ceremony. Or suggest that they change the situation to make it less stressful. For example, they could meet the bride and groom at a different time.

b. **Have regular activities**

Stress can be reduced if the person has the right balance of activity and rest. People with schizophrenia usually find it helpful to have some structure to the day. Try to involve the individual in meaningful activities, such as housework, farm work or drinking coffee with others. See also Chapter 24.

c. **Have a healthy lifestyle**

Eating enough nutritious food, getting enough sleep, doing exercise and not drinking alcohol or using khat can all help to reduce stress. See also Chapter 21.

d. **Have reasonable expectations**

Having high expectations of the individual can be stressful for him or her. Encourage the family to have reasonable expectations and for realistic goals to be set.

e. **Try not to be critical**

It is important not be critical of the individual and for them not to be critical of themselves.

Encouragement should be given for any task that the individual does and any small changes should be acknowledged. Think of positive things about the person and remind them of these when they are feeling negative. Ask the family to do the same.

5. Discuss ways to deal with stress

Discuss how the individual deals with stress when it does happen- is it helpful? Use the individual and families' ideas and suggest the following if they have not been covered:

a. Allow the individual to discuss their feelings

Letting the person talk about their feelings often provides some immediate relief and can keep stress from building up. Stress may be an early warning sign of a relapse. If the person with schizophrenia can tell you or a relative when he/she is feeling stressed, the relapse may be prevented. See Chapter 30.

b. Allow the family to discuss the situation

Discuss the situation with the family and try to find possible solutions to the problem causing the stress. Use the problem solving approach (see Section 10.5).

c. Use relaxation techniques

Relaxation techniques can help in reducing stress and sleeping well. You can teach both the individual and family these techniques. It is best to practice every day.

Controlled Breathing

- Explain that when we become stressed our breathing rate often speeds up. Slowing down our breathing can help us to feel more comfortable.
- Show the individual and family how to breathe in for 4 counts, hold for 2 counts and breathe out for 6 counts. You can close your eyes if you wish.
- Practice this exercise in the session for 5 or 10 minutes.
- Ask for feedback from the client and family about how they found the experience and discuss any difficulties.
- Discuss that learning a slow breathing technique is more difficult than it first seems, and requires a lot of practice. You could also suggest some ways of making the process easier, for example:

- Initially, only practice the breathing exercise when you're not feeling stressed
- Explain that some people describe feeling more stressed when they first begin using controlled breathing. It is important to carry on with the technique, because this feeling of stress will decrease with practice
- Practice in a comfortable environment. Lying down is easier than sitting in a chair. Choose a quiet dimly lit room, and try to practice at a time when they will not be disturbed
- The breathing pattern may be hard for some people so you might need to adjust it (e.g. breathe in for 3 counts, hold for 1 and breathe out for 5, or breathe in for 3 counts, hold for 1 and breathe out for 3 counts)

Muscle relaxation

The aim of this form of relaxation technique is to help the person relax by releasing muscle tension with each outward breath. It builds on the controlled breathing technique described above. This is what you should say:

"Focus on your breathing…Just focus on each breath, in and out...As you breathe in, tense the muscles in your feet as tight as you can. As you breathe out relax your feet and let go of the tension"

You then go through each specific areas of the body in turn, continuing with the ankles and working upwards. Repeat the instruction to tense the particular area as you breathe in and release any tension as you breathe out. Continue this exercise for 10-15 minutes.

d. Religion

For many people religion gives comfort and direction to their lives. Religious beliefs, prayer and attending church or mosque can help people cope with stress. Also, religious groups, for example *mahaber*, can be a source of social support, which can reduce feelings of isolation and stress.

e. Encourage the individual to talk about themselves in a positive way

Encourage the person with schizophrenia to stop talking to themselves in a negative way, for example "This is awful". Encourage them to try talking to themselves in a positive way, for example "This is a challenge but I can handle it" or "I am going to do the best I can".

f. Keep a sense of humour

Many people with schizophrenia have a good sense of humour. When a stressful situation happens, try to say something that will make the individual smile, to avoid them being totally overwhelmed by the situation.

g. Exercise

Physical activity has a positive effect on reducing stress, lifting mood and improving sleep patterns. People with schizophrenia should try to do some light physical activity every day, for example walking. However they should not do strenuous activities or exercise for long periods as this may contribute to weight loss (see Section 21.2).

6. Discuss unhelpful ways to deal with stress

These include: smoking, drinking alcohol, chewing khat, not spending time with other people, being very busy and getting angry with other people.

7. Discuss problems with anger

Discuss what situations have made the individual feel angry or what might do in the future.

8. Discuss ways to prevent anger

Some ways to prevent anger include:

- Try to understand what makes the individual angry and try to address this problem
- Prevent and deal with stress (see above)
- Develop self-confidence in the individual (see Chapter 28)
- Make sure the individual is accessing the health centre and taking their medication
- Try to avoid drinking alcohol or using khat; these can reduce people's anger control.

9. Discuss ways to deal with anger when it happens

Suggest the individual tries some or all of the following techniques when they feel angry:

- Leave the situation which is causing him or her to feel angry as soon as possible
- Notice the fact that he or she is angry. Stop doing whatever they were doing and either walk around or sit calmly for a few minutes.

- Release the stress in a way in which there is least possible harm to self, others, and the environment, for example punching a pillow.
- Do relaxation techniques, for example controlled breathing.
- If possible do something distracting, like watering the kitchen garden
- Talk to someone about what is making them feel angry.

If he or she became aggressive, after the situation has passed, suggest that individual: Apologises to anyone involved and tries to tidy up or fix anything that was disturbed

10. Make a plan

Help the individual to select strategies for preventing with and dealing with stress and anger. Involve the caregiver in the discussion.

Follow up

Remember to review which of these ways to deal with stress or anger were helpful, and which were not. Suggest to the individual and family to focus on the helpful ones in future.

23.5 Summary

- Everyone experiences stress sometimes, but it is more common in people with schizophrenia. Stress can lead to anger.
- Stress can make symptoms worse and make relapse more likely
- Ways to prevent stress include: avoid stressful situations, healthy lifestyle, have reasonable expectations
- Ways to deal with stress include: relaxation, religion and talking about problems
- Ways to prevent and deal with anger include: developing self-confidence, leaving the situation that is making them angry, and releasing the anger in a way that is not harmful

24 Module: Improving day to day functioning

24.1 What problems do people with schizophrenia have with day-to-day functioning?

People with schizophrenia may spend the day doing nothing in particular. This can be distressing for family members and they may see the person as being 'lazy'. People with schizophrenia may also have problems doing specific tasks. They may have problems with self-care, including: Cleaning teeth, Washing their body and hair, Eating food in a proper manner, Washing hands before and after eating, Cutting nails and Using the toilet properly.

They may also have problems with household tasks, including: Preparing food, Preparing coffee, Cleaning the cooking and serving utensils, Fetching water, Going to the mill house to get grain ground, Washing clothes, Cleaning the animal area, Preparing local beverages, for example tella, for the household, fencing and repairing the house.

24.2 Why do people with schizophrenia have problems with day to day functioning?

People with schizophrenia may have problems doing these tasks for a variety of reasons:

- The illness gives them problems with motivation
- The illness gives them problems with thinking clearly
- Medication side effects, such as drowsiness or shaking hands, make some tasks difficult
- The family does not let them do tasks, for example preparing the coffee, because they are worried they will do it wrong.

24.3 Why is it important to improve day to day functioning?

- Keep the person engaged in useful tasks
- Improve memory, attention and concentration
- Distract from troublesome symptoms like hallucinations and delusions.
- Improve their self-confidence
- Reduce stigma towards them
- Improve social interactions

24.4 How to improve problems with day to day functioning?

Principles

- Establish what is normal for the household. For example, check how often other family members get washed, and whether soap is used.

- Establish what is normal for the individual. For example, check whether fetching water was expected of them when they were well.
- Set realistic goals and aim for gradual improvement. The individual will need more time than usual to do the tasks
- Involve the family at every stage of the process. The aim is for them to be able to support the individual to do the task when you are not there.
- The individual will often know how to do the task, but may have problems remembering or being motivated to do it. Focus on getting the individual back into a routine.
- Ask the family members to give encouragement when any of the tasks are done
- You may notice that there are problems with hygiene and sanitation within the household that are not only related to the individuals' illness. If this is the case you could direct some of your advice, for example about the importance of washing, towards other family members too. You can also consider asking the health extension worker to visit to discuss sanitation, if they have not already done so.

1. Explain why you are doing this module

Explain to the individual and family that problems with day to day functioning are common in people with schizophrenia. Give some of the reasons why people have problems and why it is important to improve the situation.

2. Assess for problems with self care

Ask what problems the individual has with day to day functioning, using the Day to Day functioning Progress Form (Form 16). Ask the individual why they are having difficulty. Focus on the tasks where there is a problem.

3. Give advice on how to improve each self care task

For each task:

- Discuss why it is important to do the task. Say it is healthy to keep your teeth and body clean. Also it will make it easier to socialise with friends and family.
- Check what the family normally does and what is expected of the individual
- Agree a time when the individual will do the task e.g. clean teeth straight after waking

- If the individual is very disorganised you may need to talk through, or demonstrate, the stages in the task. If appropriate, ask the individual to have a go at doing the task
- To begin with the caregiver may need to watch the individual doing the task

Tasks may include:

a. **Cleaning teeth**
b. **Washing**
c. **Grooming e.g. braiding hair, cutting nails.** The family should supervise the first few attempts at cutting nails to ensure that he/she cuts it safely and neatly.
d. **Using the toilet, washing hands and menstrual hygiene.** This is a sensitive issue and you must be careful when you discuss this topic so as not to offend the individual or the family. Discuss the disposal and washing of soiled clothes.
e. **Dressing and taking care of clothes.** E.g. not wearing too few or too many clothes**.**
f. **Healthy eating habits.** Ensure the individual is being involved in mealtimes with the rest of the family. Discuss the important things to do and not do whilst eating e.g.
    - Wash hands before eating
    - Only eat with the right hand
    - Do not lick fingers
    - Do not reach across other people
    - Wash hands after eating

4. Assess for problems with household tasks

Ask what household tasks the individual finds difficult. Refer back to any problems highlighted in the needs assessment. Try to understand from the individual why they are not doing the task.

5. Give advice on how to improve household tasks

For each household task where there is a problem, go through the following steps

- Discuss what is expected of the individual
- Talk through, or demonstrate, the stages in the task
- If appropriate, ask the individual to have a go at doing the task

The household tasks might include:

- Tidying the sleeping area, and folding the blanket

- Keeping the room tidy. They should be encouraged to pick up fallen items and replace them. They should clean the floor if something has been spilt.
- Sweep the room every day.
- Cleaning and cutting vegetables
- Fetching the water
- Preparing coffee
- Lighting the fire

6. Encouragement and positive feedback

For each self-care and household task, ask the family to encourage the individual to do the task and give them positive feedback when it is done. Tell the family the individual will need more time than usual and will need breaks whilst doing the tasks.

7. Creating a daily routine

Once the individual has some ability to do the individual tasks, you should help them to get into a daily routine. The daily routine should be a **combination of work, rest, leisure, self-care and sleep**. The following is an example of a daily routine.

12:00: Wakeup, attend to toilet, wash face, brush hair

12:15: Tidy bed

12:30: Say prayers

1:00: Eat breakfast

1:15: Wash plate and glass, tidy table

1:30: Milk the cows and feed them

2:30: Fetch water

3:30: Rest or tea/coffee break

4:00: Household activities like cooking, tidying rooms

6:30: Lunch

6:45: Wash plate, glass and tidy table

7:00: Rest

8:00: Wash clothes

9:00: Weaving cloth

10:00: Tea/coffee and converse with family, friends or neighbours

11:00: Feed the cows

12:00: Household tasks like cooking

2:00: Eat dinner

2:30: Wash plate and glass and tidy table

3:00: Listening radio or talking with family members

4:00: Sleep

You can make a similar routine for the person you are working with after considering their age, sex, needs and wishes. Give the individual guidance on how to do all the tasks. Ask the caregiver to be involved and encourage the person stick to the routine as far as possible. It is usually helpful to write it down for future reference, even if the individual and family cannot read.

8. Give advice on managing money

- The family may need to help the individual to recognize the types of coins and notes.
- Family members should encourage the person to purchase things required for the family by initially giving the exact amount to be spent. Later, the person should be encouraged to pay the appropriate amount, collect the correct balance and return home with the items.

9. Make a plan

Decide which task the individual and family will try before the next session. Agree what each person's role is. If there are many tasks that are currently difficult, focus on the ones that are a priority for the individual and family e.g. getting dressed.

Follow up

Remember to review progress at the next home visits, referring to the Day to Day Functioning progress Form.

Yosef's story

Yosef and Addis tell Berhan that Yosef does not wash and sometimes doesn't dress properly. Berhan discusses with Yosef the importance of washing and dressing properly. Yosef agrees this might be stopping him making friends again. With Addis' help, Berhan breaks down the steps of washing and dressing into simple steps. They agree that Addis will prompt him to do these steps every morning, and will give him lots of praise if he does them by himself. Together they think of a daily routine that Yosef will try to follow. Berhan doesn't write it down as the family cannot read, but they agree to try and remember it.

24.5 Summary

- People with schizophrenia often have problems with day to day functioning, such as washing themselves or tidying the house
- Problems are due to the illness, the medication and low expectations of the family
- We can improve day to day functioning by breaking down tasks into small steps, asking the family to give lots of encouragement, and making a daily routine

25 Module: Improving the family environment

25.1 What problems do people with schizophrenia have with the family environment?

Problems within the family are common when a person has schizophrenia. There may be many arguments or even violence between family members. Sometimes the family are very critical of the person with schizophrenia. Or certain family members may be involved in everything that the individual does because they are worried they cannot do it alone. This means neither one has time to themselves. All these problems sometimes mean that the family becomes less good at caring for the individual. It can also be very stressful for the caregiver, who may find it difficult to cope. Looking after children can be stressful and tiring for any parent, but especially when the parent has schizophrenia. They may feel they are not able to look after the children properly.

25.2 Why do people with schizophrenia have problems with the family environment?

The family are the main carers when a person has schizophrenia. This can put a lot of stress on the family. The individual may have disruptive behaviours, which are difficult to control. They may also not be working, which puts extra strain on the family. The costs of treatment may also be a source of stress. The family may not understand why the individual cannot work and has not recovered. All these problems can mean the family environment becomes stressful and unpleasant (See Chapter 5). Parents with schizophrenia may have problems looking after their children because they have many symptoms or because of side effects of the medication (for example drowsiness).

25.3 Why is it important to improve the family environment?

When there are problems with the family environment it usually takes longer for the person with schizophrenia to recover. It is therefore important to help the family to support the individual better. Also, improving the family environment means the family has less stress and worry. It is empowering for the individual to start looking after their children again. Doing this might improve their self-esteem and make them feel happy that they are participating in family life. It is also better for the children's development if the parents give proper love and care to the children.

25.4 How can we help to improve the family environment?

Principles

Before you can start improving the family environment it is important to have a good relationship with the family (see Chapter 11). Be aware of the fact that discussing the family environment may be upsetting for the individual and family.

1. Explain why you are doing this module

Explain why it is important for the person with schizophrenia to have a good family environment.

2. Understand problems in the family environment

Discuss with the individual and family what problems there are in the family environment. For example whether there are often disagreements or conflict. You should also check the latest needs assessment.

3. Consider how well the caregiver is coping

See Chapter 15 for how to support the caregiver if they are not coping well.

4. Give advice on how to improve the family environment.

Try and cover all the following points with the family. Relate the points to the specific issues the family has raised.

a. Have appropriate expectations

Once the worst symptoms have gone, the family may expect the person with schizophrenia to get back to their usual activities straight away. The family often feels disappointed when the individual is unable to do so. The individual may inactive, unmotivated and may need to sleep more than usual. Or they may be restless and unable to concentrate on small tasks. Give the family the following advice:

- Encourage them to have realistic expectations of what the individual can do.
- Encourage them to view the individual as having a serious physical illness, which requires a long time for resting. However they can eventually recover.
- Discourage them from thinking the individual is just being lazy.

- Encourage them to compare the individual's current behavior with how it was a month or more ago. This helps the family members to see the positive changes.

b. Set limits

Tell the family that just because the individual is ill, the family does not need to do whatever they ask. Instead, help them to set limits on the unacceptable behaviors.

- Help them to decide which behaviors are intolerable (for example violence) and which are irritating but tolerable (for example, not eating in the correct way). When the intolerable behavior occurs, they must immediately let the individual know that this is not acceptable. The aim is to stop a pattern of behaviors from starting.
- Advise the family not to discuss with the individual the reasons for setting the limit or how they feel about it. Tell them to only state that the behavior is unacceptable, without explaining why.
- Advise the family that the individual should be encouraged to behave in a way that is usual for their age. For example, an adult should not have tantrums.
- Tell the family that it will be difficult at first to set limits. It may take a number of weeks. Encourage them not to give up trying.
- Tell the family that they should not ignore behaviors such as suicidal threats or violence. See Chapter 18 for how to address these situations.
- When the individual behaves well, advise the family to reward them. This can be through giving food, allowing them to do a favourite activity, or through smiling, patting and praising them. Ask the family to think about which reward is the most useful.
- When the individual does not behave well, the family should stop rewarding him or her. If possible, the individual should be taken away from the others for a short period and returned when the individual is behaving well again. For example, when the individual disturbs others by shouting, then he or she should be taken to another room and not spoken to until he stops it. However it may be difficult to do this when the individual is very unwell.

c. Reduce stress

Remind the family that the individual should not be put under a lot of stress. Use the stress-vulnerability model to help explain (see Section 2.3).

- Tell the family to try and reduce arguments and nagging behaviours (e.g. constantly commenting negatively about what the person is doing).
- Tell the family not to be very critical of the individual or hostile towards them.
- Advise family members against talking about complex and emotional topics whilst the individual is there, for example discussing problems with money.
- Advise them that being very enthusiastic or showing lots of concern can be upsetting for the individual. Ask them to try and reduce these behaviours.
- Advise them that if the individual wants to be alone the family should allow it. They do not need to be involved in everything the individual does.

d. Improve communication

- People with schizophrenia often do not reply straight away when they are spoken to. Advise the family members to avoid speaking on the individual's behalf when this occurs. Advise them to wait for the individual to respond.
- Advise the family to avoid detailed conversations, which the individual may find confusing.

e. Get the family routine back to normal

Discuss with the family the problems that come when the individual is the centre of their lives. Tell them that since schizophrenia is a long-term illness, after some time they may become too stressed to be able to look after the individual. They may also develop problems of their own. As far as possible, encourage them to start again the daily routines that they had before the illness. Advise them to think about the needs of other family members, for example children or siblings.

f. Strengthen social networks

Sometimes family members think that they should not worry other people with their problems. They may also worry about the reactions of others.

- Discuss with the family the benefits of discussing their problems with others like relatives, friends, neighbours, religious or community groups. This may help to reduce their distress. It also helps them to think of new and better solutions to their problems.
- Tell the family members that they will not be able to care for the individual for a long time unless they look after themselves as well.

g. Treat the individual with respect

Discuss the importance of respecting the individual as an adult who can make decision for themselves

5. Discuss unhelpful coping strategies

Sometimes family members do things that are not helpful or may even make the family environment worse. These include:

- The family may try to persuade the individual that their unusual ideas are not true. This may lead to arguments and angry responses from both sides.
- Even when it is clear that the individual does not make sense, family members may try to find a meaningful message in what the person is saying.
- Sometimes the family members ignore the symptoms and pretend the illness is not important. This is unhelpful when the symptoms are very bad.
- Family members may constantly supervise the individual, as they are not sure what they will do next. This makes the family member feel more in control. However, it is difficult for the family to maintain this constant supervision for a long time.
- The needs of other family members are ignored because the needs of the individual are so great. This may mean that other family members feel uncared for and problems with family relationships happen.

6. Give advice on how to improve parenting

- When the individual is well, encourage them to spend time with the children and to take on their usual parenting tasks. For example, helping the children to dress and wash.
- Discuss with the family how they can support the individual to get back to these activities. Ask the family to give encouragement when the individual does the activities.
- If you are concerned about the safety of the child, inform your supervisor (see Chapter 14 and Chapter 35).

7. Make a plan

Ask the family to consider what specific things they will try to do differently.

Follow up

Check how for progress at the next home visit. Ask what the family tried, and how easy or difficult this was. Ask the individual and family if the family environment feels any different.

> **Yosef's story**
>
> *Yosef and Addis agree that there are lots of arguments within the household. They usually argue about the fact that Yosef is still not working, even though in some ways he is better. Yosef also complains that he never has any time alone.*
>
> *Berhan helps them to discuss this together in a calm way. She suggests that the family need to have realistic expectations of Yosef and that it might take a long time for him to get back to his previous work. Addis agrees that she will try not to nag Yosef. They discuss that it might be useful for Yosef to spend more time alone during the day, at least whilst he is feeling well.*

25.5 Summary

- Sometimes there are problems with in the family when a person has schizophrenia. These include the family being too critical or over-involved in the individual, or there being lots of conflict
- Problems in the family environment worsen illness and cause stress for caregivers
- Ways to improve the family environment include: setting limits, having appropriate expectations, reducing stress, improving communication and getting the family routine and social life back to normal
- Ways to improve parenting include: when they are well encourage the individual to spend time with the children and ask the family to support the individual with this

26 Module: Taking part in community life

26.1 What problems do people with schizophrenia have taking part in community life?

It is common for people with schizophrenia to have problems doing the usual activities in the community. People may have problems with:

- Attending weddings and funerals
- Drinking coffee with friends and neighbours
- Visiting relatives
- Taking part in Edir meetings
- Participating in the practical activities of Edir
- Participating in kebele meetings
- Attending church or mosque
- Participating in religious groups, such as mahaber or lika
- Going to market
- Participating in other community organisations, such as the Women's association

26.2 Why do people with schizophrenia have problems taking part in community life?

- The illness makes them unmotivated to go outside
- The illness gives them problems with social skills, such as difficulties starting a conversation or taking turns to speak
- Medication side effects, such as drowsiness, make it difficult to get the energy to walk somewhere and socialise.
- The family does not let the individual attend community events because they are worried they will behave strangely or do something embarrassing
- Community members may have stigmatising attitudes towards people with schizophrenia. This means they may ignore the individual, not allow them to participate in activities, not respect their opinion or even shout names at them.

26.3 Why is it important to take part in community life?

- To feel part of the community
- Improve their sense of well-being
- Improve their self-confidence
- Reduce stigma towards them

- Reduced burden on the family
- Keep the person engaged in useful tasks
- Distract from distressing symptoms like hallucinations and delusions.

26.4 What can we do to help people with schizophrenia take part in community life?

Principles

- Focus on the areas where there are problems, and which are important to the individual to change
- Establish what is normal for the household and the individual
- Involve the family at every stage of the process. The aim is for them to be able to support the individual to do the activities when you are not there.

1. Explain why you are doing this module

Explain to the individual and family that problems taking part in community life are common in people with schizophrenia. Give reasons why people with schizophrenia have these problems.

2. Discuss what problems the individual has

Discuss what problems the individual has had taking part in community life

3. Identify the activities the individual wants to do

Find out which community activities the individual would like to do but is having difficulty with at the moment. You should refer back to the latest needs assessment.

4. Identify barriers and suggest ways to overcome them

You should discuss with the individual and their caregiver why they think they are having problems taking part in these community activities. Look at the section 26.2 for suggestions. The most appropriate approach for helping individuals to take part in community life will depend on the types of barriers. You should also use the problem solving approach to help you (see Section 10.5). You can suggest some or all of the following:

a. Improve social skills

- Ask the individual and family member to think about what social skills they need to do the community activities they would like to be involved in. These might include:
    - Greeting others

- Listening to others
- Asking for information
- Expressing an opinion, for example about their own treatment.
- Saying sorry when this is needed
- Eating with others in a polite way

Now discuss with the individual and caregiver which things the individual has problems with. Focus on these things.

- Ask the individual to practice these skills with you. For example,
    - Ask them to show you how they would greet a neighbour they met at church.
    - Ask them to show you how they would ask the price of somethings, for example cabbage at the market
- Give encouragement, including smiles and praise, when the individual shows they can do the skill
- Ask the individual to try the skills in a real life situation

b. Invite neighbours or relative for coffee

Suggest that the family invite neighbours or relatives to the home to drink coffee. This can be a chance to practice talking to familiar people in a familiar environment.

c. Family member accompanies the person to community activities

Suggest that a family member goes with the individual to the community activity for the first few times. This will give the individual more motivation and confidence. The family member may be worried that the individual will behave in an embarrassing way. Suggest that they start with activities that are close to the house and involve fewer people. For example it may be less stressful to go to the grain mill together, than to attend a funeral with many guests.

d. Engage with community leaders

With the help of the individual and the caregiver, identify key people within the community who may be able to help the individual to get back to usual activities. Consider:

- Religious leaders, who may be able to help the person go to church, mosque or a religious group (mahaber or lika)

- o Edir leaders, who may be able to help the person contribute to Edir and participate in the Edir activities
- o Leaders of Youth Association, Women's Association or kebele leaders, who may be able to help the person attend meetings and contribute to kebele affairs

If you have not already done so, it may be appropriate for you to meet with the leader to give them information about schizophrenia and to see how they can help. See Tasks 8, 9 and 10 in Community Engagement Chapter 31. Alternatively the caregiver may be willing to approach the leader independently.

e. Use approaches from other modules

Some of the approaches from other modules may also contribute towards helping individuals to take part in community life, for example:

- Community-awareness raising (see Chapter 31), which aims to reduce stigma and discrimination towards people with schizophrenia
- Improving adherence and improving side effects (see Chapter 20)
- Giving the individual strategies to deal with stigmatising attitudes (see Chapter 28)
- Improving the attitude of the family towards the individual (see Chapter 25)
- Dealing with distressing symptoms such as hallucinations, lack of motivation and problems with organisation (see Chapter 22)

f. Acting as a role model

The way you behave in the kebele can have a big impact on other people's attitudes. When you see the individual outside whilst not in a home visit, greet them, as you would do anyone else. Showing the community that the individual is just like other people may be one way to reduce stigma.

You can use Table 6 as a guide for which approach to use in which circumstances:

Table 6 Approaches to support individuals to take part in community life

Problem	Approach
Lack of motivation	• Family member accompanies<br>• Invite neighbours or relatives<br>• *Dealing with distressing symptoms*
Medication side effects	• *Address side effects*
Problems with holding a conversation	• Practice social skills<br>• Invite neighbours or relatives
The family does not let the individual attend community events	• *Improving the family environment*<br>• Invite neighbours or relatives
Stigma and discrimination from community	• Engage with community leaders<br>• Acting as a role model<br>• *Dealing with stigma and discrimination*<br>• *Community awareness raising*

5. Make a plan

Discuss with the individual which specific community activity to focus on first, and agree the steps to start participating again. Make sure everyone knows what their role is.

Follow up

Make sure you follow up progress on participating in community activities at the next home visit. Discuss what strategies were tried and which ones helped. Practice social skills again if you agree this may help. If relevant, make contact with community leader to facilitate engagement in community activities

Sara's story

Since she became unwell Sara has had problems visiting her sisters in the neighbouring kebele. This is something she used to enjoy a lot. Often she doesn't go because she doesn't have the energy to leave the house. She is also worried that she doesn't have anything to say. Berhan discusses with Sara and Alemu, Sara's husband, how they can change the situation. Sara suggests that she could invite her sisters to visit her first of all as this will require less energy. Then if this goes well, Alemu can try to support her to visit them. Berhan helps Sara to practice how to greet people and how to start a conversation. They all agree to try and invite the sisters within the next two weeks, which is when Berhan will visit again.

26.5 Summary

- Many people with schizophrenia have problems taking part in community life such as attending church or funerals.
- Helping individuals take part in community life is good for improving self confidence, reducing stigma, and reducing the burden on the family
- Ways to help individuals take part in community life: improve social skills, invite neighbours for coffee, family member accompanies, and engaging community leaders

27 Module: Getting back to work

27.1 What problems do people with schizophrenia have with work?

When we talk about work, it might mean different things depending on the individual and whether they are a man or a woman. Work can include:

- Farm work on the family farm
- Daily labouring on other people's farms
- Household work e.g. fetching wood, fetching water and preparing food
- Paid employment for example in a shop
- Trading at market

Many people with schizophrenia have problems with work. They may find they can work for shorter periods or only on simple tasks. Some individuals find it difficult to work at all.

27.2 Why do people with schizophrenia have problems with work?

- They are unwell and have lots of symptoms
- The illness makes it difficult to concentrate and be organised. For example, handling money at market may become difficult.
- Medication side effects, such as drowsiness and shaking hands, make it difficult to work, particularly if it involves physical labour
- The illness gives them problems with social skills
- The family does not let the individual do their usual work tasks as they are concerned they will do it wrong
- Community members may have stigmatising attitudes towards people with schizophrenia. This means it may be difficult to get paid work, for example daily labouring, within the community.
- They are restrained at home so cannot work.
- In between periods of being well and able to work, the individual has relapses, during which time it is not possible. This can make the individual seem unreliable.
- Whilst they were unwell they may have lost their farmland or cattle. This may be because they had to sell them to get money for medication or because they were unable to work. Or the land or cattle may have been given to another family member to look after.
- The illness makes them unmotivated.

27.3 Why is it important to get people with schizophrenia back to work?

- Helps to improve the economic status of the family
- Improves self-esteem and confidence
- Helps to stop the individual from thinking about their symptoms
- Reduces isolation and improves social skills
- If the person can work alone, this means the caregiver does not have to stay with them, and they can do their own usual activities
- Improves relationship with family

27.4 How can we help people with schizophrenia to get back to work?

Principles

- Focus on the areas where there are problems
- Establish what is normal for the individual
- Go slowly and steadily; they shouldn't take on too much work too soon.
- Involve the family at every stage of the process. The aim is for them to be able to support the individual to work when you are not there.

1. Explain why you are doing this module

2. Discuss what problems the individual has had with work

Discuss what problems the individual has had with work and why they have had them.

3. Identify the work the individual wants to do

Establish what was work was normally done by the individual before they became unwell. If they used to do several types of work, for example going to market, looking after the household and doing farm work, discuss with the individual and family which type of work to start with. It is usually sensible to start with the simplest type of work.

4. Consider what preparation is needed

This will depend on the type of work and the wishes of the individual and family. If the individual needs to engage with people outside the family during work, for example daily labouring, their needs may be different. It might be necessary to ensure they have good self-

care, improved social skills and the symptoms are under control. If the person plans only to work on the family farm, it might be less important to achieve these things prior to doing work.

5. Try specific tasks

- Once you have decided the type of work, ask the family to select one specific task within this. For example, if the focus is on farm work the first task selected might be looking after the cattle. Ask the family and individual to select a task that is:
    - Something the individual used to do, or is expected to do
    - Something which the individual feels ready to try
- With the family, break down the specific task into individual steps. For example, the steps involved in looking after the cattle are:
    - Untying the cattle
    - Taking them to the field
    - Watching them whilst they graze
- Ask the individual to try doing the task with a family member present. They should decide in advance which of the individual steps the family member will do, and which the individual will do. They should gradually work towards the individual doing all steps. Even if the individual makes a mistake the family should give encouragement to continue.
- Discuss that the individual will probably only be able to work for short periods at the beginning. They may need to lots of rests whilst doing work.
- Once the individual is able to do some of or the entire specific task, choose another task together and repeat steps a to c again.

6. Encourage independent working

As the individual becomes more confident and able to complete the specific tasks, the family members should encourage them to work independently.

7. Consider asking community members or an NGO for their input

In some cases you may need the help of community leaders and other community members. Any meetings with other community members should be done with the permission of the individual and family.

- In your initial meetings with community leaders you should have found out the potential for daily labouring and other paid employment opportunities within the kebele (see Task 7 in Community Engagement Chapter 31). If you found there were any opportunities, you should now discuss them with the individual and family. Follow Task 15 in Chapter 31 for how to follow up these opportunities.
- If they lost their farmland or cattle whilst unwell, you may also need to ask a community leader for input.
- Discuss with your supervisor if there is a local NGO who can offer some livelihood support or skill development.

8. Identifying barriers

You should discuss with the individual and their caregiver why they think they have had problems doing the work so far, and what potential problems might arise if they try to do the work now. Look at the Section 27.2 for some suggestions. You can also use a problem solving approach (see Section 10.5). Try to address these barriers alongside trying the specific tasks (step 4). This will increase the chances of the person being able to work.

Table 7 Approaches to support individuals to get back to work

Barrier	Approach
Unwell and lots of symptoms	• *Module: Supporting individuals to take medication*
Lacks confidence	• *Encouragement and support from you and family*
Problems with motivation	• *Module: Dealing with distressing symptoms*
Problems with organisation	• *Module: Dealing with distressing symptoms*
Medication side effects	• *Module: Supporting individuals to take medication*
Problems with holding a conversation	• *Practice social skills (Module: Taking part in community life)*
The family does not let the individual work	• *Module: Improving the family environment*
Stigma and discrimination from community	• *Engage with community leaders*<br>• *Module: Dealing with stigma and discrimination*<br>• *Community awareness raising*<br>• *Acting as a role model*
Relapse	• *Module: Taking control of your illness*
Farm land has been taken by kebele administration	• *Engage with community leaders*
Farm land has been taken by other family	• *Identify who has decision-making power within family and discuss with them the benefits of individual starting to work on or look after own land or cattle again*<br>• *Module: Improving the family environment*
Farmland lost due to poverty	• *Engage with community leaders*<br>• *Engage with NGO*

9. Difficult situations

The individual or family may ask you for financial help, for a loan or for a job. If this happens you should explain that unfortunately you are not able to provide any of these things to them or indeed to any of the people you are working with. Tell you supervisor if the individual or family continue to ask you about this, and consider discussing together.

10. Make a plan

Make sure everyone knows what steps they will take before the next home visit.

Follow up

Once you have started helping the individual to get back to work it is important that you follow this up every time you see them.

- Check what progress has been made with specific tasks.
- Check what progress has been made overall. For example, how much of the day does the individual spend doing farm work? How does this compare to when they were very unwell? How does this compare to when they were well?
- Check for any problems. In particular whether the work is too stressful or the individual feels they are not being given enough responsibility. Try to change the plan to fit better with the individual's needs.
- Check the progress with any links made with community members
- Review any barriers that are still present and try to address them using the suggestions above.

Yosef's story

Yosef no longer does any farm work. Before he became unwell he did all the necessary tasks. He says the reason he doesn't do the work is because he usually feels tired and unmotivated, and the work seems overwhelming. Yosef and his family agree Yosef is ready to start trying to work again as his symptoms are quite well controlled. They agree he will start by helping his father to thresh the wheat. Before he starts they discuss the specific steps involved, and agree that Yosef should only work for a short time initially, before having a rest.

27.5 Summary

- Many people with schizophrenia have problems with work. This may be due to the illness, medication side effects and stigma
- Getting back to work is important as it helps to improve confidence and social skills, bring money into the family and reduce stigma
- To help the individual get back to work: decide what preparation is needed then try specific tasks broken down into steps.
- Consider working with community leaders to help find work within the community

28 Module: Dealing with stigma and discrimination

28.1 What problems do people with schizophrenia have with stigma and discrimination?

In Chapter 6 you learnt about the problems that people with schizophrenia have with stigma and discrimination.

28.2 Why do people with schizophrenia have problems with stigma and discrimination?

Stigma can be found in family members, community members and health workers. A person may also start to believe the stigmatising things that people say – that is called self-stigma.

28.3 Why is it important to reduce experiences of stigma and discrimination?

Stigma and discrimination can mean that individuals find it more difficult to return to the activities that they used to do when they were well. People with schizophrenia may continue to be discriminated against even though they have recovered. It can be upsetting to feel separate and different from family, neighbours and community. People with schizophrenia often feel that stigma and discrimination is even more distressing than the symptoms of the illness. It is therefore important to reduce stigma and discrimination. Individuals may get back to usual activities faster if these problems are addressed.

28.4 How can we reduce experiences of stigma and discrimination?

1. Explain what stigma and discrimination is

Explain what you mean by stigma and discrimination

2. Explain why you are doing this module

Explain why it is important to reduce experiences of stigma and discrimination.

3. Discuss experiences of negative comments and discrimination

First of all, discuss what experiences of stigma and discrimination the individual has had. Discuss what happened and how it made them feel about themselves. If the individual has trouble thinking of any experiences, you can you use Section 6.2 to prompt them. Remember this might be upsetting for the individual and family.

4. Encourage the individual to think about themselves in a positive way

Discuss with the individual that even if we cannot control the way other people see them, they should still see themselves as a valuable person. Tell the individual that the illness is not the only important thing about them, and that they can recover and have a meaningful life.

5. Discuss how to deal with negative comments and discrimination

a. Discuss how the individual dealt with the situation at the time. For example, what they did when someone called them an insulting name. Discuss whether the way they responded was useful or not.
b. Discuss other ways the individual could respond. For example, it may be useful to practice explaining the illness to others. You could suggest that the individual tries saying, "I have an illness like other illnesses. I am taking the medication, which makes me better " or "This illness could have happened to anyone."

6. Discuss which ways of responding would not be useful.

For example, getting into a fight.

7. Discuss the caregiver's experiences stigma and discrimination.

You can repeat these steps with the caregiver.

8. Discuss what other approaches may reduce stigma

There are many parts of CBR that help to reduce stigma and discrimination. These include:

a. Give information about schizophrenia to the person with schizophrenia and their family (see Chapter 16). In particular, focus on the possibility of positive outcomes.
b. Awareness-raising amongst community members and community leaders (see Chapter 31). Once people have more information they are less likely to have negative attitudes towards people with schizophrenia.
c. Supporting individuals to improve self-care, to get back to their usual activities within the community and to get back to farm work or employment (see Chapter 26 and 27). When people in the community see the individual doing usual activities again, this will help to reduce their ignorance and prejudice towards people with schizophrenia.

d. Supporting access to treatment and support taking medications will hopefully improve symptoms (see Chapters 17 and 20). This makes it more likely the family and community will treat the individual as normal, and also shows people that schizophrenia is treatable.

e. If the individual or their family is worried that home visits by you may bring negative attention to the household, suggest you hold the sessions elsewhere, for example at the health post.

f. Set up a Family Support Group so that the person and their family can share their experiences, including ways to deal with stigma. The groups can also be a way to improve self-esteem (see Chapter 32).

g. Encourage the family to accept the person's illness and treat the individual in a dignified way (see Chapters 19 and 25)

h. Treat the individual with respect and dignity yourself (see Chapter 11).

9. Make a plan

Agree what actions the individual and family will take before the next session

Follow up

At the next session check if there have been any new experiences of stigma or discrimination and if so how the individual dealt with it. Ask whether the strategies were useful. Discuss how to change the strategy for next time it happens.

Sara's story

Sometimes children in the kebele shout at Sara that she is mad. They laugh at her and sometimes throw stones. Sara finds this very upsetting and it makes her want to stay at home all the time. When it happens usually she ignores the children, which is quite helpful. However, she tells Berhan that she would feel more confident to go outside if she had some way of responding. Sara and Berhan practice helpful things that Sara can say in a calm way to the children next time. They agree that it is not helpful to throw stones back. Alemu agrees that it is also not helpful when he calls Sara names. Sara and Alemu agree to try these ideas before they next see Berhan in one month's time. Berhan tells Sara that she is doing awareness-raising in the kebele, which hopefully will improve the attitudes of community members, including the children.

28.5 Summary

- Stigma and discrimination is a common experience for people with schizophrenia
- It is important to reduce stigma because it is a barrier to returning to usual activities
- Ways to reduce stigma include: helping the family and community to understand schizophrenia better, supporting individuals to get back to usual activities and giving them strategies to deal with negative comments

29 Module: Improving literacy

29.1 What problems do people with schizophrenia have with literacy?

Like many people in rural Ethiopia, often people with schizophrenia are not able to read and write. This is more common in women.

29.2 Why do people with schizophrenia have problems with literacy?

People with schizophrenia may have more problems with literacy than other people if they had to leave school early due to the illness. People with schizophrenia may also find it more difficult to access the adult literacy training that is available in every kebele. This may be because of:

- Being too unwell to take part
- Having problems with motivation or problems thinking clearly
- Stigma and discrimination meaning people with schizophrenia are excluded simply because of their illness.

29.3 Why is it important to improve literacy?

Improving literacy in people with schizophrenia may have the following benefits:

- Improve self-esteem and confidence
- Provides a meaningful activity, which may distract from symptoms and side effects
- Helps to get back to usual activities for which it helps to read and write, for example participating in kebele or edir activities, or conducting business

29.4 How can we help to improve literacy in people with schizophrenia?

Principles

It is not your role to teach the individual to read and write. Instead you should help them to access existing facilities using the following steps. Before you undertake this module, confirm that there is adult literacy training or a school (if the individual is school age) in the kebele that is accepting students. Discuss with the co-ordinator or headteacher whether there is any reason why an eligible person with schizophrenia could not attend, if they wished to. See Task 15 in Community Engagement chapter. If there is a facility available, continue the following steps.

1. Explain why you are doing this module

Explain why it may be important for some people with schizophrenia to improve literacy

2. **Find out the level of literacy of the individual**

- How many years did they go to school for?
- How long ago were they at school?
- Can they write their name?
- Can they read shop signs?

3. **Confirm the individual is interested**

Confirm that they are interested in learning to read and write.

4. **Arrange attendance at facility**

Ask the individual and family member to approach the literacy group or school to arrange attendance. If the individual and family are not willing or able to make the initial link, arrange a meeting with the co-ordinator or headteacher yourself. Discuss the potential benefits for the group/ school and for the individual.

5. **Help the individual to attend**

Facilitate the individual to attend the group/school if this has been arranged. For example, discuss with the family who will take them to the facility. It may be possible for a family member to sit in the group initially.

Follow up

Follow up progress each time you see the individual. Ask the following questions:

- Has the individual attended the group/school?
- Were there any problems? If so try to use a problem solving approach to address them.
- Is the individual noticing any benefits?

29.5 Summary

- Many people in rural Ethiopia are unable to read and write, but this may be more common in people with schizophrenia
- Learning to read and write may help with confidence, and getting back to work or community activities
- You should not teach the individual to read and write but you can help them to access existing services

30 Module: Taking control of your health

30.1 What problems do people with schizophrenia have with relapse?

Most people with schizophrenia have an 'up and down' course of the illness. This means sometimes they feel well and have no symptoms, while at other times they experience a return of symptoms. When symptoms return in a way that is problematic and distressing, this is known as a relapse.

30.2 Why do people with schizophrenia have relapses?

Different people experience relapses due to particular reasons and the time between relapses also varies a lots. Most often relapses develop gradually over a period of a few weeks. But some people experience a relapse very quickly, in a matter of days. Not taking medication makes it more likely that a relapse will happen. Some stressful situations can trigger a relapse, including:

- Illness or death in the family
- Change in daily routine or living arrangement
- Argument or relationship problem with family member or neighbour
- New responsibilities, for example due to family illness or relatives visiting
- Physical illness
- Khat or alcohol use

What are the early warning signs of relapse?

During the time before the relapse, there are changes in the person with schizophrenia that are called *'early warning signs'*. These warning signs are often unique to each person. The person with schizophrenia and family members can often identify them since they have seen this happen a few times. These early signs of a relapse are important to recognize and respond to, since this can be a time when something can be done to stop the person from suffering the serious consequences of a relapse. The most common early warning signs are:

- **Tension and nervousness**

Individuals may report feeling anxious, nervous or tense about small things that they would not usually worry about. People feel worried, walk around the house excessively, and are unable to rest by sitting in one place for long.

- **Sleep disturbances**

Many people with schizophrenia experience changes in their sleeping habits. They either

have difficulty in sleeping or start sleeping more than normal.

- **Feeling sad and angry**

Individuals may feel sad all the time. This can lead the person feeling hopeless and having suicidal ideas. Family member may notice that the person is talking less, hardly smiles, looks worried and is often tearful.

- **Becoming isolated from others**

Many people will become withdrawn and less interested in talking to people. This is often noticed first by family members as the person becomes quieter, does not meet guests, and avoids going out of the house. There can be many reasons for the social isolation. Some people feel tense and anxious if they have to talk to someone, while others can start feeling suspicious of people and decide the best way to deal with it is by reducing social interactions.

- **Difficulty in concentration:**

Individuals may find it more difficult to concentrate and pay attention to something for a period of time. This leads to difficulty in following a conversation, cooking a meal, or remembering to get things from a shop.

- **Reducing or stopping medicines**

Sometimes the person with schizophrenia does not realize that they are going to have a relapse. Because of this they may decide to reduce or stop the medication. They may also refuse to go to the health centre or refuse to see you.

- **Change in appetite**

Before a relapse people often change their eating habits. Usually there is a loss of appetite. This can sometimes lead to a loss of weight and to a general sense of tiredness and lack of energy. Less commonly, people may start wanting a lot of food several times a day.

- **Unique signs of relapse**

Quite often, family members will be able to describe particular signs of a relapse that are unique to the individual and tend to occur only in such situations.

30.3 Why is it important to prevent relapse?

During a relapse, most people are too unwell to do farm work, housework or other usual activities. Even after the symptoms stop, it can take a few weeks or months for the person to return to the previous level of functioning. Some people may even feel that they want to commit suicide during this time due to constantly hearing voice telling them negative things or

because they have developed depression. Others can become very suspicious and frightened. This is also the time when some people become irritated and easily angry; rarely, there can be a risk of harm from the person to others in the family or community. The family may feel they have no choice but to tie up the person with schizophrenia. After a relapse the individual may also have a sense of failure ('This happened in spite of trying so hard to stay well'). They may also feel more stigma and discrimination from their family, neighbours and community. Having a serious relapse is a setback for everyone and preventing this from happening is one of the most important goals of CBR.

30.4 How can we prevent relapse?

Principles

Relapse prevention involves helping the person with schizophrenia and the family to recognize the early signs of a relapse and putting in place a plan to deal with the situation. It is a joint effort involving you, the person with schizophrenia and the family. The focus should be on helping the individual to take control of their own health. The aims of relapse prevention are:

- To prevent a relapse from happening. Unfortunately this is not always possible.
- To minimize the severity of the relapse so that the time to recovery is shortened.

To prevent relapse we should do two main things:

- Firstly, we should support individuals to take medication.
- Secondly, we should try and reduce life stress by helping individuals to cope better with their problems.

Before undertaking this module, decide who to involve. The person with schizophrenia must always be involved, as well as the primary caregiver and any other key family members who the person trusts and is comfortable with. The group should be between 3 and 5 people and will usually involve parents, spouses and in some cases adult children or more distant relatives. Gather the group together to carry out the following steps:

1. Explain why you are doing this module

Give information about what relapse is, what problems it causes and why it is important to prevent it.

2. Discuss the problems of relapse

Discuss what happened during the most recent relapse, for example which symptoms were most prominent and what usual tasks the person could no longer do. All members of the group should be encouraged to join the discussion.

3. Discuss the early warning signs

Explain what early warning signs are and ask the group whether they usually notice any. Use the Early Warning signs Checklist (Form 17) to make sure you haven't missed any. Agree on 3 to 5 important signs for the individual.

4. Discuss stressful triggers

Explain that relapse can be caused by certain stressful situations. Ask the group whether they can remember any which came before the person's relapse. Explain that it is useful to think about these so they can be avoided in the future, where possible.

5. Discuss the relapse management plan

Explain that the purpose of the plan is to decide in advance how you will all respond to early warning signs. Having a plan may help us to prevent a serious relapse, or help it to resolve quicker. The plan should be made jointly with the group members. In particular the wishes of the person with schizophrenia should be sought and included in the plan. They should feel they have some control over what happens to them during the relapse. Record the plan on the Relapse Management Form (Form 18). Each member should be clear on what their role is. If appropriate, give a copy of the plan to each member of the group. The detailed parts of the plan will be different for each individual. However for most families the following elements should be included.

a. Family meeting

If early warning signs are noticed by the person with schizophrenia or family members, a family meeting should be held. Concerns should be discussed openly and a plan is agreed on. This process should help everyone, including the person with schizophrenia, to participate as equals in trying to improve the situation. This process also helps the person with schizophrenia and family take on responsibility for managing the illness and improve their sense of control.

b. Review whether the individual is taking medication regularly

Not taking medication is often an important factor in a relapse. Find out the person's reasons for stopping the medicine. Take the steps in the adherence support chapter to ensure the individual starts the medication again as soon as possible.

c. Clinical review

Arrange for a review at the health centre as soon as possible. Inform the person with schizophrenia and family when the appointment is. Check if there are any major barriers to attending the health centre and try to resolve them. If you feel they are unlikely to attend, you might need to accompany the person to the health centre. After the review, check if everyone has understood the advice from the nurse.

d. Reduce stress

Identify any obvious stressful situation that the person has experienced recently. If possible try to remove or reduce the stress. Suggest stress management activities, particularly those that have been useful in the past (see Chapter 23).

e. Reduce alcohol or khat

Using alcohol or khat may make the relapse worse**.** You and the family should help the person to reduce or stop using alcohol or khat. For example, by removing any bottles from the house, providing less money to buy it and by reinforcing any steps taken by him to reduce alcohol use.

f. Activate social and practical support

Identify relatives, friends and community leaders who may be able to provide social and practical support. In particular, the caregiver may need more support during a relapse. For example, neighbours may be able to help with the farm work whilst the person is unwell.

g. Monitor the situation

Your role is to closely monitor the situation. At each visit check whether the early warning signs have improved.

6. Make the plan

Ensure each member is clear on what his or her role in the plan is.

Follow up

It is a good idea to review the plan every 6 months, to ensure that everyone involved remembers the issues clearly and any updates can be made.

30.5 Summary

- When symptoms return in a way that is problematic and distressing, this is known as a relapse. Relapses are a common part of the illness
- Relapses may be triggered by stressful events
- You should help the family to develop recognise the early warning signs and know the likely stressful triggers
- You should help the family to make a relapse prevention plan which usually includes: family meeting, check medication, review at the health centre and reduce stress

31 Community engagement

31.1 Why is it important to do community engagement work?

The community can have a powerful impact- both positive and negative- on the experiences of the person with schizophrenia. CBR aims to increase the positive influences of the community, and reduce the negative influences. There are many community resources in every kebele (see Chapter 8). It is your role to ensure that the person can do the same community activities as other people of their age and gender. The community also has an important role in making sure the positive changes made during CBR are continued after you have left.

31.2 What community engagement work do we do?

Phase I

During Phase I you should do the following tasks in each kebele where you are working. You should do these tasks at the same time as you are doing the home visits to each individual you work with. You don't need to do these tasks in the order set out below; lots of the tasks will overlap. Remember when you talk to community leaders or other community members you should not reveal the name or problems of the individual you are caring for, unless you have got their permission to do so.

Task 1: Meet with health extension worker/s (HEW)

How?

- Get the HEW contact details from the health centre and arrange a meeting. You may need to spend between 2 hours and a whole day with the HEW.
- Check whether the HEW is aware of the people with schizophrenia to whom you have been allocated. If they are, ask if they are willing to show you where they live. Ask what they know about them and what problems they are aware of.
- Ask them who the important community leaders are (see Task 2) and if the HEW would be willing to make initial introductions
- Ask what the important community resources are (see Task 3)
- Explain what your role will be in terms of home visits and community engagement
- Explain how you would like them to be involved: support the messages you will give about reducing stigma and discrimination and the importance of accessing treatment; do their

usual home visit and offer the care they usually provide to people with schizophrenia e.g. family planning.

- Give your contact details in case they have any questions

Task 2: Identify important community leaders e.g. kebele leader/s, Edir leaders, religious leaders, traditional healers, women's association leader.

How?

- First ask the HEW to help you. If they do not have the information use: Health Centre of District Health Bureau (DHB) records; church/mosque; other community leaders
- Get the names/address/telephone number

Task 3: Identify key community resources e.g. churches/mosque, Edir, adult literacy group, schools, Mahaber/tsewa/lika groups, informal social networks, women/youth associations, NGOs

How?

- Ask the HEWs, kebele leader and community leaders
- If they do not have the information use: Health Centre or DHB records and church/mosque

Task 4: Ascertain what community work relating to mental illness has already taken place or is planned e.g. community conversations, engagement/training of traditional headers

How?

- Ask the HEWs
- This will help you to plan meetings with community leaders (Task 7) and community awareness raising (Task 6)

Task 5: Meetings with important community leaders

How?

- Ask the HEWs to introduce you to community leaders if possible
- Meet with and introduce yourself to 5-10 important community leaders from across all sectors.
- Try to speak to these community leaders at a meeting or event that is already planned, for example a kebele meeting. This will make it easier to reach them. If that is not possible,

invite these community leaders to a meeting, either in one group or small groups depending on the local circumstances. Discuss with your supervisor.

- The meeting may take up to 2 hours, but could be much less.
- At the meeting, cover the following:
    - Purpose of CBR programme (briefly describe severe mental illness; the possibly of recovering; importance of medication; the need for help with rehabilitation too; get more productive community member at the end, benefits whole community),
    - Authorisation given to CBR programme (links to district health bureau and Addis Ababa University)
    - Give outline of CBR activities, including home visits and types of modules
    - Give outline of community engagement activities (community awareness raising events, further individual meetings with community leaders according to needs of individuals)
    - Give outline of how you want the community leaders to be involved (help arrange community awareness-raising events; general support e.g. encouraging individual to keep using medication; encourage inclusion in Edir tasks for the person with schizophrenia; encouraging inclusion in kebele meetings; making sure people are not excluded from church)
    - Give outline of how you want general community members to be involved (general support; encourage general social inclusion)
    - Ask what is the best place for a community awareness-raising event
    - Ask about community resources (See Task 3)
    - Gather information on potential paid employment in the kebele e.g. farm labouring (see Task 7)
    - Arrange a subsequent meeting if the community leader/s would like more time to discuss

Task 6: Community awareness-raising event/s

How?

- You should hold the event at a community meeting that is already planned. Utilise the place suggested by the community leaders e.g. kebele or Edir meeting
- Pre-arrange the date, start time and the amount of time you have to speak. The meeting may take 30 minutes to 2 hours depending on where it is held and the topics you cover

- You may wish to arrange 2 or 3 meetings to give you time to cover all the topics. This is a suggestion for how to divide up the topics.
- Meeting 1 topics
    - Introduce yourself and explain that you are working in the kebele to try and support people with schizophrenia
    - Describe how schizophrenia is a mind disease that can improve with medication
    - People develop schizophrenia due to stressful events that happen in life.
- Meeting 2 topics
    - Explain that with time and support people with schizophrenia can get back to usual activities, such as work and socialising
    - People with schizophrenia can get medication at the health centre. This will make them better.
- Meeting 3 topics
    - To get better people with schizophrenia need the support of people in the community
    - It is important not to treat people with schizophrenia differently, for example with disrespect, calling them names, or ignoring them just because they have this illness.
    - This kind of behaviour will stop them from getting back to their usual activities
    - It is also important not to beat people with schizophrenia. This will not help them get better.
    - Getting medication will reduce the need for individuals to be chained. We should avoid chaining if possible, and instead help people with schizophrenia get treatment.
- Explain the information simply and clearly
- Listen carefully to the responses from those attending. Be respectful of any differences in beliefs or differing opinions.
- Thank attendees for their time.

Task 7: Identify potential employment opportunities in the kebele

How?

- Identify potential paid employment opportunities e.g. farm labouring by consulting: community leaders, the person with schizophrenia and HEWs. This may be on a one-off or ongoing basis.
- Arrange a meeting with the potential employer

- Discuss potential benefits for the employer, community, individual and caregiver of offering paid employment
- Address any concerns the potential employer has

<u>**Phases II and III**</u>

In Phases II and III you may or may not need to conduct the following activities depending on:

- Needs of individual and caregivers. It is indicated in the modules when a Community Engagement task is likely to be useful.
- Available community resources, which you identified in Task 3.
- What the person or their family is willing or able to do themselves (therefore not requiring your input).
- Who the individual and family are happy for you to talk to in the community
- You should always discuss with your supervisor which ones to undertake.

Task 8: Individual meetings with Kebele leaders

In what circumstances?

- If the individual is not receiving free medication but may be eligible AND/OR
- If the individual and/or caregiver is not engaged in kebele activities but would like to be, and family have been unsuccessful in getting involved without your help

How?

- Arrange meeting with Kebele leader
- Discuss the possibility of letter supporting free medication AND/OR
- Discuss the fact that the individual or caregiver finds it difficult to participate in kebele activities
- Discuss the benefits of them participating e.g. contribute to development of kebele
- Discuss how to facilitate their increased participation

Task 9: Individual meetings with Edir leaders

In what circumstances?

- If the individual or caregiver is not engaged in Edir activities but would like to be, and family have been unsuccessful in getting involved without your help AND/OR

- The health extension worker or community leaders suggest that the Edir group could give financial or practical support to the individual and family

How?

- Arrange meeting with Edir leader
- Discuss the fact that the individual or caregiver finds it difficult to participate in Edir activities
- Discuss the benefits of them participating e.g contribute to Edir work, reduce work of others.
- Discuss how to help them participate more AND/OR
- Discuss that in a crisis situation the individual may come to Edir for financial or practical support

Task 10: Individual meetings with religious leader

In what circumstances?

- The individual, caregiver or HEW identifies religious leader as important source of support AND/OR
- The individual has had difficulties attending church or religious groups, and family have been unsuccessful in getting involved without your help AND/OR
- The individual, caregiver or HEW identifies priest as currently giving incorrect or conflicting messages about treatment, including medication

How?

- Arrange meeting with religious leader
- Discuss how the religious leader can support the individual and caregiver e.g. reinforce importance of accessing care, not chaining unless absolutely necessary AND/OR
- Give information about the importance of taking medication for recovery. Be respectful and do not disagree with their beliefs AND/OR
- Discuss the fact that the individual or caregiver finds it difficult to participate in church/mosque/religious groups
- Discuss the benefits of them participating
- Discuss next steps for involving them, if possible e.g. priest is welcoming to individual; priest makes adaptations or special arrangements to encourage individual to attend.

Task 11 Individual meetings with traditional healer / holy water attendant

In what circumstances?

The individual, caregiver or HEW identifies traditional healer or holy water attendant as important source of reinforcement or currently gives incorrect messages about treatment

How?

- Arrange meeting with traditional healer or holy water priest or attendant
- Discuss how the traditional healer or holy water attendant can support the individual and caregiver e.g. reinforce importance of taking medication whilst at holy water
- Be respectful and do not disagree with their beliefs

Task 12 Individual meetings with literacy group leader or headteacher

In what circumstances?

If the individual or caregiver is not engaged in literacy group or school (and is school age) but would like to be, and family have been unsuccessful in arranging this without your help.

How?

- Arrange meeting with literacy group leader
- Discuss the fact that the individual or caregiver would like to participate in the literacy group
- Discuss the benefits of them participating
- Discuss next steps for involving them, if possible

Task 13 Demonstrate progress of individuals to community leaders/ wider community

In what circumstances?

- Person with schizophrenia who is recovered and has good functioning is available and willing to speak to general community AND
- There is a suitable place in a community setting where this individual can speak AND
- You and your supervisor assess that the community has a good level of awareness and speaking to community members is unlikely to bring additional stigma and discrimination to the individual.

How?

- Work with supervisor to identify person with schizophrenia who is suitable for this task, either through RISE or the Butajira study
- Discuss expectations with the individual e.g. any fears about stigma

- Take consent from the individual
- Arrange with community leader for individual to speak at public meeting e.g. Family Support Group meeting, kebele meeting, Edir meeting.
- Ensure the individual receives compensation for their time, transport etc. Discuss with your supervisor

Task 14 Community awareness raising consolidation

In what circumstances?

Community leaders are willing to receive further community awareness raising activities

How?

Repeat Task 6 in the same or different community meetings

Task 15 Facilitate employment opportunities in the kebele

- Arrange joint meeting with the individual, caregiver and employer previously identified as able to offer employment
- If the employment goes ahead, keep in regular contact with the employer to discuss progress and concerns.

31.3 How do we deal with difficult situations?

When meeting with community and religious leaders you may find they have different beliefs about schizophrenia, for example that it is caused by spirit possession or evil eye, or that holy water will not work if the individual takes medication. You should not tell them their beliefs are wrong. You may find it useful to say there may be many different reasons why someone becomes unwell with schizophrenia, and often we don't know the cause. Also remember that you are not suggesting that people should stop going to holy water, unless they are being harmed there. You should be working towards the individual using medication and holy water at the same time.

Occasionally, community leaders may not be interested to meet with you. If this happens you should work on building your relationship gradually, instead of insisting that they work with you. You could try asking others for help building the relationship, such as the HEW, or another community leader who is more interested in CBR. If you are having lots of problems with community engagement work you should discuss this with your supervisor and make a plan together for how to improve the situation.

31.4 How do we document the community engagement work?

For each kebele you are working in you will have a separate Kebele Logbook (see Form 10). In the Logbook you will record information all the community engagement work you undertake.

31.5 How do we monitor the progress of the community engagement work?

You will discuss at the community engagement work in each kebele at your Face-to-Face Discussions with your supervisor, and also at the Group Supervision sessions.

31.6 Summary

- It is important to do community engagement work to try to increase the positive influences of the community on the individual, and to reduce the negative influences.
- In Phase I you should: meet with the health extension worker, identify important community leaders and resources, find out what mental health awareness raising has already taken place, meet with the community leaders, do a community awareness-raising event, and find out about potential employment opportunities.
- In Phases II and III you may or may not need to do the following steps, depending on the needs of the individual: individual meetings with the kebele leader, religious leader, Edir leader, Traditional healer and holy water priest or literacy group leader; demonstrate the progress of individuals to the community; and facilitate employment opportunities
- You should document the community engagement work in the Kebele Logbook
- Your supervisor will monitor the progress of community engagement work at your supervision meetings

32 Family support groups

32.1 What are family support groups?

Family support groups are where the caregivers of people with schizophrenia who live in the same kebele meet regularly. Sometimes people with schizophrenia themselves are also part of the groups. The aim is to share experiences and information and provide one another with support. The type of support is usually emotional support, but can sometimes be practical.

32.2 Why are family support groups important?

Caregivers of people with schizophrenia are often isolated from other people in the community. They may feel alone with their problems and that no one understands what they are going through. Many caregivers, and sometimes also people with schizophrenia, find it useful to discuss their experiences with other people in the same situation. This can:

- Be empowering as it helps caregivers and people with schizophrenia feel they are able to help themselves
- Reduce feelings of isolation by seeing other people in the same position
- Help to work through problems, such as difficulties returning to farm work, by sharing ideas
- Reduce stress by talking about problems
- Help to give ideas about how to deal with stigma and discrimination
- Improve confidence and self-esteem
- Improve social skills by having a chance to meet with others in a safe setting

Family support groups may also continue after you have stopped doing CBR in the kebele. This may help the positive effects of CBR to continue after you have left.

32.3 How to we set up family support groups?

Who can participate in the family support groups?

There should usually be one family support group in each kebele. The caregivers of all the individuals you are working with and any other key family member should be invited to join. In addition, you will be asked to invite the caregivers of any other people with schizophrenia in the kebele, but who are not participating in CBR. Your supervisor will give you information about these individuals. You will not be asked to provide any other components of CBR (for example, home visits) to these additional individuals. Family support groups may vary a lot in size. In some kebeles they may be very small, for example 3 members. Family members are more likely

to benefit if the group is not too big e.g. more than 10 people (althought you can be flexible depending on the needs of the families). In some kebeles there may be a network or group of caregivers already. If so you should try to build on this rather than starting a new group.

People with schizophrenia who are unwell or very disabled will usually find it difficult to participate in the group. However some individuals may find it useful to be involved, especially when they are getting better. Discuss with your supervisor, the family and the individual, if possible, about whether they should be involved. If they are too unwell at the beginning, this should be reviewed as time goes on.

When do we start the family support groups?

You will invite caregivers to participate in the family support group around the time that the families you are working with are moving from Phase I to Phase II. This will usually be about 2 months after you start working in the kebele, but this may vary. The reason for starting around Phase II is that you are more likely to have a good relationship with the family by then, and you should have addressed the most important problems. However you should invite all families to participate at the same time, even if some of them haven't reached Phase II yet. As well as inviting the families you are already working with, you should invite the other people with schizophrenia living in the kebele at around this time.

How do we invite families to participate?

You could say something similar to this to invite individuals and families to join:

''We are planning to start a family support group in your kebele which will allow you to meet with people having similar problems as you are experiencing. This will give you opportunity to express your difficulties, gain support by sharing personal experiences and will help to learn different ways of tackling your problems. We will not be able to provide any loans, but we still hope the group will be helpful. Will you be interested to be a part of the group?''

32.4 How do we organise the family support groups?

Where will the family support groups be held?

The family support group should take place within the kebele, usually in the health post. They could also take place in one of the families' homes, if this is suggested by members of the group. The location should be easily accessible on foot for all the members.

How often will the family support groups meet?

How often the group meets should be decided together with the group members at one of the first meetings. It should usually be about every month. Once decided, the group members should agree to try and attend all the meetings.

When will the meetings be held?

When the group meets should be decided together with the group. It is usually helpful to meet at the same time each month, for example at early on the 1st Saturday each month.

How long will the family support group meetings last?

The meetings might last 60 to 90 minutes, but this should be decided by the group members.

Will we provide coffee or tea at the meetings?

You will not be given any money to buy coffee or tea for the group. However the group may decide to each contribute a small amount each month to buy coffee or tea.

How do we record the details of the Family Support Group?

You should record the details of each family support group on the Family Support Group Details Form (Form 19).

32.5 What do we do in the family support group meetings?

Who leads the meetings?

In the beginning you should be responsible for leading the meeting. After several meetings have taken place you should try to identify a group leader who can take over after you have left. The group should decide together who the leader will be. Different group members can be the leader at different time.

What is the structure of the meetings?

Whoever is leading the group- you or a group member- is responsible for keeping the structure of the meetings. Meetings should normally follow these steps:

- Welcome all members, and introduce new members to the rest of the group
- Remind the group what topic you will discuss at this meeting
- Asking group members to share their experiences, concerns and ideas on the topic

- At the end summarising the main points which were raised in the discussion
- Agree which topic you will discuss at the next meeting and confirm the time, date and place of the meeting

Very small groups may be more informal and not necessarily follow these steps.

What do we discuss in the meetings?

The group members should decide what you discuss in the meetings, but you can give suggestions. It is usually a good idea to keep to one or two topics at each meeting. Decide together which topic you will discuss next week. Topics might include:

- Sharing problems with taking medication and how to overcome them.
- Sharing experiences of negative comments or other types of discrimination from community members, and how to deal with these situations
- Discussing how to improve participation in community life. Caregivers or people with schizophrenia from different families may agree to go together to social events

What should the basic rules of the meetings be?

At one of the first meetings you should agree on the basic rules, using the ideas from the group. These should normally include:

- Everything discussed in the group should be confidential
- Everyone should listen to the other group members and share their own experiences
- No one should make judgements or criticise the other members
- Everyone must respect the other member's situation. What is right for one person does not have to be right for the others.

How do we document the meetings?

The group leader should record who attends and what was discussed on the Family Support Group Meeting Form (Form 20).

What should we do if the group asks for a loan or equipment?

The group members may ask you for financial help, for a loan, for stationary or other equipment or for training. If this happens you should encourage the group to think of how they can use their own strengths, skills and resources. Explain that unfortunately you are not able to provide

any of these things to them or indeed to any of the groups you are working with. Tell your supervisor if the group continue to ask you about this, and consider discussing together.

32.6 How do we make sure the family support groups carry on after we have left?

From the beginning of the support group meetings you should be thinking about how to make sure they carry on after you have left the kebele. Here are some ways to do this:

- Make sure the group discusses topics that are important to the members. If they are benefitting from the group it is more likely to continue.
- Tell the group that you will support them in the beginning but that you hope they will run it by themselves later on, once they are ready.
- Find a group leader from within the group after the first few sessions.
- Be prepared for the group to take a long time to get started and to run by itself.

32.7 Summary

- Family support groups are where caregivers of individuals, and sometimes people with schizophrenia themselves, provide support to one another
- They are empowering for the participants, and reduce feelings of stress and isolation
- The groups should have 5-10 members and should meet about every 15 days.
- You should lead the group initially then later a group member should lead. You should try to ensure the group carries on after you have left
- Discussion topics can include: sharing problems, sharing experiences, improving participation in community life

RISE

SECTION C: Know how you will be supported to deliver CBR

33 CBR worker wellbeing

33.1 What problems with wellbeing might CBR field workers have?

Working with people with mental illness is often rewarding but you may sometimes find it stressful. Stress isn't always bad, but it can cause problems. These problems include:

- Worrying all the time
- Sleeping badly
- Being irritable
- Drinking too much alcohol or chewing *khat*
- Not wanting to be with people
- Physical health problems (high blood pressure, peptic ulcer disease)
- Conflicts with your work colleagues over small things
- Problems with relationships at home
- Developing depression or an anxiety disorder

33.2 Why might CBR field workers have problems with wellbeing?

Here are some of the reasons why you might have problems with your wellbeing:

- You feel you have too much to do
- You are worried about your personal safety.
- You feel there are high expectations from others or the individuals aren't making enough progress
- You have to make difficult decisions
- You don't have enough support
- Working with people with many problems, you may take on their worries as your own

33.3 Why is it important for CBR workers to look after themselves?

It is very important for you to look after yourself. It will be difficult to help the people you are working with if you are not looking after yourself and feeling well. You might feel that admitting you feel stressed or unhappy is a sign of weakness or lack of commitment to your work. This is not true.

33.4 How can you maintain your wellbeing?

1. Reducing stress

Here are some ideas to make sure stress doesn't cause problems for you:

- Don't work all the time – in your rest times, meet up with family and friends, do things you enjoy and try to relax
- Eat adequately
- Try to maintain a routine
- Discuss work problems with another person, for example your spouse or a friend
- Don't be afraid to ask for more support.
- Try the relaxation techniques in Chapter 23.

2. Discuss your work

You should have lots of opportunity to discuss how your work is going, and any problems you are facing (see Chapter 34). These include:

- Meeting with your supervisor once every two weeks
- Monthly group supervision with other CBR workers
- You can call your supervisor at any time if you are facing difficulties.

Remember that it might take a long time to see positive changes in the individuals you are working with. Try to stay positive and think about the small changes which have happened since you started.

3. Seek help

There are two situations where you should look for help STRAIGHT AWAY:

- **Suicidal feelings.** If you feel so hopeless that you are thinking that life is not worth living, or even thinking about ending your life it is very important to discuss these feelings with someone you trust.
- **Problems with alcohol or khat.** If you are concerned that you have a problem with alcohol or khat or your friends or relatives tell you they are concerned, you should try to get help.

To get help you can also talk to your supervisor or go to the health centre.

4. Keep safe

The following steps are designed to ensure you feel safe whilst at work:

- Your supervisor should accompany you on first two home visits to each individual

- For each individual, your supervisor will undertake a CBR worker safety assessment of the risk to you and the supervisor
- You should always carry a mobile phone. You will be provided with credit.
- You should ensure that there is a family member present at all home visits. This should be emphasised to the caregiver during your first visits.
- If there is a high risk to your safety, your supervisor will accompany you on the home visits
- If there is a medium risk to your safety, you should call your supervisor at the beginning and the end of each visit

5. Know how to deal with a violent or aggressive person

- Be aware of the signs that someone is going to become violent. These include:
    - Talking louder or becoming threatening
    - Fists opening and closing
    - Breathing fast
    - Fidgeting
    - Tapping, punching or slapping tables, walls or the floor
- Be aware if the individual smells of alcohol, is losing their balance or has slurred speech. This might mean the individual is drunk and is more likely to be violent
- Think about your own feelings. If you feel scared you should stop the home visit and leave the house.
- Make sure that both you and the individual can reach the door of the house easily.
- Speak in a clear and calm voice. Do not shout to try and calm the individual.
- Let the individual know that you want to help them.
- Don't tell them that they can't really hear voices or that their beliefs are wrong.
- Do not threaten the individual or correct their swearing. This will only make it worse.
- If the individual has a weapon, reassure them that you are here to help them and there is no need for a weapon. If they refuse to put down or hand over the weapon, you should stop the home visit and leave the house.
- If the individual becomes violent tell him to calm down by firm reassurance. If this is not possible you should stop the home visit and leave the house.
- After the incident call your supervisor to tell them what has happened and agree together any steps you need to take now.

33.5 Summary

- Whilst working as a CBR worker you may sometimes feel stressed or unhappy
- This may happen if you feel you do not have enough support or you are worried about your safety or have other problems in your life to deal with
- It is important for you to stay well so that you can help the individuals you are working with properly
- Ways to stay well and happy include: reducing stress, discussing your work with others, seeking help if you need to, keeping safe and knowing how to deal with a violent person

34 Supervision

34.1 What is supervision?

Supervision is where somebody else supports you in your work and gives advice on how to improve the way you are working.

34.2 Why is supervision important?

It is important that you are supervised for the following reasons:

- To make sure that all the right steps are taken for each individual, for example that referrals to the health centre are made at the right time.
- To make sure all the individuals receiving CBR are supported in a similar way. Even though each individual will receive a different version of CBR, depending on their needs and goals, there are some things that should be the same for everybody. These include your relationship with the individual and how well you document their progress.
- To make sure all the kebeles have similar community engagement, for example community awareness raising.
- To make sure gaps in your knowledge are identified, so that you can receive extra training and support if you need it.
- To make sure you are safe and well whilst delivering CBR

34.3 Who is involved in supervision

You and the other CBR workers

You and the other CBR workers will get lots of experience with working with people with schizophrenia whilst delivering CBR. You will probably find it useful to share these experiences and hear about how others have overcome difficulties.

Your supervisor

Your supervisor will be your main source of supervision. They have experience in supervising CBR workers.

The intervention co-ordinator

The intervention co-ordinator will supervise your field supervisor. You will not have very much contact with the intervention co-ordinator.

34.4 How will you be supervised?

There are four ways in which you will be supervised.

1. **Face to face discussion**

Who? You and your supervisor

When? Every 15 days at a pre-arranged meeting

Where? At the health centre or a health post

What?

- Using the most recent Home Visit Forms (Form 11) and the logbooks you will discuss the progress of each individual you are working with, including:
    - General health and symptoms
    - Whether they are taking medication
    - Whether any risks have been identified, particularly suicide risk
    - Modules you are working on and modules you have completed
    - Progress on current goals
    - Any problems or difficulties achieving the goals
    - Practical issues
    - Safety issues
- You will record any actions that your supervisor suggests on the most recent Home Visit Form. You will need to make sure you use these suggestions in your next home visit. At the next Face to Face Discussion your supervisor will check whether you used the suggestions.
- Using your kebele logbooks you will discuss the progress of the community engagement, including:
    - Community engagement activities carried out and planned
    - Any problems with community engagement
- You will discuss your timetable for the next fortnight. Both you and your supervisor will then have a record of which individuals you plan to visit, and what community engagement activities you plan to do, on each day. See Form 13.
- Your supervisor will identify any training needs that you have and update your knowledge or skills if needed. You should record this part of your discussion on the Supervision Record Form (Form 21).
- You will arrange your next supervision meeting and any joint home visits which are needed

2. **Phone contact**

Who? You and your supervisor

When? Whenever you need advice or support

Where? By mobile phone

What?

- You can call your supervisor at any time to discuss problems or questions which arise during your work
- There are certain situations where you should always call your supervisor. These include:
  - Individual is at risk of suicide, chaining, neglect, physical or emotional abuse inside or outside the home, or sexual violence (see Chapter 14 and Chapter 35)
  - You have made an urgent referral for the individual to attend the health centre (see Chapter 36)
- You will record any suggestions that your supervisor makes on the most recent Home Visit Form, if it relates to an individual, or on the Supervision Record Form if it relates to your own training needs. You will need to make sure you use these suggestions in your next home visit.

3. Unannounced observed home visits

Who? You and your supervisor

When? About every month to two months, but you will not know the time or day in advance

Where? At an individual's home

What?

- You will carry out the home visit as normal, but your supervisor will also come to the home and observe you working. They will be looking at:
  - How good your relationship is with the individual and family
  - How well you listen and explain things to the individual and family
  - Whether you do modules or tasks which seem right for the individual at that time
  - How well you write down what happened during the home visit
- After the home visit, usually at the health post or health centre, the supervisor will tell you how well they think you are working. They will always give you some positive feedback, as well as telling you about any problems.
- You will discuss together how you can improve things if there are any problems. Your supervisor may give you more training if you need it.
- You will record any suggestions that your supervisor makes on the current Home Visit Form if it relates to the individual, or on a Supervision Record Form if it relates to your own

training needs. You will need to make sure you use these suggestions in your next home visit.

4. **Group supervision**

Who? You, all the other CBR workers and both field supervisors

When? Every month

Where? At a health centre

What?

- Prior to the meeting you should try to think of one or two specific problems or issues that you have faced that you would like some support with. These might be related to:
    - Issues with delivering the modules e.g. the best way to give advice about self care
    - Issues with working with people with schizophrenia e.g. How to react when an individual is unwell and hallucinating
    - Issues with community engagement work e.g. how to work with an obstructive community elder
    - Practical issues e.g. how to use public transport to get to a particular location
    - Your own well being e.g. how to balance work and home life
- During the session the supervisor will facilitate the discussion. They will ask one of you to briefly present your problem or question. The other CBRWs will then have a chance to ask you questions to make sure everyone understands what your issue is.
- The supervisor will then ask for ideas and advice from you and the other CBR workers for how to overcome the problem. It may be useful for other CBR workers to describe how they responded to a similar situation.
- You will record any suggestions that the group makes on a Supervision Record Form. You will need to make sure you use these suggestions, for example at your next home visit.
- The supervisor will then ask the next CBR worker to present their problem. In each session around half of the CBR workers will have chance to present their issue.
- The supervisors will also update your knowledge or skills based on issues that come up during the group discussion, or which they have noticed from face to face discussions or observed home visits.

34.5 Summary

- It is important that you are supervised to make sure all the right things are being done for each individual; to make sure your skills are up to date and that you are safe
- Every 15 days you will have a Face to Face Discussion with your supervisor to discuss each individual and kebele you are looking after, your timetable, and any training needs
- You can call your supervisor at any time for support. You should also call them when you identify certain risks or if you have made a referral to the health centre
- Your supervisor will make an unannounced home visit about once a month
- You will have a Group Supervision session every month to discuss issues with other CBR workers and share ideas.
- You should record any supervision on the most recent Home Visit Form or a Supervision Record Form

35 Dealing with difficult situations

35.1 What difficult situations might you come across?

As you learnt in Chapter 14, people with schizophrenia are vulnerable to many types of risks. These include: suicide attempt or suicide risk, chaining, neglect, physical abuse outside the home, physical or emotional abuse inside the home and sexual violence.

Other difficult situations you might come across include:

- The individual commits suicide or dies from another cause
- The individual is admitted to hospital due to a serious side effect from the anti-psychotic medication, or due to any other serious medical emergency
- The individual is violent or aggressive towards you or others

35.2 Why is it important to know how to deal with difficult situations?

It is important that you know how to deal with difficult situations so that the individual can get the best care and support available as soon as possible. This will lead to a better outcome for the individual, family and the community.

35.3 How should you deal with difficult situations?

In this section there is a summary of what you should do for each difficult situation. Your priorities when dealing with difficult situations are always to ensure your own safety and the safety of the individual. Whenever you send the individual to the health centre, write on the Health Centre Referral Form (Form 14) the reason for the referral and ask the individual or caregiver to show the form to the nurse or health officer. Keep a copy of the form for the participant logbook. You should only call the police if you strongly believe the individual is in immediate danger of being seriously hurt by someone.

KEY

Solid arrow: always continue to the next step

Dashed arrow: continue to the next step if the red writing is true in this situation

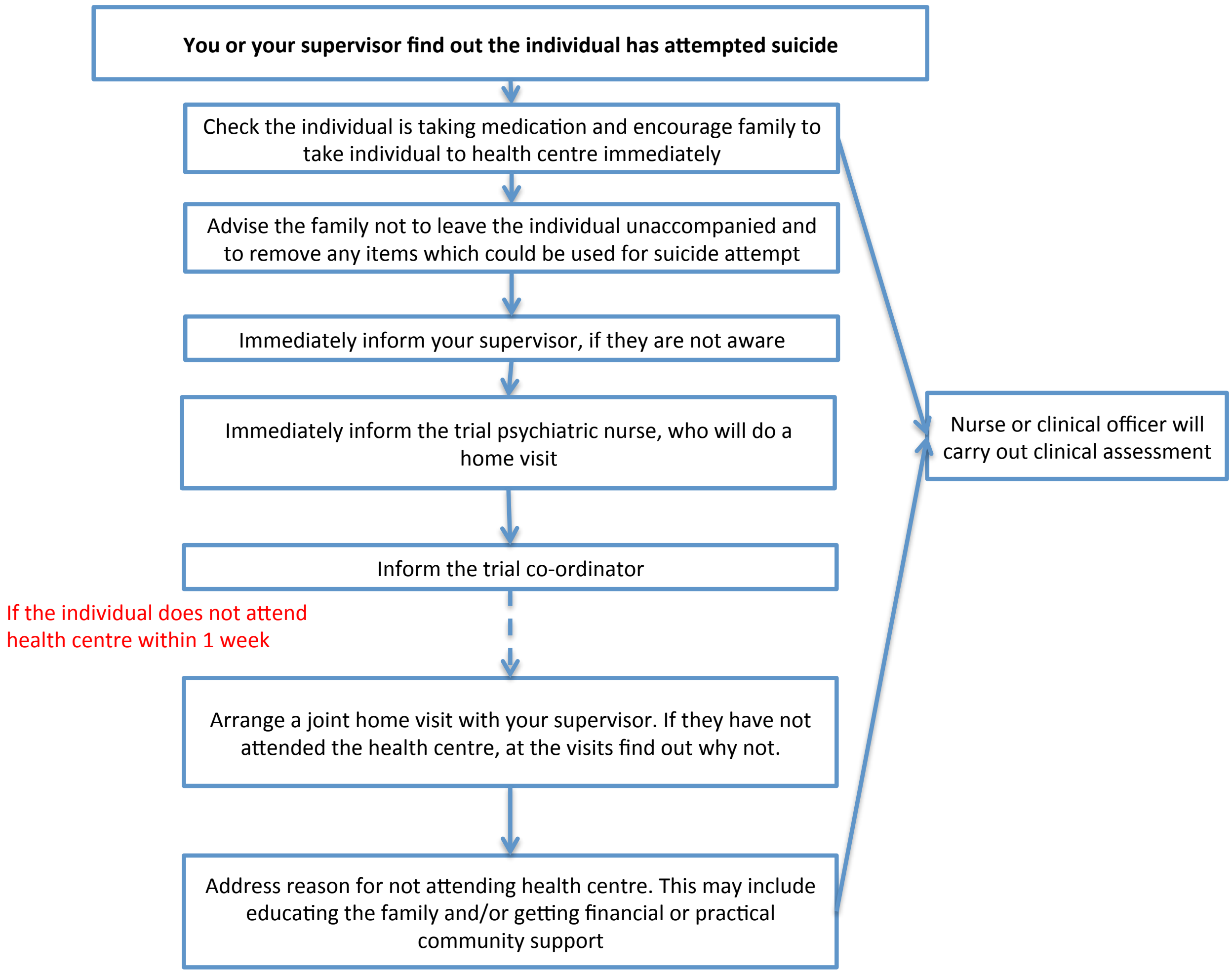

Figure 1 Attempted suicide flow chart

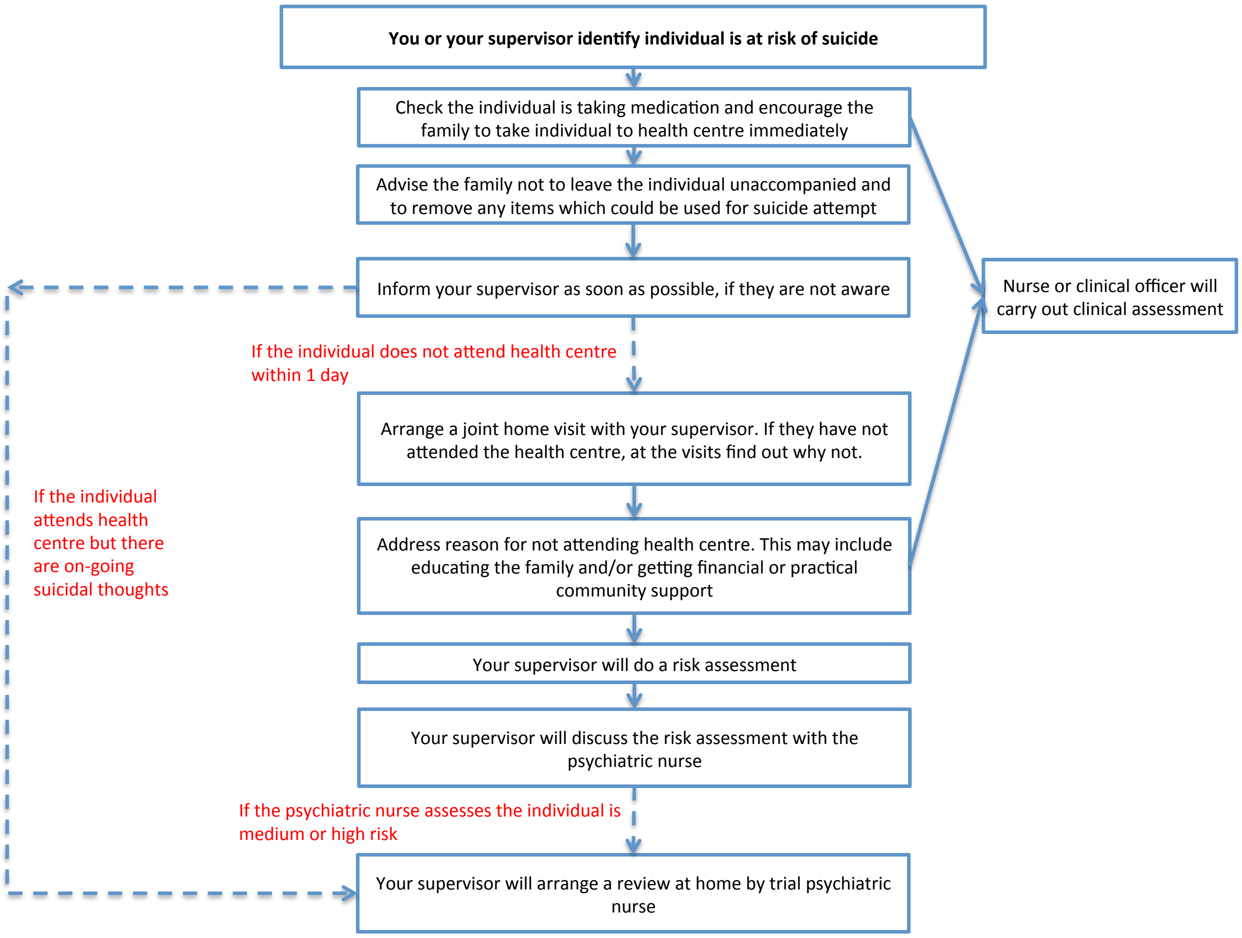

Figure 2 Risk of suicide flow chart

The individual is violent or aggressive

Take steps to ensure your own safety:
•Make sure that both you and the individual can reach the door of the house.
•If the individual has a weapon, reassure them that you are here to help them and there is no need for a weapon
•If you feel scared you should stop the home visit and leave the house.

Take steps to calm the individual:
•Speak in a clear and calm voice. Do not shout to try and calm the individual.
•Let the individual know that you want to help them.
•Don't tell them that they can't really hear voices or that their beliefs are wrong.
•Do not threaten the individual or correct their swearing.

Check the individual is taking medication and encourage them to do so

Encourage family to take individual to health centre

Inform your supervisor after the incident and agree together any steps you need to take now. Do not visit the home until you have permission from your supervisor

Your supervisor may arrange for review at home by trial psychiatric nurse

Figure 3 VIolent or aggressive individual flow chart

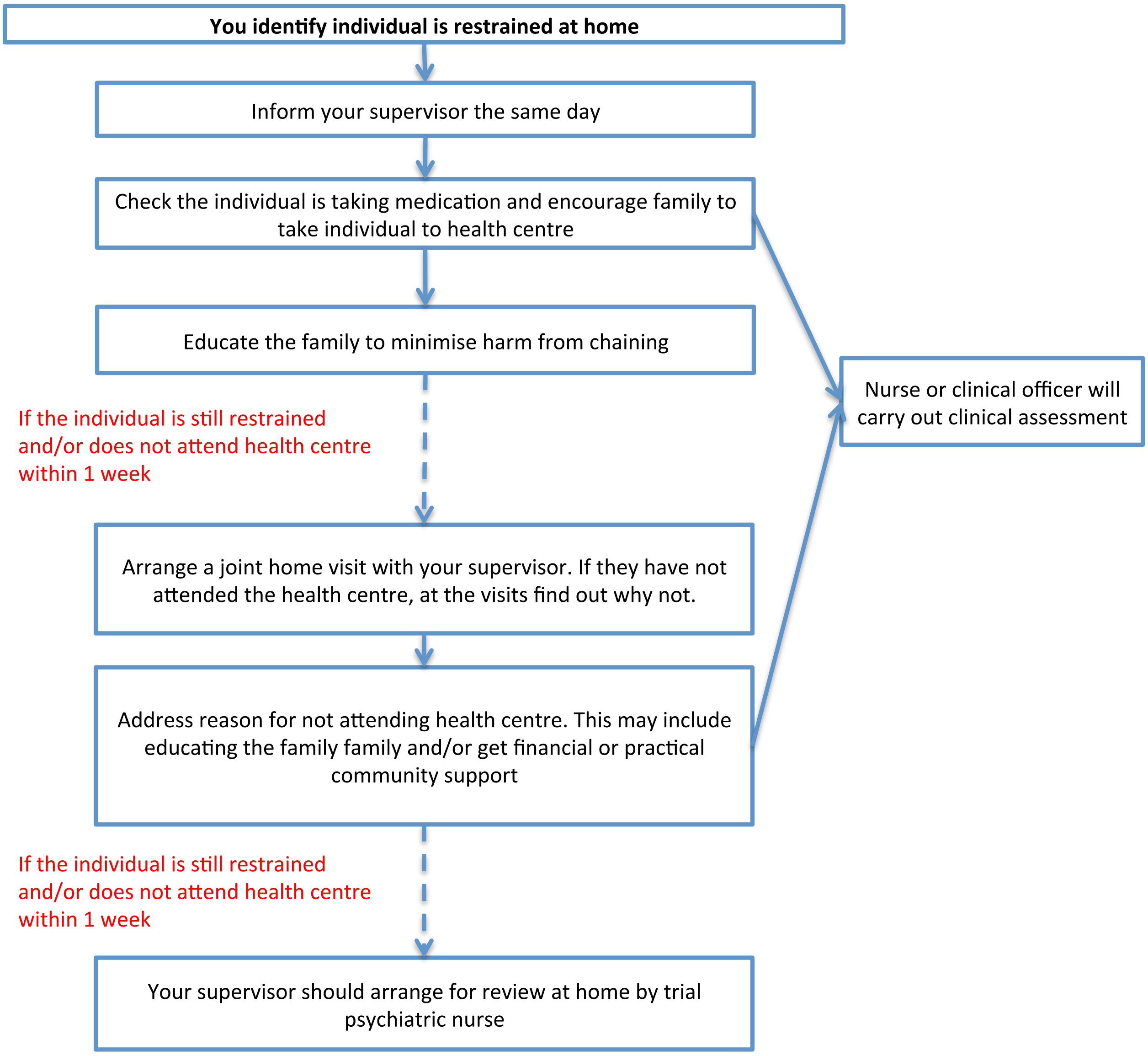

Figure 4 Restrained or chained individual flow chart

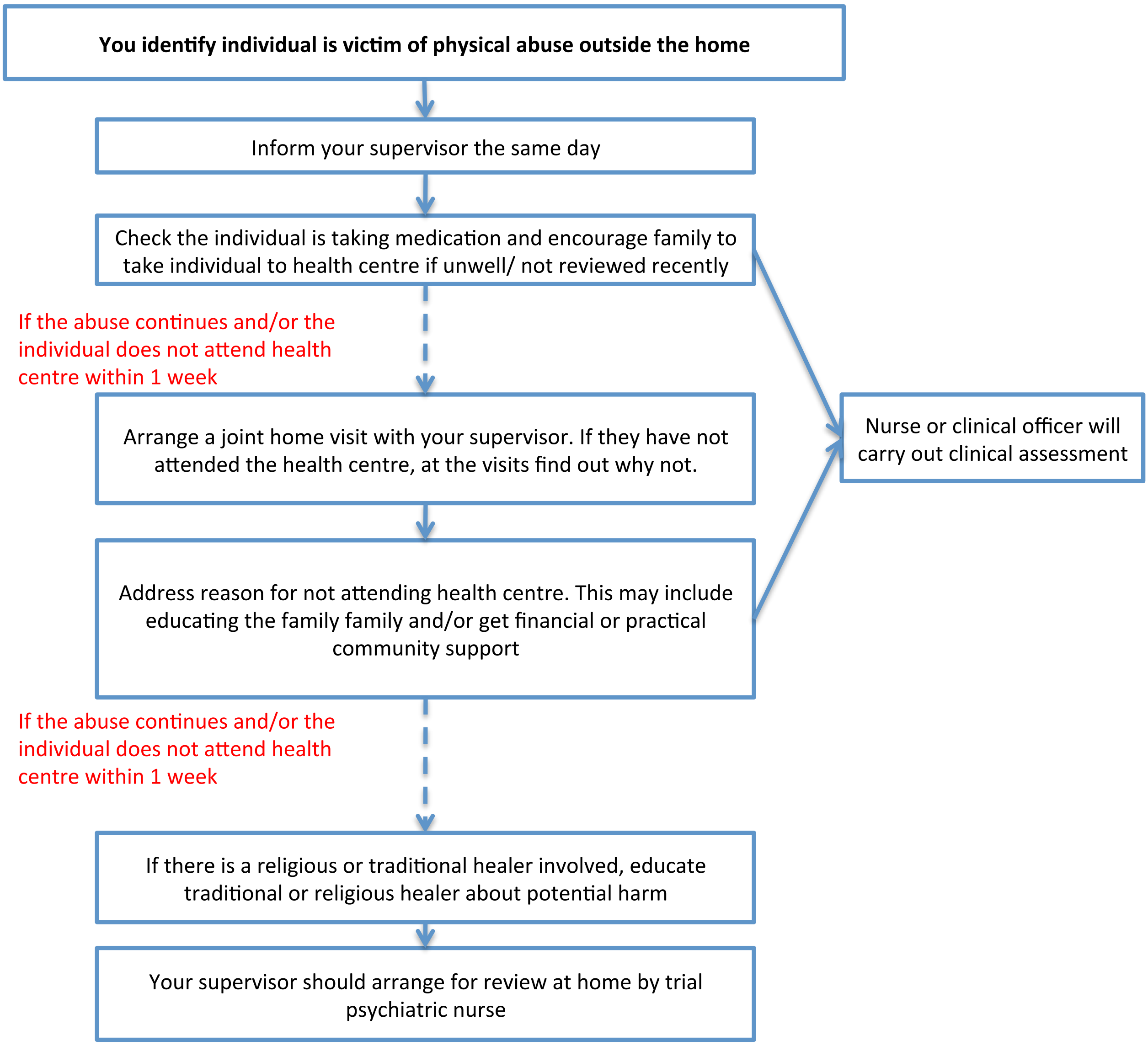

Figure 5 Physical abuse outside the home flow chart

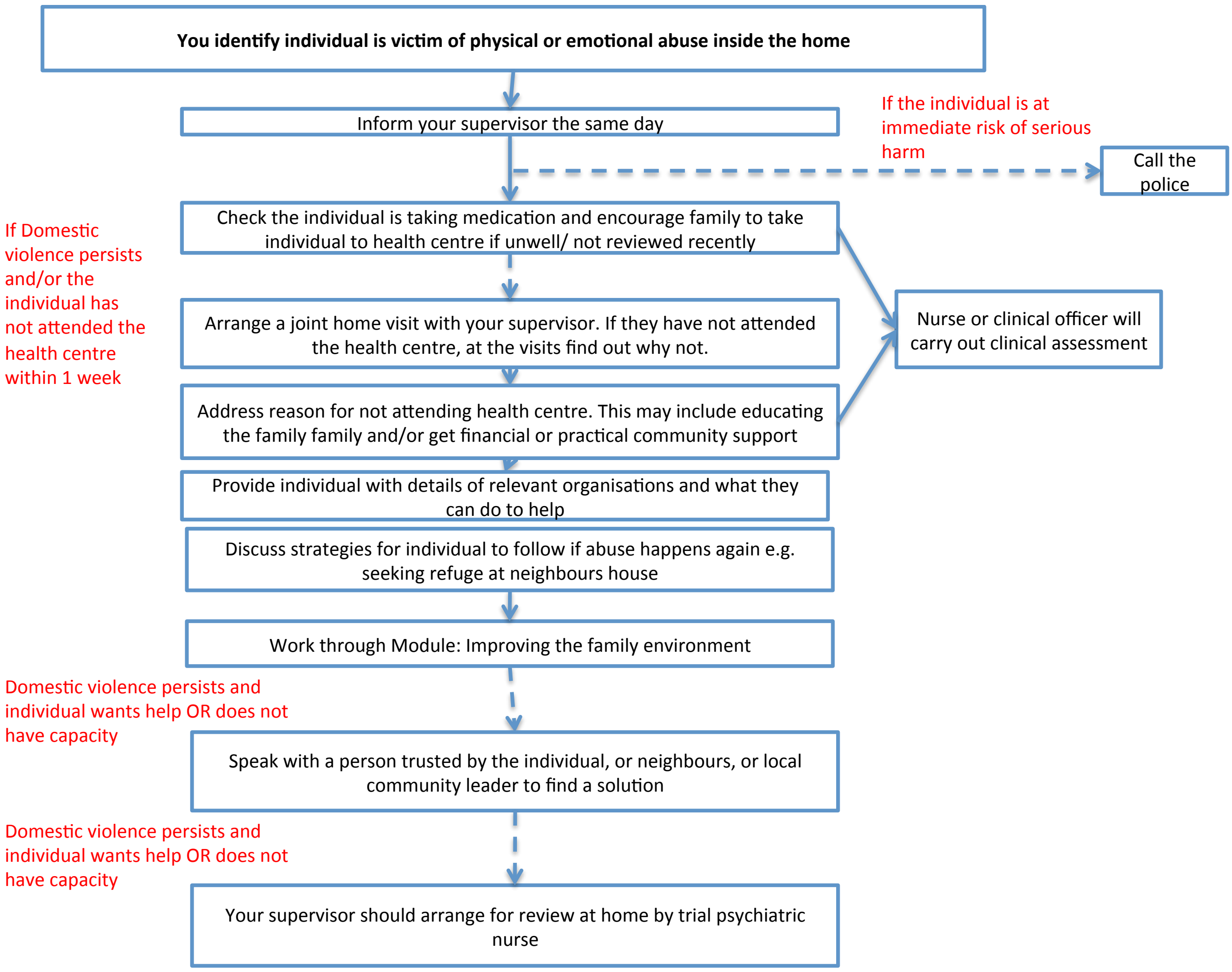

Figure 6 Physical or emotional abuse inside the home flow chart

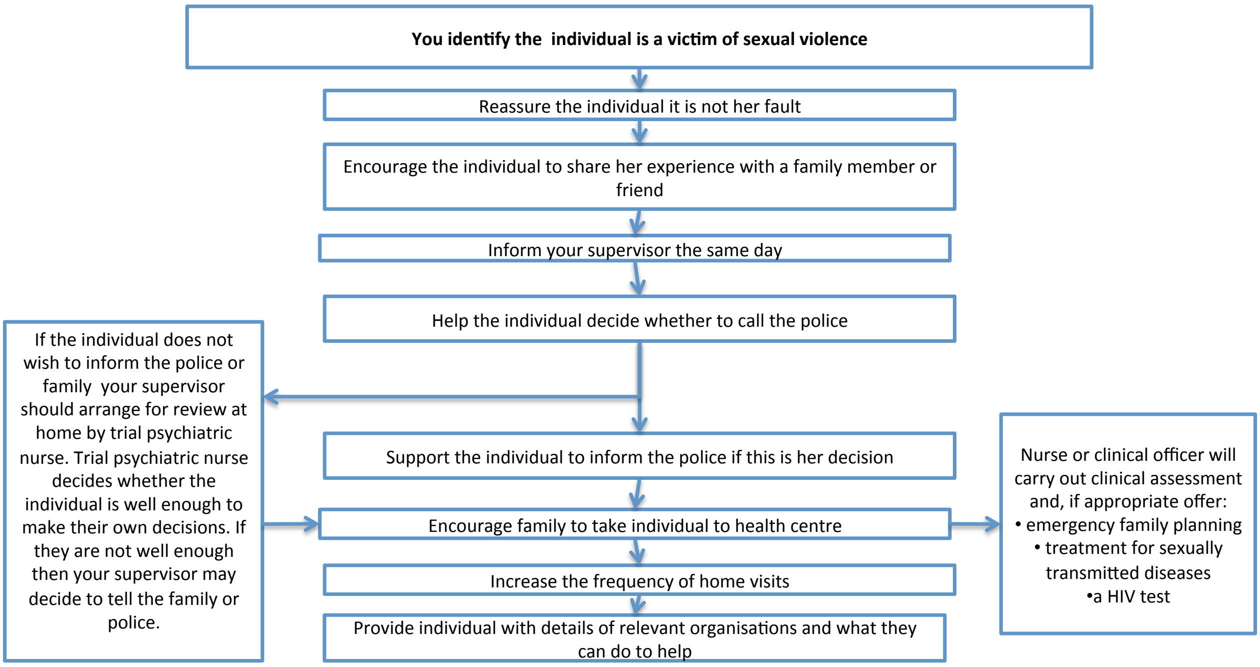

Figure 7 Sexual violence flow chart

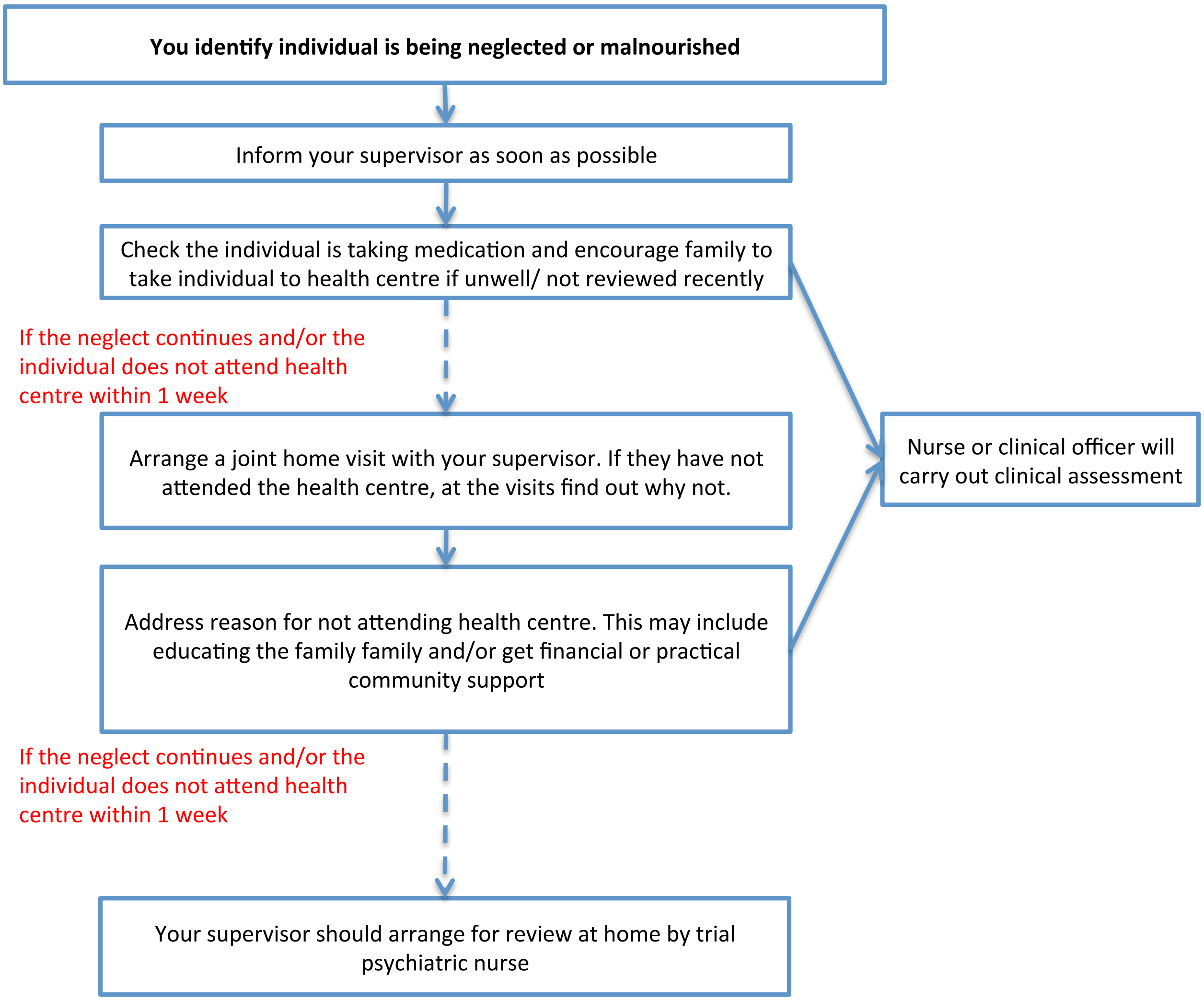

Figure 8 Neglected or malnourished flow chart

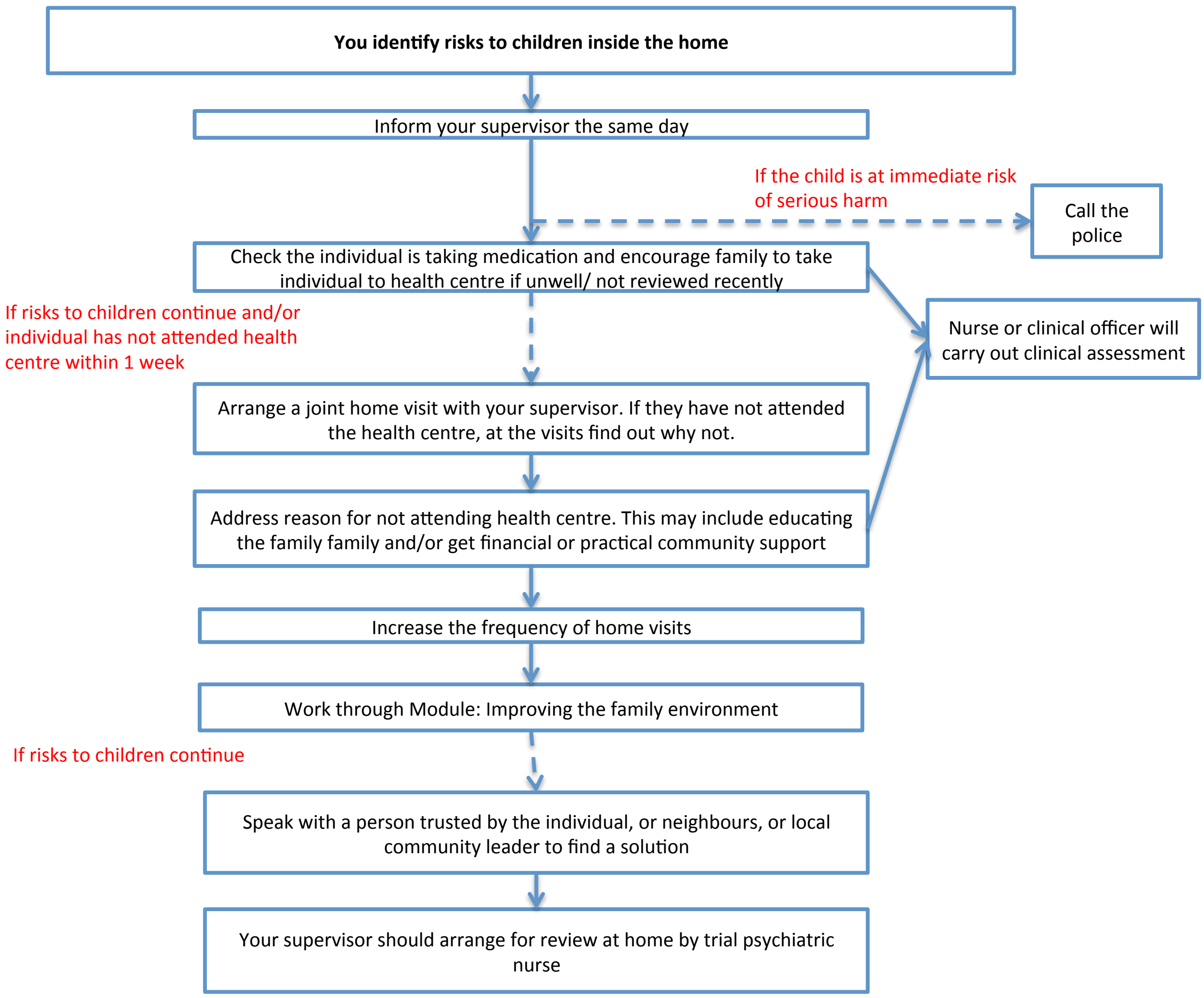

Figure 9 Risks to children flow chart

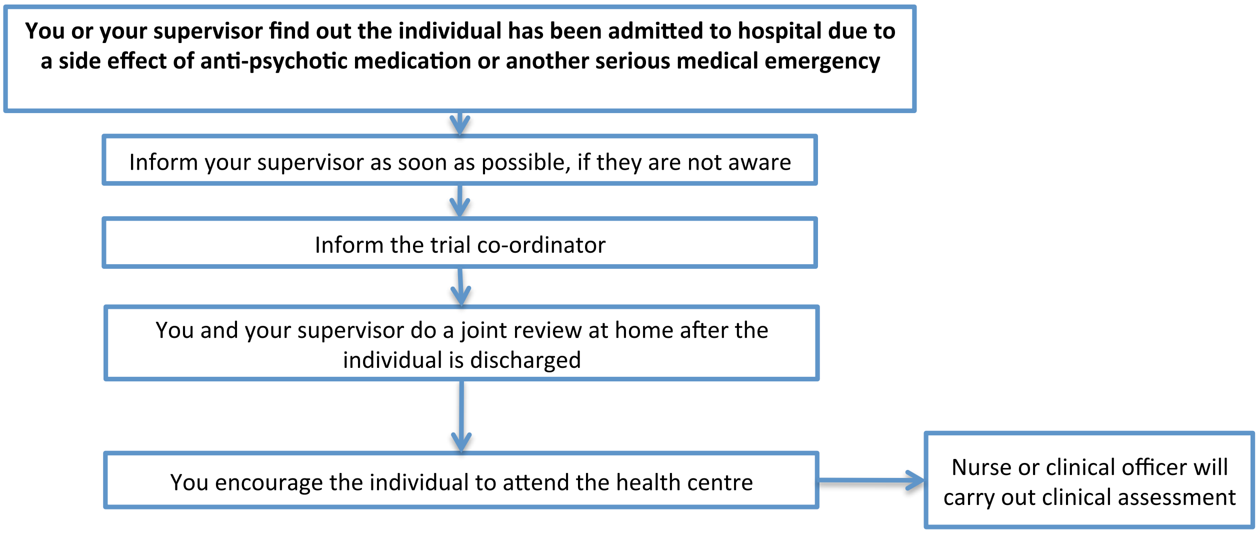

Figure 10 Hospitalisation flow chart

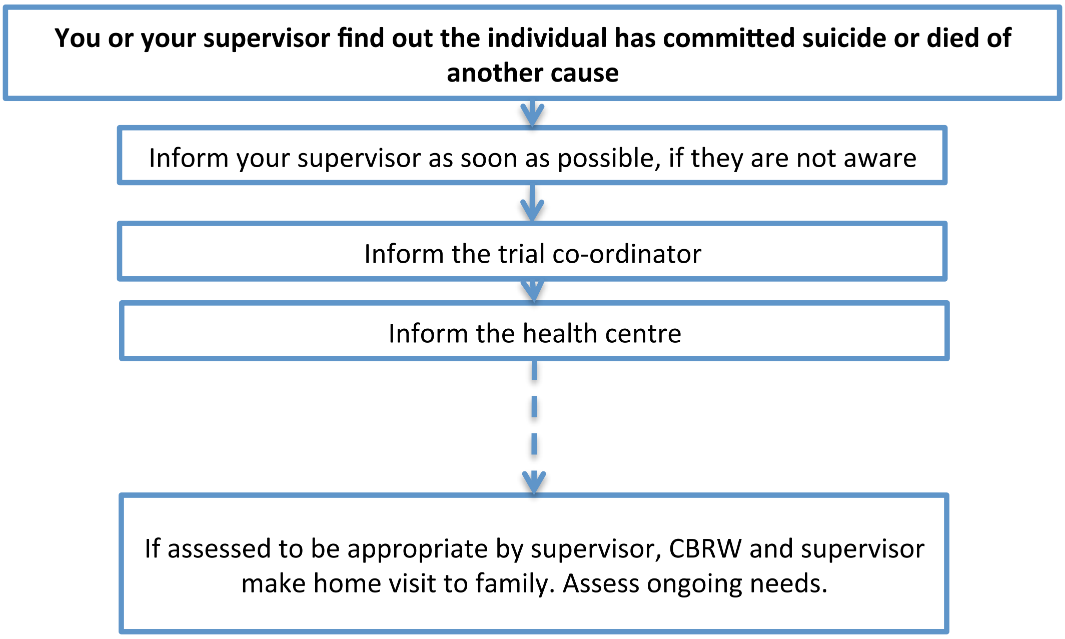

Figure 11 Suicide or death from other cause flow chart

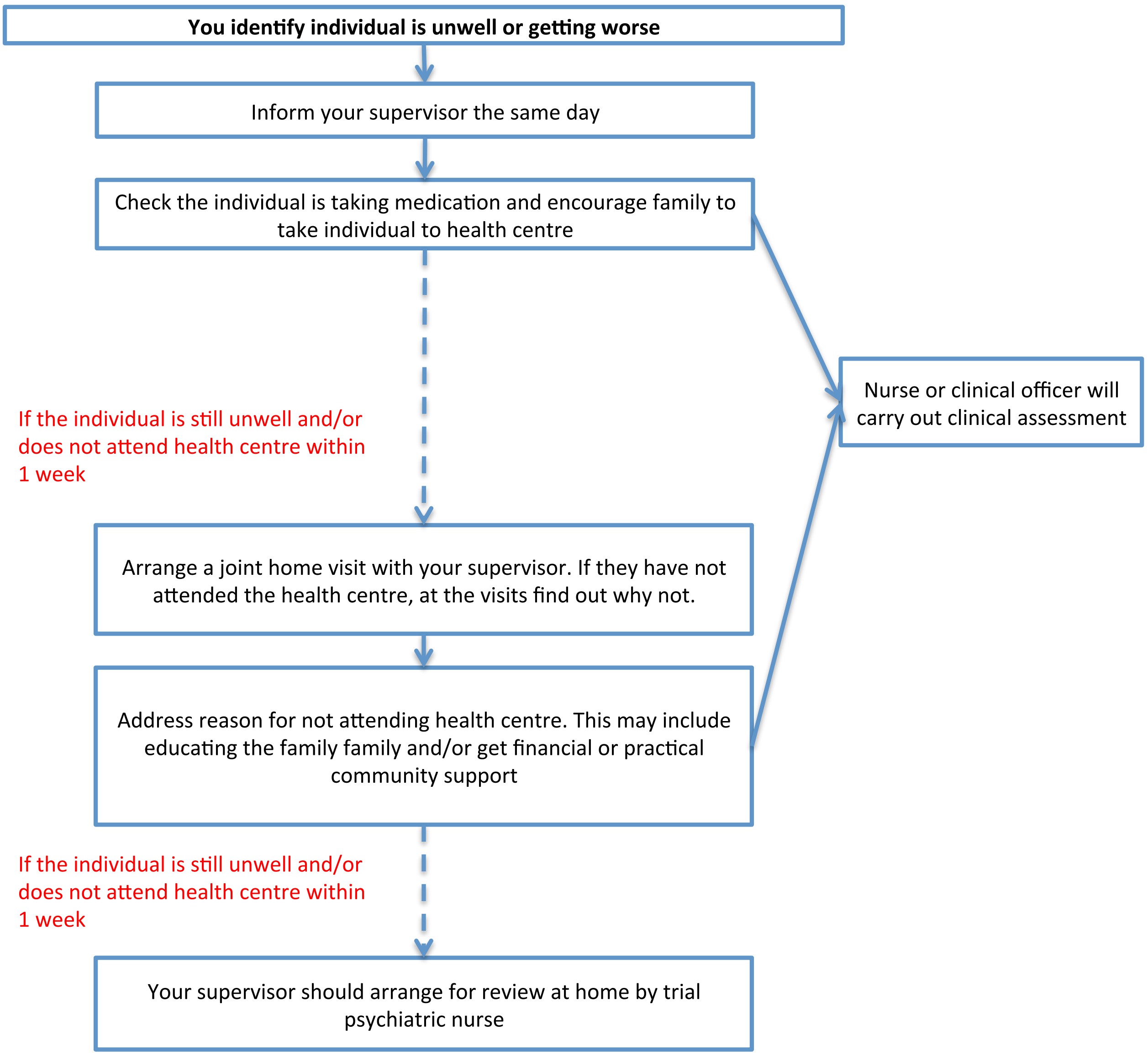

Figure 12 Individual is unwell or getting worse

35.4 Summary

- It is important you know how to deal with difficult situations to make sure the individual gets the best care possible
- There is a set of actions you should take for each difficult situation

36 Links to health services

36.1 What are links to health services?

Links to health services are any communication that you have with:

- The health centre
- The health extension workers

36.2 Why is it important to have links to health services?

All the individuals you are working with have access to medical care for schizophrenia at the health centre. It is part of your role to ensure that the individual attends the health centre regularly, as this will help them to recover (see Chapter 17). There are some situations that are too complicated for you to deal with, for which you should send the individual to the health centre. There are other types of work, for example giving advice about family planning, which you have not been trained for, so you should involve the health extension worker. Keeping links with health services will ensure the individual can get the best possible care.

36.3 How do you keep links to health services?

Introduce yourself at the health centre

At the beginning of the CBR programme you and your supervisor will introduce yourself to the health centre head and the nurses and clinical officers. They will already be aware of the CBR programme but you can remind them of your role.

Regular appointments at the health centre

Each individual should go to the health centre every 1 to 3 months. You should be aware of all the appointments they have, and check if they have attended. If they have missed an appointment, even if you are not due for a home visit, you should make a home visit to check for any problems and remind them to attend.

Special situations

You should send the individual to the health centre in any of the following situations:

- You think the individual is at risk of committing suicide (See Chapter 14 and 35)
- You are worried that the individual is not eating or drinking (See Chapter 35)

- You are worried that children may be at risk of harm from the individual (See Chapter 35)
- The individual has harmed someone else or you are worried this is going to happen
- The individual has a relapse i.e. is very unwell with symptoms of schizophrenia (Chapter 30)
- The individual become pregnant. You should also inform the psychiatric nurse.
- The individual has a new physical health problem or physical injury (Chapter 21)
- The individual has a serious problem with alcohol or khat (Chapter 21)
- The individual has serious side-effects of the anti-psychotic medication (see Chapter 20 for description of side effects).
- You would like the nurse to review the medication schedule or consider the injection (Chapter 20)

You should tell your supervisor if any of these situations happen. Advise the family to take the individual to the health centre as soon as possible and give them support in making the journey. This may include finding other community members who can help the family (see Chapter 31). Record on the home visit form that you have referred to the health centre. Write on the Health Centre Referral Form (Form 14) the reason for the referral and ask the individual or caregiver to show the form to the nurse or health officer. Keep a copy of the form for the participant logbook. You can go to the health centre with the individual. If you accompany the individual you should complete a Health Centre Contact Form (see Form 2). Once they have returned from the health centre you should continue to deliver CBR as normal. If you find that the caregiver is very distressed you can suggest they go to the health centre (see Chapter 15).

Health Extension Programme

The health extension workers should be continuing to provide their usual care to the individuals you are working with. You should ask the health extension worker to visit the individual if they need support with contraception, sanitation advice, or malaria prevention, and they have not received a home visit. Health extension workers are not expected to assist with CBR, for example help with self-care.

36.4 Summary

- It is important to have links with the health centre and health extension worker to ensure the individual has the best care possible.
- You should send the individual to the health centre when: they are at risk of committing suicide, they are not eating or drinking, they have harmed someone else, they are very

unwell with schizophrenia, they are pregnant, they have a physical health problem or they have serious side effects of anti-psychotic medication.

- You should ask the HEW to visit if they have not delivered their usual care to the individual
- If an individual misses a routine appointment at the health centre you should remind them to attend

37 Good documentation

37.1 What is good documentation?

Good documentation means that you write things down, for example about a home visit, in a way that:

- You are able to look back and remember what happened.
- Other people, for example your supervisor, can understand what happened without having to ask you
- The individual and family can read what you have written and they think it is a good description of what happened during the visit

37.2 Why is it important to have good documentation?

When you are doing CBR with many families at the same time it may be difficult to remember the details of what happened every time you see a family. By keeping a good record of each individual's progress, you will be much better at helping them each time you see them. It will also be easier for your supervisor to check that you are looking after individuals in the right way, and give you advice on how to improve the way that you are working. The individual and family you are working with may ask to see what you have written. It is fine for you to show them.

37.3 What do we need to document?

For each individual you are looking after you will keep an individual logbook. This will include the following forms:

- Initial contact form (Form 1)
- Health Centre Contact Form (Form 2)
- CBR Review Form (Form 3)
- Needs Assessment Form (Form 4)
- Goals setting Forms (Forms 5 and 6)
- Risk assessment Form (Form 7)
- Rehabilitation plan (Form 8)
- Continuing Care Form (Form 9)
- Home visit forms for each visit (Form 11)
- Visit Summary Forms (Form 12)
- Health Centre Referral Form (Form 14)

- Medication checklist (Form 15)
- Day to Day functioning Progress Form (Form 16)
- Early Warning signs checklist (Form 17)
- Relapse Management Plan (Form 18)
- Supervision Record Forms (Form 21)

For each kebele you are working in you will keep a kebele logbook (Form I). You have learnt about what information to record in these logbooks in other sections of the manual.

37.4 How do we make sure we have good documentation?

These are the things you can do to make sure you have good documentation:

- Write in a clear and legible way
- Write in Amharic
- Write down enough detail so that you and someone else will be able to understand it later
- Make sure you would be happy for the individual and family to read whatever you write down.
- Use a pen instead of a pencil
- Keep all your logbooks with you at all times when you are working in the field. At other times keep them in a safe place.
- Keep your logbooks dry and clean
- Your supervisor will check your documentation every so often and will give advice on how to improve it if necessary

37.5 Summary

- Good documentation is where you record things in a way it is easy to understand later
- It is important to make sure you and your supervisor can easily follow the progress of individuals
- You will keep an logbook for each individual and each kebele
- You can ensure good documentation by: writing clearly, keeping logbooks dry and clean, writing in pen and writing enough detail

38 How to deal with other people with problems

38.1 What other problems might you come across?

During your CBR work you may come across people, apart from the individuals you are working with, who have problems. These might include:

- People with schizophrenia who have been offered care at the health centre but who you have not been asked to look after. They might live in a kebele where you are working, or another kebele where you are not working.
- People who you think may have schizophrenia but who are not receiving care at the health centre
- People with other sorts of mental health problems, for example alcohol problems in adults, or developmental delay in children
- People with other sorts of disabilities, for example blindness or problems walking due to polio
- Community members or community leaders may ask you to do community engagement work in another kebele

38.2 Why might you come across other people with problems?

In most kebeles there will be people with schizophrenia who are receiving care at the health centre but who you have not been asked to look after. The reasons for this will be:

- They are generally more well or less disabled so do not need the extra support of CBR
- They were offered CBR but they did not want to participate
- There is another reason why they could not receive CBR, for example there was no caregiver able to participate, or they have plans to leave the kebele soon.

You may be asked by the individual, family or community members to offer CBR or extra support to these individuals. It may be difficult for people to understand why you are giving extra support to some people with schizophrenia but not others.

You will become good at recognising schizophrenia, and you may notice the symptoms in people who have not been diagnosed by a nurse at the health centre. Or community members might tell you about individuals who are chained up because they are unwell.

When you are working within the kebele you might also notice other sorts of problems, for example physical disabilities, just by observing the people you meet. The family you are working with might tell you about problems with other family members. Community leaders or other

community members may see you as a source of support and may tell you about problems that other people have, even if you don't ask them to.

Community members or community leaders from kebeles which have not been allocated to receive CBR may ask you to do community engagement work in their kebele. They may have seen or heard about you doing this work in the kebeles where you are working.

38.3 Why is it important to know what to do if you come across other people with problems?

In this CBR programme your role is to support the people with schizophrenia that you have been asked to look after. However, it is important that you know what to do if you come across people with other problems. This is to make sure that these people get any care and support that is available. It will also help the individual, family and community to trust you more if you know what to do when you come across people with other problems.

38.4 What should you do if you come across people with other problems?

What you should do when you come across other people with problems depends on the type of problem. But the general rule is to ask someone else to help, rather than helping the person yourself.

1. People with schizophrenia who have been offered care at the health centre but whom you have not been asked to look after

- Suggest that they visit the health centre if they have not been recently
- Explain that unfortunately you cannot offer home visits to them and that this is out of your control.
- Ask the Health Extension Worker to support the individual, for example encouraging them to attend the health centre.
- If the individual lives in kebele where you are working (i.e. in a kebele allocated to receive CBR), consider inviting them to the Family Support Group, if this has already started.
- If the individual lives in kebele where you are not working (i.e. in a kebele not allocated to receive CBR), it is important that you do **not** invite them to the Family Support Group.
- Inform your supervisor of your discussions

2. People who you think may have schizophrenia but whom have not yet been offered care at the health centre

- Suggest to the individual and family to take the individual to the health centre
- Ask the Health Extension Worker to also encourage them to attend the health centre and to follow up on their progress.
- Inform the health centre that you have asked the individual to attend
- Inform your supervisor

3. People with other sorts of mental health problems

- Suggest to the individual and family to take the individual to the health centre
- Ask the Health Extension Worker to encourage them to attend the health centre and to follow up on their progress.
- Inform the health centre that you have asked the individual to attend
- Inform your supervisor

4. People with other sorts of disabilities

- Suggest to the individual and family to take the individual to the health centre
- Ask the Health Extension Worker to encourage them to attend the health centre
- Inform the health centre that you have asked the individual to attend
- Inform your supervisor
- Refer the individual to any relevant NGOs

5. Community members or community leaders who want you to do community engagement work in another kebele

- It is very important that you do NOT do any community engagement work in kebeles where you are not already working (i.e in kebeles not allocated to CBR).
- Explain that unfortunately you cannot offer community engagement work in their kebele and that this is out of your control.
- Explain that medical care for people with schizophrenia is available at the health centre and that anyone with schizophrenia who is not already doing so should seek help there.
- Give them the number of the trial co-ordinator in case they wish to discuss this further
- Inform your supervisor of your discussions.

38.5 Summary

- During CBR work you may come across other people with problems, for example people with mental health problems, or people with physical disabilities
- It is important to know what to do so that these people can get the best care possible
- Generally, you should not try to look after these people yourself, but you should tell them how to get help

39 Supporting people with intellectual disability and schizophrenia

39.1 What is intellectual disability?

Intellectual disability affects the way a person understands information and how they communicate. This means they can have difficulty:

- understanding new or complex information
- developing as fast as other people
- learning new skills
- living independently

Some intellectual disabilities are diagnosed at birth, such as Down's syndrome. Others might not be discovered until the child is old enough to talk or walk. It is possible to have both schizophrenia and an intellectual disability. Some of the individuals you support through CBR may have both schizophrenia and an intellectual disability.

39.2 What causes intellectual disability?

An intellectual disability happens when a person's brain development is affected, either before they are born, during their birth or in early childhood. Several factors can affect brain development, including:

- the mother becoming ill in pregnancy
- problems during the birth that stop enough oxygen getting to the brain
- illness, such as meningitis, or injury in early childhood

Sometimes there is no known cause for an intellectual disability. Intellectual disability is not due to God punishing children or parents for sins, or due to curses or bewitchment. Neglecting or punishing the person for being slow or having difficult behaviour is likely to make things worse.

39.3 What problems do people with intellectual disability have?

Just like people with schizophrenia, people with intellectual disabilities can have different kinds of disabilities. An intellectual disability can be mild, moderate or severe. Some people with a mild intellectual disability can talk easily and look after themselves, but take a bit longer than usual to learn new skills. Others may not be able to communicate at all and have more than one disability.

Some people with intellectual disabilities can be quite independent, while others need help with everyday tasks, such as washing or getting dressed, for their whole lives. It depends on their abilities. People with intellectual disability are more likely to have the following problems compared to other people

- Physical problems, for example problems moving and walking; problems with hearing, speech and sight; epilepsy
- Mental health problem, including schizophrenia.
- Problems sleeping
- Behaviour problems, including doing things that threaten their own safety, or the safety of other people; and tantrums

People with intellectual disabilities may also experience stigma and discrimination, or be vulnerable to exploitation and abuse.

39.4 How should we communicate with people with intellectual disability?

1. Ensure you have the person's attention. Use their name and use eye contact
2. A person's ability to understand may vary, for example it may be worse when they are tired or upset. When you're not sure of the person's ability to understand, it is more respectful to assume they DO understand rather than they DON'T
3. If you're not sure, check with the caregiver how the individual communicates e.g. how do they say yes/no?
4. When you speak to the person, use simple and clear words and short sentences.
5. Speak to the person with the same respect that you would any other adult, rather than talking to them like you would talk to a child.
6. Give the person lots of time to understand what you have said and to respond.
7. Check the person's understanding in their own words.
8. If you don't understand the person, you should say so. Don't pretend to understand. Keeping trying to understand e.g. could you use another word to explain? Can you show me?
9. If the person doesn't understand what you are saying, don't give up. Keep trying, but say it in a different way. Try using pictures or actions to explain.
10. Involve the caregiver to help you to communicate- but first of all ask the person if that's ok.

39.5 What is the treatment for intellectual disability?

There is no specific treatment for intellectual disability, unlike for schizophrenia. If a person has both schizophrenia and intellectual disability they will usually be given anti-psychotic

medication. If a person has only intellectual disability, they will not usually be given any medication, as this is unlikely to help. However, if they also have another condition, such as epilepsy, they may be given medication for this.

39.6 Can people with intellectual disability recover?

Intellectual disability is usually lifelong, but some problems may improve. People with intellectual disability may continue developing and learning but at a slower rate compared to other people. Supporting the individual and their family you can help increase the different kinds of activities the individual does and to increase their quality of life.

39.7 Which RISE CBR modules should we use for people with intellectual disabilities and schizophrenia?

If an individual has both intellectual disability and schizophrenia, many of their needs may be the same as for individuals with only schizophrenia. However there may be some differences. For example, there may be more need to focus on the module 'Improving day to day functioning'. Progress may be slower amongst individuals with intellectual disabilities, so you should make sure family members do not expect to see rapid changes in the individual. Also when covering the module 'Improving the family environment', the family may need particular support in coping with challenging behaviour.
