# Supplementary material for: “Like a doctor, like a brother”: Achieving competence amongst lay health workers delivering community-based rehabilitation for people with schizophrenia in Ethiopia
Source: PLoS One. 2021 Feb 25;16(2):e0246158. doi: 10.1371/journal.pone.0246158 (PMC7906313; doi:10.1371/journal.pone.0246158)
Supplement: S3 Appendix — (PDF) [file pone.0246158.s003.pdf]

Date ___ ___ / ___ ___ / ___ ___ ___ ___
Day Month Year

CBR Worker name __________CBR worker ID:___ Participant ID:_______Location of assessment__________________
Name of the person completing the form:______________________ Assessor's ID:______
Job position: ____________ Visit type:______________

| | | |
|---|---|---|
| **1. Non-Verbal Communication & communication through active listening and with the use of appropriate body language** ☐ **Not applicable** | | |
| 1 | **Needs improvement** | = does not make appropriate eye contact with the patient or stares; shows anger; laughs at/mocks patient; turned away from patient; repeatedly interrupts the patient conversation; ignores patient; answers mobile phone without permission |
| 2 | **Done Partially** | = does not consistently use body language to express interest: rarely makes eye contact, expresses only limited emotion, appears artificial; |
| 3 | **Done Well** | = makes appropriate eye contact throughout their conversation; smiles when appropriate; sits at appropriate angle from patient, leans in to the patient to show interest; use of 'uh-huh', 'hmm' and other keys to signal interest in their conversation |
| **2. Verbal Communication Skills: Open-ended questions, REPEATING THE MAIN TOPIC, clarifying statements** ☐ **Not applicable** | | |
| 1 | **Needs Improvement** | = Uses mostly 'yes/no' questions, e.g. "do you take your medication?" |
| 2 | **Done Partially** | = Uses open-ended questions, but does not explore topics further or does not repeats the main topics for patient to reflect upon |
| 3 | **Done Well** | = Uses Open-ended questions, repeats the main topic and clarifies statements, e.g.,asks questions like "What happened? Tell me more." |
| **3. building trust** ☐ **Not applicable** | | |
| 1 | **Needs improvement** | = clinician does not attempt to make the patient feel comfortable by treating him with respect and dignity |
| 2 | **Done Partially** | = Clinician does not attempt to make the patient feel comfortable but treats him with respect and dignity. |
| 3 | **Done Well** | = Clinician attempts to make the patient feel comfortable by treating him with respect and dignity. |
| **4. further Exploration, interpretation and normalization of feelings** ☐ **Not applicable** | | |
| 1 | **Needs improvement** | = clinician does not ask about patient's feelings **OR** clinician is judgmental/critical about patient's emotions and feelings (e.g., "You shouldn't feel that way" "You should stop thinking or feeling that." |
| 2 | **Done Partially** | = clinician asks but does not normalize (does not explain that it is common)/validate **OR** does not explore feelings in detail with patient ( Uses questions which need a Yes/No reply) |
| 3 | **Done Well** | = clinician explains that the patient's feelings are common and expected for a person in his/her situation |
| **5. Empathy, Warmth, & Genuineness** ☐ **Not applicable** | | |
| 1 | **Needs improvement** | = is critical, or hostile, of patient's concerns or complaints |
| 2 | **Done Partially** | = clinician is not critical or hostile but does not demonstrate that he/she understands the experience of patient or does not consider him seriously. |
| 3 | **Done Well** | = clinician demonstrates that he/she understands the experience of patient in genuine, sincere manner |
| **6. Assessing Impact of psychosocial problems on Life, functioning and day to day activity** ☐ **Not applicable** | | |
| 1 | **Needs improvement** | = clinician does not ask patient about the impact of stress, worry, thoughts and psychosocial problems on functioning and daily life. |
| 2 | **Done Partially** | = clinician asks functioning and day to day activities, but does NOT relate it to psychosocial concerns |
| 3 | **Done Well** | = clinician explores the relationship between psychosocial problem and functioning |
| **7. Explores patient's and social support network's Explanation for the cause of Problem (Causal Model)** ☐ **Not applicable** | | |
| 1 | **Needs improvement** | = clinician does not ask patient about his/her own view of the cause of his problem OR is judgmental/critical about patient's explanation (e.g. "Witchcraft doesn't cause these problems, that is an ignorant/backwards idea |
| 2 | **Done Partially** | = clinician asks patient about his/her own view of cause of problems, but does not explore if his/her view is similar to his family or other important people in support network. |
| 3 | **Done Well** | = clinician asks patient about his own view of the cause of his problem **and** asks if family or significant other support network have same or different explanations |

| | | |
|---|---|---|
| **8. Assessing coping mechanisms for problems and challenges and prior solutions** ☐ **Not applicable** | | |
| 1 | **Need improvement** | = clinician does not ask patient about how patient has coped with the problem **OR** clinician is judgmental/critical about how patient has coped (e.g., "Why did you think that work?" or "That isn't helpful.") |
| 2 | **Done Partially** | = clinician asks about coping and prior solutions, but does not provide positive feedback |
| 3 | **Done Well** | = clinician asks patient about how he has coped with the problem and provides positive feedback |
| **9. Assessing impact of patient's recent stressful life events on psychosocial wellbeing** ☐ **Not applicable** | | |
| 1 | **Needs improvement** | = clinician does not ask about current stressful situations/events |
| 2 | **Done Partially** | = clinician asks about current stressful situations but does not see its relation with current mental health issues |
| 3 | **Done Well** | = clinician asks about current stressful situations and discusses connection with current mental health |
| **10. assessing, Alcohol or Drugs USE (including misuse of prescription drugs)** ☐ **Not applicable** | | |
| 1 | **Needs improvement** | = clinician does not ask about drug and alcohol use (including misuse of prescription drugs) , OR asks about drug or alcohol use in an inappropriate or insensitive way for the patient's age and sex |
| 2 | **Done Partially** | = clinician takes partial history but does not explore positive responses about alcohol or drug use (including misuse of prescription drugs) |
| 3 | **Done Well** | = clinician assesses issues with alcohol or drugs (including misuse of prescription drugs) and explains relationship to patient's condition when appropriate OR clinician does not ask about drug and alcohol use but this is appropriate to age and gender of patient OR clinician asks about alcohol or drug use in close family members |
| **11. Assessing Appropriate involvement of family member, significant other and caregiver** ☐ **Not applicable** | | |
| 1 | **Needs improvement** | = clinician only talks with the patient's family and does not give the appropriate respect for the feedback of the patient and ignores patient perspective, (e.g., "You should listen to your family more.)OR (if family not present) fails to ask the patient about the involvement of the family |
| 2 | **Done Partially** | = clinician ask about family involvement, but does not explore patient's reasons for involvement or non-involvement |
| 3 | **Done Well** | = clinician makes a treatment plan that considers the patient's perspective on how much they want family involvement (even if the family is not present)**and** encourages interaction between the two |
| **12. Assessing Collaborative goals setting and expectations of the patient for recovery** ☐ **Not applicable** | | |
| 1 | **Needs improvement** | = clinician does not ask patient about his/her goals for recovery **OR** clinician just tells patient what to do without asking his/her opinion |
| 2 | **Done Partially** | = clinician asks patient about goals for recovery but does not discuss if these are realistic or can be accomplished |
| 3 | **Done Well** | = clinician asks about goals regarding the treatment and discusses with patient what is and is not realistic and achievable through treatment; collaboratively clinician and patient establish treatment plan |
| **13. promotion of realistic hope for change** ☐ **Not applicable** | | |
| 1 | **Needs improvement** | = clinician either gives no hope (e.g. you will never get better) or gives unrealistic expectations (e.g. you will be cured in a few weeks and never have problems again) for what to expect in treatment and recovery |
| 2 | **Done Partially** | = clinician vaguely tells patient what will happen during treatment |
| 3 | **Done Well** | = clinician helps patient feel positive about the future **and** creates realistic expectations about what can and cannot be achieved through treatment and explains treatment checking patient understanding |
| **14. Assessing the usE of Local (ethnopsychological) termsin conducting psychoeducation** ☐ **Not applicable** | | |
| 1 | **Needs improvement** | = clinician uses technical jargon to explain about mental illness **OR** uses stigmatizing terms **OR** does not explain how treatment works |
| 2 | **Done Partially** | = clinician uses a limited amount of technical jargon but **No** stigmatizing terms |
| 3 | **Done Well** | = clinician conducts psychoeducation using local terminology and phrases to explain mental health and treatment in non-stigmatizing language, in a local language where appropriate, and checks to see if patient understands |
| **15. ASSESSING Problem Solving SKILLs: Problem Formulation & Prioritization, Solution Generation, Action Planning** ☐ **Not applicable** | | |
| 1 | **Needs improvement** | =Clinician attempts problem solving steps #2-4 (see below) but only completes 1 or 2 steps satisfactorily |
| 2 | **Done Partially** | =Clinician attempts problem solving steps #2-4 (see below) but only completes 3 steps satisfactorily |
| 3 | **Done Well** | =clinician helps patient to do all of the following (1) formulate and prioritize primary problem, (2) brainstorm solutions, (3) explores advantages and disadvantages, and (4) formulate action plan |

| 16. ASKING FOR FEEDBACK AND PROVIDING ADVICE, SUGGESTIONS AND RECOMMENDATIONS ☐ **Not applicable** | | |
|---|---|---|
| 1 | NEEDS IMPROVEMENT | = clinician lectures patient what to do without asking if this is acceptable and comfortable to patient, |
| 2 | DONE PARTIALLY | = clinician gives useful advice but does not ask for feedback from patient about the usefulness of the advice to the patient |
| 3 | DONE WELL | = clinician gives appropriate advice for the patient and explicitly asks for feedback about the usefulness of the advice |

| 17. CLINICIAN EXPLAINS CONFIDENTIALITY OF THEIR DISSCUSSION ☐ **Not applicable** | | |
|---|---|---|
| 1 | NEEDS IMPROVEMENT | = clinician does not address confidentiality (by explaining confidentiality or ensuring privacy) OR does not adjust conversation to setting (e.g. if other family members are present, does not take care with topics discussed) |
| 2 | DONE PARTIALLY | = clinician tells patient that everything is confidential with explaining harm to self or others |
| 3 | DONE WELL | = clinician explains that all clinician-patient discussions are confidential with the exception of harm to self and others OR ensures privacy OR adjusts conversation to setting |

| 18. HARM TO SELF, HARM TO OTHERS, AND HARM FROM OTHERS AND COLLABORATIVE RESPONSE PLAN ☐ **Not applicable** | | |
|---|---|---|
| 1 | NEEDS IMPROVEMENT | = clinician does not ask about harm to self or others or does not pick up on key signals of self harm or suicide |
| 2 | DONE PARTIALLY | = clinician asks about harm to self or others, but does not help patient to develop a crisis plan |
| 3 | DONE WELL | = clinician asks about harm to self or others and facilitates appropriate actions to assure safety |

| 19 ASSESSES ANTI-PSYCHOTIC MEDICATION ADHERENCE (TAKING MEDICATION APPROPRIATELY) ☐ **Not applicable** | | |
|---|---|---|
| 1 | NEEDS IMPROVEMENT | = Does not ask about anti-psychotic medication adherence OR reprimands patient for not taking medication appropriately |
| 2 | DONE PARTIALLY | = Asks about anti-psychotic medication adherence but does not ask him reasons for non-adherance, and/or does not explore ways to improve medication adherence |
| 3 | DONE WELL | = Asks about anti-psychotic medication adherence, and asks him reasons for non adherence, tries to understand reasons for these and explores ways to improve the situation |

| 20 ASSESSES FOR POTENTIAL ROLE OF COMMUNITY OR SOCIAL NETWORKS IN FINDING SOLUTIONS TO PROBLEMS ☐ **Not applicable** | | |
|---|---|---|
| 1 | NEEDS IMPROVEMENT | Does not assess the role of community or social networks as potential solutions to improve patient's situation |
| 2 | DONE PARTIALLY | Makes suggestions about role of community or social networks as potential solutions to improve patient's situationwithout asking for patient input |
| 3 | DONE WELL | makes appropriate suggestions and asks for feedback, about the role of strengthening community engagement or social networks as a potential solution to improve patient's situation |

| 21 ASSESSING PHYSICAL HEALTH ISSUES ☐ **Not applicable** | | |
|---|---|---|
| 1 | NEEDS IMPROVEMENT | = clinician does not ask about physical health and physical health issues |
| 2 | DONE PARTIALLY | = clinician takes partial history but does not explore positive responses about physical health issues |
| 3 | DONE WELL | = clinician assesses related physical health issues and explains relationship to patient's condition and lifestyle when appropriate |

| **Case difficulty rating (CIRCLE)** | |
|---|---|
| 1 | No complex issues or challenging behaviour |
| 2 | Some complex issues or some challenging behaviour |
| 3 | Very complex issues or very challenging behaviour |

| | |
|---|---|
| Total ENACT- E score | |
| Number of items rated | |
| Mean ENACT- E score | |

**Confirmation feedback given:**

Signatures of the assessor: ________________________ Date:_____________________

Signatures of person assessed____________________ Date:_____________________
