# Supplementary material for: “Like a doctor, like a brother”: Achieving competence amongst lay health workers delivering community-based rehabilitation for people with schizophrenia in Ethiopia
Source: PLoS One. 2021 Feb 25;16(2):e0246158. doi: 10.1371/journal.pone.0246158 (PMC7906313; doi:10.1371/journal.pone.0246158)
Supplement: Attachment — Submitted filename: Response to reviewers 09.12.20.docx [file pone.0246158.s004.docx]

9th December 2020

Dear Professor Wasserman,

**Re: PONE-D-20-30256 “Like a doctor, like a brother”: achieving competence amongst lay health workers delivering community-based rehabilitation for people with schizophrenia in Ethiopia**

**Response to reviewers’ comments**

Thank you for the opportunity to revise the above manuscript. Please find the responses below (note that line/page references refer to the tracked changes version of the manuscript).

| **Comment** | **Response** |
| --- | --- |
| **Journal requirements** | |
| Ensure that your manuscript meets PLOS ONE's style requirements, including those for file naming | Amendments have been made to meet the guidelines |
| Please provide additional information about your teaching intervention, such as detailed curriculum, description of texts or methods used, or supporting educational material that would allow others to replicate your study. | There is a summary of the RISE training programme in the methods lines 185-211, which we feel is appropriate detail for the body of the paper. I had provided a link to the 200 page RISE training manual, but I have now added this as an appendix instead. There is an overview of the manual contents in the main text.  S1 Appendix provides an overview of the RISE training programme. To this file I have added the following text signposting to the detailed training materials ‘Further training resources, including powerpoint slides and training activities in English and Amharic, are available on request [laura.asher@nottingham.ac.uk](mailto:laura.asher@nottingham.ac.uk)’. The training materials consist of 46 powerpoint presentations, each in English and Amharic, 32 handouts/exercises, plus videos and other materials, so it is not practical to link these directly to the paper. |
| Amend your list of authors on the manuscript to ensure that each author is linked to an affiliation. | Minor amendments made to author affiliations. Each author has at least one affiliation. |
| Include captions for your Supporting Information files at the end of your manuscript | These have been added |
| **Reviewer** |  |
| It would be interesting to know which proportion of the patients were on medication? | We have added details on the pilot participants including medication use, “*At recruitment into the pilot, half of participants were treatment naïve and only one participant was taking anti-psychotic medication. Several participants went on to take anti-psychotic medication for varying periods during the 12 month pilot study*”. See lines 251-261. |
| The legends of table 1 should mention the sample and explain ENACT, in order to make it possible to read the table without consulting the text in details. | We have amended the titles and footnotes of Tables 1 and 2. |
| If necessary due to space limitations, some of the cited phrases could be left out | We feel all the included quotes would ideally remain in the paper to adequately represent the rich qualitative data supporting our analysis. However if the editor feels the overall length needs reducing, we can omit some of these. |
| The main challenge will be to make sure that lay people do not practice work that is difficult or dangerous for themselves and for the patients. This could involve emotional overinvolvement, taking over too much responsibility, and ending up in a role like a relative. Lay people will be faced with unmet needs of the patients, and how to decide what to do? Procedures must make sure that the lay people can continue their own personal life outside working hours without too much worry and concern regarding their job. | We agree these are important challenges. We have added text to the methods to describe how CBR wellbeing and safety was assured (lines 238-240 and 269-273) : “*The training covered approaches to support CBR wellbeing, including keeping appropriate boundaries with participants, and how safety would be assessed and maintained*.” And “*All home visits were made with caregivers present and CBR workers were required to carry a mobile phone at all times. CBR supervisors made regular risk assessments of the home visit environment. Additional safety procedures, such as only permitting joint visits with supervisors, were implemented according to the assessment outcome*.”  We have also added reflections on these issues in the discussion, lines 845-850: “*Geographic and socio-cultural similarities between CBR workers and participants, whilst supporting a better experience for participants, may increase the likelihood of emotional over-involvement. Peer supervision is proposed as a useful approach to reduce stress and minimise feelings of inadequacy (17), and may also help lay workers to identify and address problems maintaining appropriate boundaries*.” And lines 910-911 “*Rigorous risk assessments and procedures to protect lay worker safety are also paramount*.” |
| Also, idiosyncratic practice where a lay person starts to work mainly guided by own opinion or therapeutic ideas should be addressed. It could also be considered to work in pairs in order to make sure that practice is visible? | We agree this is another important challenge and interesting potential solution- we have added reflections on this in the discussion lines 939-942 “*Observation of some home visits by a supporting CBR worker could also be a relatively resource-light means of ensuring broader intervention fidelity, that would have the additional benefit of minimising any safety concerns associated with lone visits*.”  In addition CBR supervisors did conduct unannounced observed home visits every two months (when ENACT assessements were completed), which is mentioned in the methods (lines 266-267). |

Kind regards

Laura Asher
